# Systematic Mapping of Homoserine Lactone and Cyclodextrin Binding Strengths — Effects of Structural Features

**DOI:** 10.1002/chem.202501916

**Published:** 2025-08-28

**Authors:** Otso I. V. Luotonen, Rasmus Rantanen, Lijo George, Sandra Kaabel, Eduardo Anaya‐Plaza, Mauri A. Kostiainen

**Affiliations:** ^1^ Department of Bioproducts and Biosystems Aalto University Espoo 0076 Aalto Finland; ^2^ Center of Excellence in Life‐Inspired Hybrid Materials (LIBER) Aalto University Espoo 0076 Aalto Finland; ^3^ Department of Chemistry and Materials Science Aalto University Espoo 0076 Aalto Finland

**Keywords:** cyclodextrin, homoserine lactone, host‐guest chemistry, supramolecular interaction, synthesis

## Abstract

Directing the collective behavior of bacteria is important for various applications in chemical bioproduction, water treatment, and antibiofilm solutions. A potential approach to such control mechanisms lies in sequestering signal molecules (autoinducers) by macrocyclic host molecules that lower the effective concentration of the former, modulating bacterial signaling. Cyclodextrins (CD) — one of the best‐established families of hosts — have been shown to bind homoserine lactones (HSL) acting as autoinducers, but with a focus limited to shorter (≤ 8 carbons) tailed molecules and β‐CD. Here, we have systematically mapped binding affinities for HSLs of three different tail lengths and with different 3‐site substituents (used for signal differentiation), with native and substituted α‐ and β‐CDs. The HSL alkyl chain length has the most influence on affinity, although the 3‐substitution also slightly affects the binding constant. Little difference between native CDs was observed, but the binding ability of α‐CD was more susceptible to even minute substitutions. The wider β‐CD core could be substituted with greater modularity without impairing HSL binding ability. The results constitute an initial chemical toolbox to be applied in guiding host design for HSL sequestering in, for example, therapeutic applications, but also in constructing systems for the modulation of bacterial collective behavior.

## Graphical abstract

Binding of homoserine lactone (HSL) signaling molecules used by Gram‐negative bacteria with native and substituted cyclodextrin (CD) host molecules was mapped. Between differently substituted HSLs with different alkyl chain lengths, chain length governed binding affinity. Between α‐CD and β‐CDs, the wider β‐CD cavity was found to be more tolerant of substitutions in terms of binding constant.

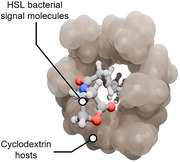

## 1 Introduction

The modulation of bacterial collective action presents potential for applications in various fields; certain behaviors may be encouraged for, for example, biosynthesis^[^ ^1^ ^]^ or wastewater treatment.^[^ ^2^ ^]^ Conversely, the blocking of biofilm formation has been explored for anti‐biofouling^[^ ^3^ ^]^ as well as anti‐virulent solutions with a lower risk of developing resistance.^[^ ^4^ ^]^ Bacterial quorum sensing (QS), a cell‐to‐cell communication system mediated by signaling molecules, regulates various processes in response to local cell density.^[^ ^5^, ^6^, ^7^ ^]^ Therefore, chemical interaction with the QS system provides an avenue to control their collective behavior.

**Figure 1:**
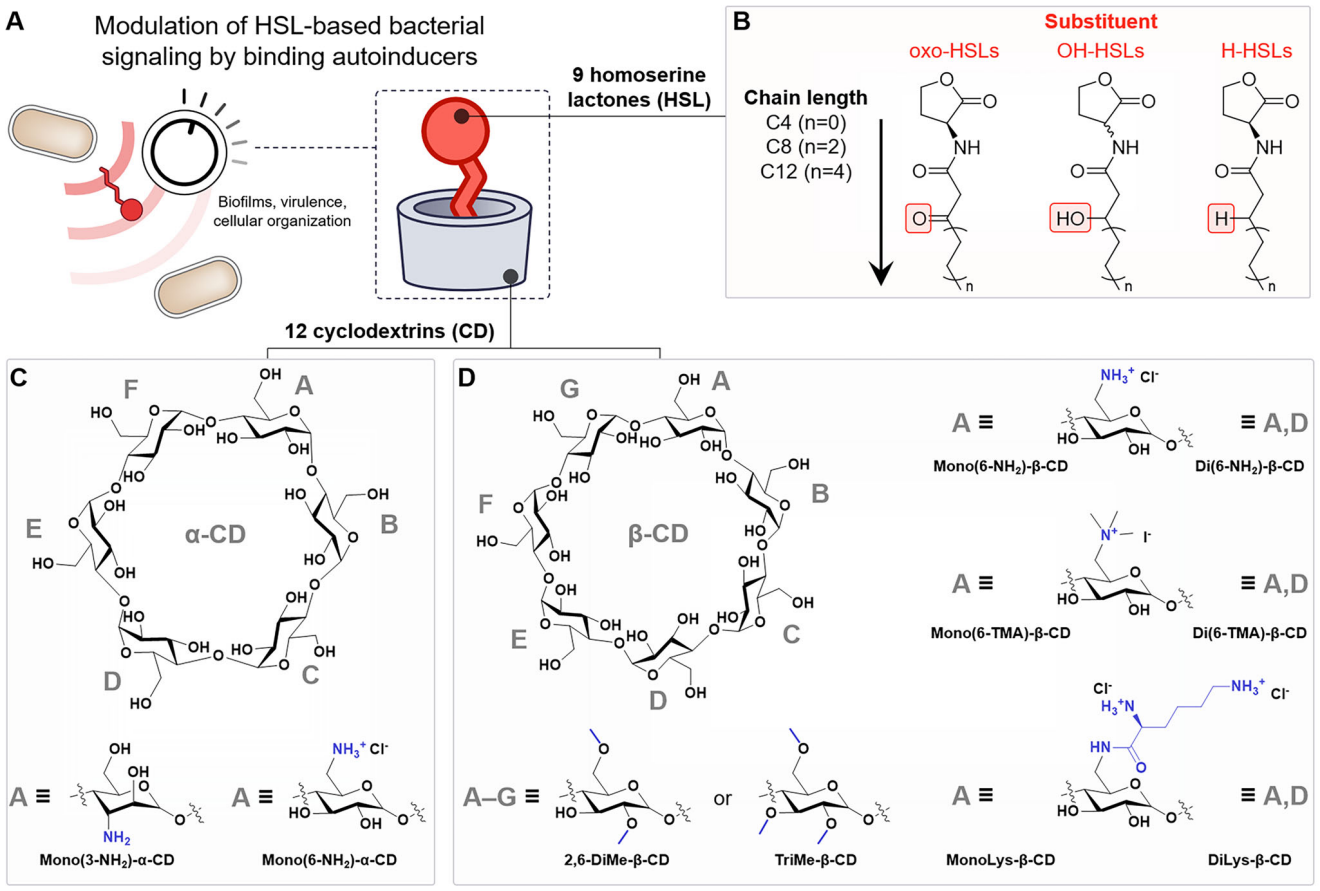
A) Schematic representation of HSL‐mediated quorum sensing (QS) and of an HSL autoinducer molecule complexed into the cavity of a CD host. B) Structural variations in the studied HSLs: three substituents on the 3‐site of the acyl chain and three different acyl chain lengths (calculated from the nitrogen atom onwards, C4, C8, or C12). Note that among the HSLs, OH‐C12‐HSL was purchased as a racemic mixture; the others were used as the l isomer. C) Structures of the native and substituted α‐CDs included in the study. D) Structures of the native and substituted β‐CDs included in the study.

One approach to modulating QS involves inactivation by complexation of signal molecules (called autoinducers), using macrocyclic “host” molecules characterized by cavities that can accommodate smaller “guest” molecules within. Among the different QS systems found among bacteria, N‐acyl homoserine lactones (AHL^[^ ^8^ ^]^ or HSL^[^ ^9^, ^10^ ^]^) are characterized by an HSL “head” and an acyl “tail,” ^[^ ^11^ ^]^ which can be targeted for binding^[^ ^4^, ^12^, ^13^ ^]^(Figure 1A). HSLs are a common autoinducer among Gram‐negative bacteria, including multiple clinically relevant strains.^[^ ^14^, ^15^, ^16^ ^]^ The use of cucurbiturils^[^ ^17^ ^]^ and recent efforts focused on pillararenes^[^ ^4^, ^13^ ^]^ have been reported, as well as cyclodextrins^[^ ^12^, ^18^ ^]^ (CD). Advantages of CDs as a host category include the native macrocycle's low toxicity^[^ ^19^ ^]^ and availability,^[^ ^20^ ^]^ which have led to their approved use in industry and use as a common model host. Native and substituted CDs have been shown to inhibit QS‐mediated pigment expression,^[^ ^18^, ^21^ ^]^ bioluminescence^[^ ^18^, ^22^, ^23^ ^]^ and enzyme activity.^[^ ^12^, ^18^ ^]^ In an earlier screening of various host molecules for binding HSLs, based on a fluorescence‐detected bacterial reporter system, α‐ and β‐cyclodextrins (α‐CD, β‐CD, respectively) showed a promising inhibition of HSL detection, alongside a cationic pillar[5]arene.^[^ ^4^ ^]^

Much of the work on the binding of HSLs with CDs has focused on relatively short‐tailed HSLs (C8 or less) and covers a limited number of HSLs as a binding target to characterize.^[^ ^12^, ^18^, ^22^, ^24^ ^]^ In particular, the possible effect of the 3‐site substituent for an HSL of a given length has received little attention. Among the three major native CDs (α, β, γ), β‐CD often garners more attention.^[^ ^18^, ^21^, ^24^ ^]^ In this work, we have systematically probed the binding ability of different CDs against HSLs with three different tail lengths (C4, C8, C12) and three different 3‐site substitutions (oxo, hydroxyl, and unsubstituted) (Figure 1A, B). Comparisons are drawn between the native α‐ and β‐CD, as well as different substituted forms of the two (Figure 1C, D). The HSL tail length governs the binding affinity, albeit a slight difference between different substitution motifs may be present — particularly for the shortest‐tailed autoinducers (C4), where the relative alkyl chain length is most affected. Between different diameter α‐ and β‐CD (6‐ and 7‐membered macrocycles, respectively), substitution was more likely to impair the former's binding affinity toward HSLs. The results present a chemical toolbox for informing the design of new macrocyclic hosts for the binding of HSLs in therapeutic applications, as well as in designing bioengineered systems that make use of reversible modulation of the QS system for, for example, biotechnological processes or synthetic biology.

## 2 Results and Discussion

### 2.1 Native CD Comparison

#### 2.1.1 NMR of α‐CD and β‐CD

First, the differences in HSL binding affinity between the differently sized native α‐CD and β‐CD were resolved using ^1^H NMR host–guest titrations. Titrations were carried out at room temperature in 1% DMSO‐d_6_/D_2_O, using HSL concentrations between 0.05–1 mM as dictated by compound solubilities, and adding increasing amounts of the selected host, up to [Host]/[HSL] ratios of ca. 10.

**Figure 2:**
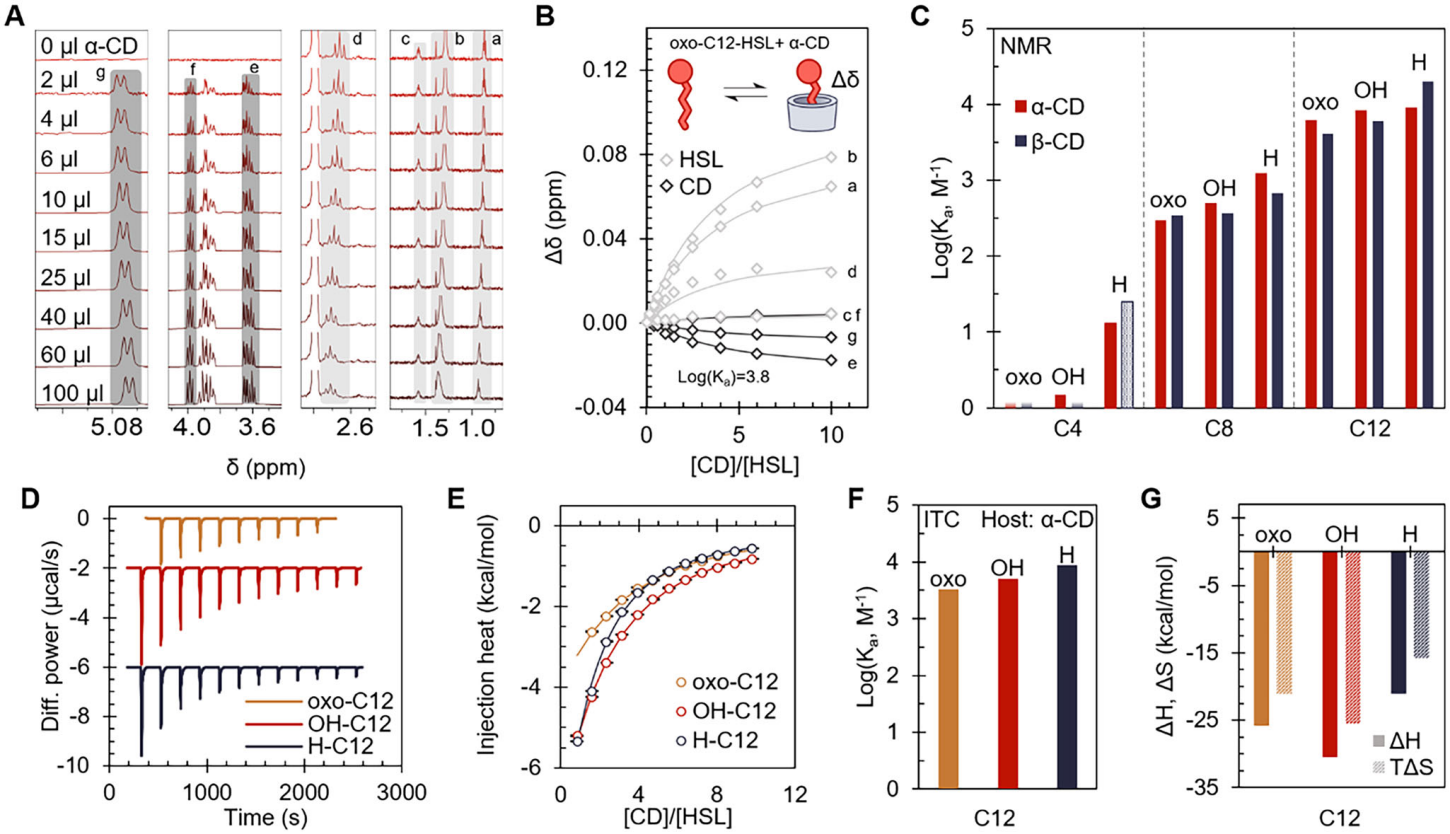
A) ^1^H NMR spectra of oxo‐C12‐HSL titrated with increasing α‐CD concentrations ([Host] = 3 mM, [HSL]_initial_ = 0.05 mM, V_initial_ = 600 µL). The lighter‐traced peaks correspond to the HSL, and the darker‐traced peaks to the CD. B) Traces of relative peak shifts as a function of [CD]/[HSL] over the oxo‐C12‐HSL versus α‐CD titration. Curves correspond to 1:1 binding fits at the experimental molar ratios. C) Binding constants obtained for native CDs versus the studied HSLs (via ^1^H NMR titrations, run as singlicates). The binding constant between β‐CD and H‐C4‐HSL is reported based on the work of Ikeda et al.  .^[^ ^12^ ^]^ The short gradient bars denote binding affinity deemed inconclusive or “very low,” based on very small observed shifts. D) ITC thermograms of α‐CD titrations versus C12‐HSLs (arbitrary baseline values for stacking of plots), run as singlicates. E) Integrated injection heats (error bars: estimated integration error^[^ ^35^ ^]^) as a function of [CD]/[HSL] for α‐CD titrations versus C12‐HSLs, along with traces following 1:1 binding fits through the experimental molar ratios. F) Binding constants obtained for α‐CD versus C12‐HSL pairs via ITC. G) Thermodynamic parameters obtained for α‐CD versus C12‐HSL pairs via ITC.

The chemical shifts of selected peaks (showing shifting and reliably trackable positions, mostly from HSL hydrogens) in each titration were fitted to a 1:1 binding model using the software Musketeer.^[^ ^25^ ^]^ The titration of oxo‐C12‐HSL with α‐CD is presented as an example (Figure 2A, B), and the calculated binding constants are summarized in Figure 2C. Spectra for the titrations, along with the peak trajectories and fitted curves, can be found in the Supplementary Information (Supporting Information Section 3.1).

The dominating effect of the HSL alkyl tail length on binding affinity is readily apparent, both for α‐CD and β‐CD. Among C4‐tailed HSLs, very minute shifts in δ were observed within the studied concentration range, particularly for the substituted variants (oxo and OH) (Supplementary Figures S5–7, S13–15). Accordingly, the fitting yielded the very low binding constant values of log(K_a_, M^−1^) – 0.06 and 0.18 for the α‐CD•oxo‐C4‐HSL and α‐CD•OH‐C4‐HSL pairs, respectively (Supplementary Figures S5–S6). For the α‐CD•H‐C4‐HSL pair, the shifts were larger in magnitude (still minute nonetheless), and a binding constant of log(K_a_, M^−1^) 1.1 was obtained (Supplementary Figure S7). For the corresponding β‐CD pairs, the lower titration concentrations arising from β‐CD's particularly poor solubility^[^ ^26^ ^]^ and its relation to the guest concentration (initial HSL concentration 0.05–0.15 mM) led to very small shifts and poorly fittable data (Supplementary Figure S15). However, the binding constants for H‐C4‐HSL and the two native CDs have been reported earlier by Ikeda et al.^[^ ^12^ ^]^ and are in agreement with the aforementioned constant with α‐CD: 1.3 and 1.4 for α‐CD and β‐CD respectively (^1^H NMR‐based titration). The results with C4‐HSLs generally show that they are not very effectively sequestered by α‐CD and β‐CD, but slightly higher affinities seem to exist for the unsubstituted H‐C4‐HSL.

With the longer C8‐tailed HSLs, more clear peak shifts were observed, with fitted binding constants ranging from 2.5 to 3.1 (log(K_a_, M^−1^)) (Supplementary Figures S8–10, S16–18). We note that the titration data for β‐CD pairs again shows smaller shifts due to the lower reachable concentrations within the solubility limits. The longest‐tailed C12‐HSLs produced the highest binding constants, in the range of 3.6 to 4.3 log(K_a_, M^−1^) (Supplementary Figures S11–12, S19–21). In other words, the studied hosts’ binding constants increased roughly by an order of magnitude with the addition of four carbons onto an HSL's alkyl chain. A ROESY NMR experiment of α‐CD and H‐C8‐HSL was also performed, showing through‐space correlations between the hydrophobic alkyl tail and the host's inner hydrogens (Supplementary Figure S57). The trend of HSLs with longer alkyl chains being bound more strongly — together with the localization of the chain inside the host cavity — agrees with chemical intuition. The CD cavity, typically described as “hydrophobic”^[^ ^27^ ^]^ or “semipolar”^[^ ^28^ ^]^ is well known to host hydrophobic guests, and the solvation of longer‐tailed HSLs in aqueous media is expected to be more disadvantageous, contributing to greater hydrophobic driving force. Additionally, the shortest HSLs may not be able to fully release high‐energy water molecules from within the CD cavity, a mechanism that has been presented as contributing to CD binding^[^ ^28^, ^29^ ^]^ (albeit weakened relative to some other host families due to partial stabilization of cavity water^[^ ^30^ ^]^).

While hydrophobic behavior seems to dominate the studied systems’ overall behavior, the availability of additional interactions between CDs and guests should be borne in mind. The potential contribution of van der Waals forces has been noted,^[^ ^28^, ^30^ ^]^ and improved binding of alkyl chains when terminated with polar groups has been linked to guest–rim interactions (dipole–dipole, hydrogen bonding)  .^[^ ^28^ ^]^ The latter is indicated by a small but consistent trend showing that for both α‐CD and β‐CD, and for all HSL lengths, the binding constants for unsubstituted HSLs were slightly higher than for HSLs with an OH‐ or oxo‐substituent on the 3‐site. These relative binding constants also concord with the lower aqueous solubility and critical micellar concentration of H‐C12‐HSL.^[^ ^31^ ^]^

Despite the different host cavity diameters, pairs involving α‐CD and β‐CD give mutually similar association constants in general. This similarity is intriguing when compared to the differing responses of a previously reported fluorescence‐detected *Escherichia coli* reporter system to α‐CD and β‐CD.^[^ ^4^ ^]^ The results showed a somewhat stronger effect of α‐CD on reporters for oxo‐C12‐HSL and an OH‐substituted C14‐tailed HSL, while β‐CD elicited an earlier response on an H‐C4‐HSL detector. Additional results have been described in a series of recent reports on QS microbial models.^[^ ^23^, ^32^, ^33^, ^34^ ^]^ The bioluminescence of *A. fischeri* and pigment production of *P. aeruginosa* could be inhibited to various degrees with native and substituted forms of α‐ and β‐CDs, with the α‐CDs generally performing somewhat better. Such disparity in effect on a biological system, regardless of the similar binding constants, points to differences in binding behavior of the differently substituted HSLs with the α‐CD and β‐CD. A larger number of other species may be able to compete with HSLs in the case of the wider β‐CD cavity, hindering the latter's effective activity.

#### 2.1.2 ITC of α‐CD

Secondly, isothermal titration calorimetry (ITC) measurements were carried out with α‐CD to provide more insight into the driving forces governing the system (Figure 2D, E). ITC titrations were performed at 25 °C in 1% DMSO/H_2_O (details of calorimetric procedures are described in Section 2 of Supplementary Information). With the explored concentrations, C12‐HSLs (Figure 2F) and C8‐HSLs (Supplementary Figure S28) yielded results in good agreement with those obtained from ^1^H NMR measurements in terms of fitted binding constants, save for H‐C8‐HSL (likely due to adsorption onto the ITC cell surface; see Supplementary Results and Discussion). Within the set of differently substituted C12‐HSLs, there is no dramatic difference between fitted thermodynamic parameters, with exothermic signatures and considerable entropic penalty (Figure 2G). The binding is enthalpically favorable but entails a considerable entropic penalty, corresponding with a “nonclassical hydrophobic effect.” ^[^ ^28^, ^29^ ^]^ These parameters may be rationalized as “frustrated” water molecules being freed into solution to form more hydrogen bonds^[^ ^30^ ^]^; along with a penalty from the HSL and/or host's conformational restrictions, not fully compensated by the shedding of low‐entropy water. Though minute, the differences between C12‐HSLs may be explained by hydrogen bond formation between host and guest adding to the enthalpy of binding, and H‐C12‐HSLs binding relieving surrounding water molecules’ structuring to a higher degree.

For C8‐HSL titrations, the fitted enthalpic changes of binding were considerably weaker but still favorable. Differing from other ITC titrations, the fitted entropic component for oxo‐C8‐HSL and OH‐C8‐HSL was slightly positive (i.e., contributing to binding). The differences in thermodynamic parameters between C12‐HSLs and C8‐HSLs can be interpreted as a stronger “nonclassical” hydrophobic effect (enthalpic driving force) being present for the longer‐tailed C12‐HSLs, partially offset by a more penalizing loss of conformational freedom of the same alkyl tail upon binding.

### 2.2 Effects of Substituents on CDs

**Figure 3:**
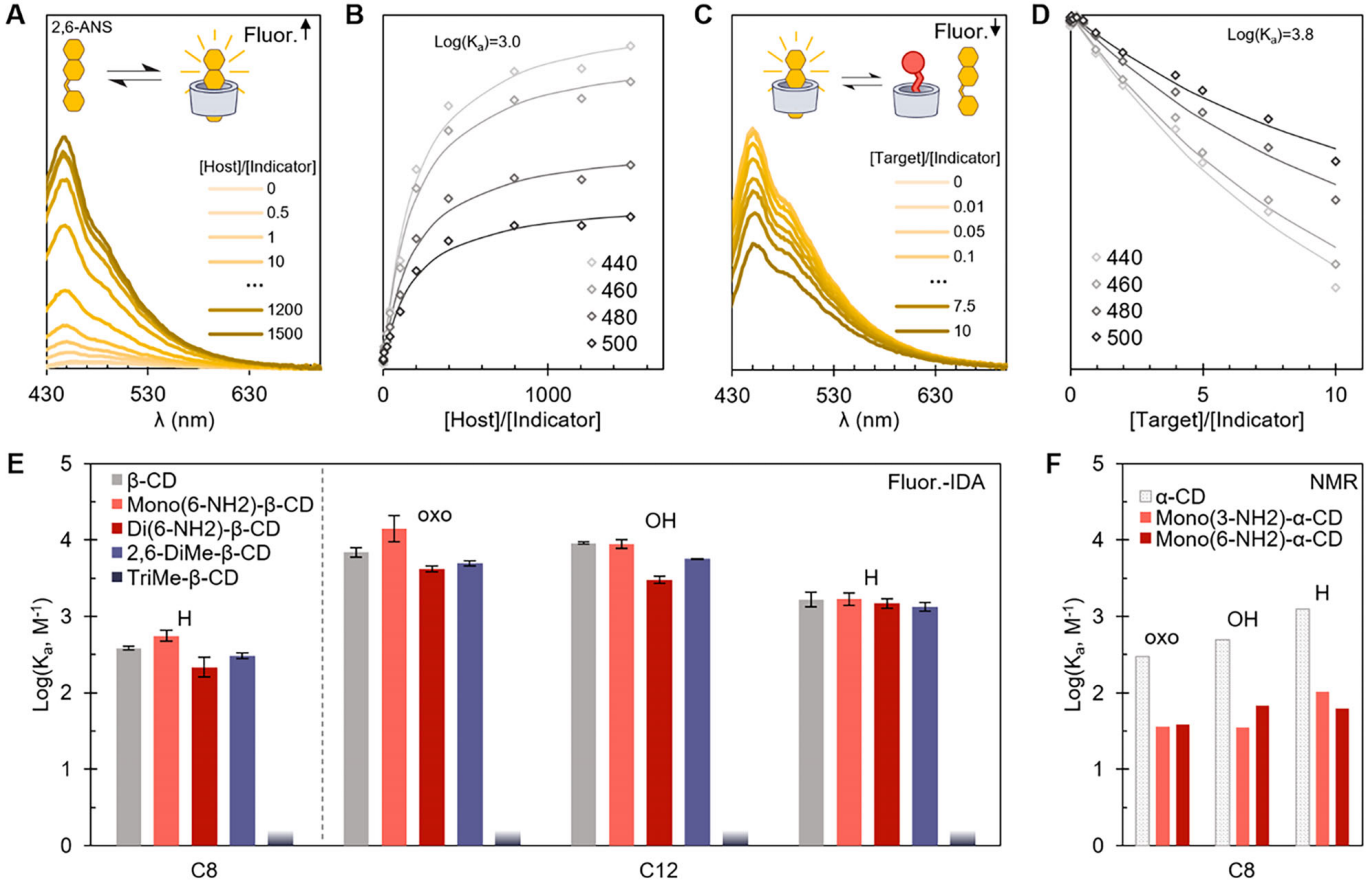
A) Fluorescence spectra of 2,6‐ANS titrated with increasing β‐CD concentrations. B) Traces of relative fluorescence intensity change as a function of [CD]/[2,6‐ANS] over the titration for given wavelengths. Curves correspond to 1:1 binding fits at the experimental molar ratios. C) Fluorescence spectra of IDA of oxo‐C12‐HSL against β‐CD and 2,6‐ANS. D) Traces of relative fluorescence intensity change as a function of [oxo‐C12‐HSL]/[2,6‐ANS] over the displacement assay for given wavelengths. Curves correspond to a 1:1 binding fit at the experimental molar ratios. E) Binding constants obtained via fluorescence IDAs for native and substituted β‐CD (error bars: standard deviation of separately fitted triplicate measurements). The short gradient bars denote binding affinity deemed inconclusive or “very low,” based on weak observed shifts. F) Binding constants obtained via ^1^H NMR titrations for native and substituted α‐CD (constants for native α‐CD repeated from Figure. 2), run as singlicates.

In addition to the comparison of the native α‐CD and β‐CD in binding HSLs with different substituents and chain lengths, the effects of various substitutions on the CD structure were explored (Figure 3). Different substituents can be used to functionalize CDs, to adjust their cavity properties and thus binding affinity, or to include additional functionalities to the molecule (e.g., biologically active moieties). However, functionalization may also impair a host's binding ability. In addition to the neutral hosts, cationic variants were chosen, based on their exploration for antibacterial and antibiofilm functionality that may find synergy with the control of QS in antibiofilm and antivirulence applications.^[^ ^4^, ^36^, ^37^ ^]^

A UV–Vis or fluorescence‐based indicator displacement assay (IDA) can lend itself well to such exploratory work covering larger numbers of combinations. For β‐CDs, the fluorescent compound 2‐anilinonaphthalene‐6‐sulfonic acid (2,6‐ANS) is a suitable indicator, being water‐soluble and increasing its fluorescence upon inclusion (Figure 3A)  .^[^ ^38^ ^]^ However, a considerable screening effort of available dyes did not yield suitable indicators for use with α‐CDs, likely due to their narrower cavity preventing full inclusion of chromophores or fluorophores typically bearing aromatic rings. As such, the native CD and their substituted forms were characterized via ^1^H NMR titrations for α‐CDs and via fluorescence titrations for β‐CDs (at room temperature in 1%DMSO/H_2_O using 2,6‐ANS as an indicator, see Supplementary Figures S30–S38; the binding study against oxo‐C12‐HSL is illustrated as an example in Figure 3C–D).

#### 2.2.1 α‐CD Substituted

In the case of α‐CDs, two mono‐aminated hosts were chosen for study, substituted at either the 6‐site or 3‐site of the glucopyranoside subunits (Figure 1C). The binding of C8‐HSLs was characterized for these macrocycles (Figure 3F, Supplementary Figure S22–S27). Compared to native α‐CD, the aminated derivatives show an impaired affinity with binding constants lowered by close to an order of magnitude (for instance, log(Ka) = 3.1 for α‐CD•H‐C8‐HSL and 2.0 for mono(3‐NH_2_)‐α‐CD•H‐C8‐HSL). The mono‐amination may crowd the CD rim to a slightly increased degree, but the overlap with the cavity entrance would seem minimal. Other possibilities for the hindered binding might include a conformational twisting of the overall α‐CD macrocycle structure that leads to a more obstructed fit. Mono(3‐NH_2_)‐α‐CD showed a similar minute increase of fitted binding constant for the unsubstituted H‐C8‐HSL as α‐CD (2.0 vs. 1.6, logarithmic, for both), but the same effect is not visible for mono(6‐NH_2_)‐α‐CD (log(K_a_) = 1.8 for H‐C8‐HSL, 1.8 for OH‐C8‐HSL, and 1.6 for oxo‐C8‐HSL).

#### 2.2.2 β‐CD Substituted

For β‐CDs, a larger variety of substituents is accessible through synthesis, including mono‐ and di‐substituted β‐CDs with lysine and trimethylammonium (TMA) moieties (see Supporting Information Section 2 for synthesis and characterization). Comparing the use of 2,6‐ANS for IDA experiments with previous work, the association constant fitted with β‐CD agrees well with reported values (reported log(K_a_) 3.1 vs. our 3.0).^[^ ^38^ ^]^ Constant concentrations of host and indicator were titrated with increasing concentrations of HSL as a displacement experiment (see Supporting Information Section 3.3.2).

The effect of tail length on binding constant is again apparent in the results, but a difference in the effect of host substituents can be seen in contrast with the results for α‐CDs. Compared to the hindered binding observed with amino‐substituted α‐CDs, aminated and di‐methylated β‐CDs seem less impacted by the substitution. Similar results were obtained with both of the lysine‐modified β‐CDs and their mono‐TMA‐modified counterpart (see Supporting Information Section 3.3.2). However, lower affinities were observed for trimethylated β‐CD compared to other β‐CDs — only slight decreases in fluorescence took place in IDAs; the results could not be fitted satisfactorily and were interpreted as a lowered binding affinity. The apparent lower affinity of trimethylated β‐CD for HSLs may be due to an overly crowded cavity due to the maximum amount of substituted methyl groups across the structure.

Among the substituted β‐CDs, the lysine‐ and TMA‐modified macrocycles proved more challenging in the host–indicator titrations due to low solubilities, limiting a full titration toward an endpoint, which would be ideal for the fitting of the data. As such, the characterization of these pairs — and by extension corresponding IDAs — was considered more approximative. Similarly, the shorter‐tailed C4‐HSLs showed very little or no effect in the displacement assays, preventing fitting and indicating only a binding constant lower than what this assay can cover. The reliably fittable pairs’ binding constants are presented in Figure 3E.

The reliability of binding constant determination via host–guest titrations can generally be verified by using multiple independent characterization methods. In our case, the ^1^H NMR and IDA titrations of β‐CD concord with each other otherwise, but a marked difference arises for H‐C12‐HSL. This is likely related to the unexpected behavior in the IDAs of said HSL, the addition of which led to a gradual increase in fluorescence in the case of lysinated and TMA‐substituted β‐CDs (Supplementary Figures S42–S45). If such a fluorescence increase is also produced to a smaller degree in the other H‐C12‐HSL IDAs, the decrease in fluorescence used to probe the system may be partially obscured, which may explain the lower‐than‐expected binding constants when compared to ^1^H NMR‐derived values for β‐CD.

## 3 Conclusion

The intelligent steering of bacterial community behavior may find uses in various fields, such as biosynthesis, biofilm repelling, and anti‐virulence. The QS system governs this collective action in many strains, and the HSLs commonly used by Gram‐negative bacteria present an element for modulation. HSLs can be supramolecularly bound by macrocyclic hosts such as CD. Here, we have systematically mapped the chemistry of both host and guest molecules. On one hand, we evaluated the effect of length and chemistry of the HSLs by screening nine HSLs — having three different acyl chain lengths (C4, C8, C12) and three different 3‐site substituents (oxo, OH, and H, i.e., unsubstituted). On the other hand, 12 different hosts — both native and substituted α‐CDs and β‐CDs — were evaluated. Advantages of CDs include being well established and commercially accessible, as well as having an established modification chemistry of the native macrocycle structure for increased functionality. For the binding of HSLs with these host molecules, chain length is generally the main deciding factor for the binding affinity. The effect of 3‐site substitution is clearly more minute, but the results suggest the unsubstituted HSLs might have slightly increased affinities toward the studied hosts. The substituted β‐CDs mostly showed negligible changes in binding affinity relative to the native form, pointing to the possibility of modularly adding functionalities on either side of the macrocycle without foregoing binding ability. The substituted α‐CDs were more impaired in terms of binding strength, suggesting a more sensitive binding motif that necessitates care if it is to be substituted for multifunctionality including HSL binding. The findings may find use in the design of HSL binders for the modulation of QS, as well as informing other applications of bacterial group behavior modulation.

## Conflict of Interest

The authors declare no conflict of interest.

## Supporting information



Supporting Information

## Data Availability

The data that support the findings of this study are available in the supplementary material of this article.
