# Supplementary material for: Systematic Mapping of Homoserine Lactone and Cyclodextrin Binding Strengths — Effects of Structural Features
Source: Chemistry. 2025 Aug 28;31(61):e01916. doi: 10.1002/chem.202501916 (PMC12587022; doi:10.1002/chem.202501916)
Supplement: Supplementary file 1 — Supporting Information [file CHEM-31-e01916-s001.pdf]

Systematic Mapping of Homoserine Lactone and Cyclodextrin Binding Strengths — Effects of Structural Features

Otso Luotonen[1,3], Rasmus Rantanen[1,3], Lijo George[1,3], Sandra Kaabel[2,3], Eduardo Anaya[1,3,*], Mauri A. Kostiainen[1,3,*]

[1] Department of Bioproducts and Biosystems, Aalto University, 0076 Aalto, Finland.

[2] Department of Chemistry and Materials Science, Aalto University, 0076 Aalto, Finland.

[3] Center of Excellence in Life-Inspired Hybrid Materials (LIBER), Aalto University, 0076 Aalto, Finland

* Corresponding author

Email addresses: mauri.kostiainen@aalto.fi, eduardo.anaya@aalto.fi

Supplementary Information

1. Contents

1. Contents S1
2. Materials and Methods S2
 2.1. Syntheses S4
  2.1.1. MonoLys-β-CD S4
  2.1.2. DiLys-β-CD S6
  2.1.3. Mono(6-TMA)-β-CD S8
  2.1.4. Di(6-TMA)-β-CD S9
3. Supplementary results and discussion S11
 3.1. NMR Detected Titrations S11
  3.1.1. With α-CD as a host S11
  3.1.2. With β-CD as a host S18
  3.1.3. With aminated α-CDs as hosts S28
 3.2. Isothermal Titration Calorimetry S34
 3.3. Fluorescence Detected titrations S36
  3.3.1. Indicator titrations S36
  3.3.2. Indicator displacement assays S43
4. References S63

2. Materials and Methods

Commercially available compounds were used as received, unless otherwise stated.

Homoserine lactones (HSLs). OH-C12-HSL (*N*-3-hydroxydodecanoyl-DL-homoserine lactone) was purchased from Sigma Aldrich. Other HSLs were purchased from Cayman Chemical: oxo-C4-HSL (*N*-3-oxo-butyryl-L-homoserine lactone), OH-C4-HSL (*N*-3-hydroxybutyryl-L-homoserine lactone), C4-HSL (*N*-butyryl-L-homoserine lactone), oxo-C8-HSL (*N*-3-oxo-octanoyl-L-homoserine lactone), OH-C8-HSL (*N*-3-hydroxyoctanoyl-L-homoserine lactone), C8-HSL (*N*-octanoyl-L-homoserine lactone), oxo-C12-HSL (*N*-3-oxo-dodecanoyl-L-homoserine lactone), C12-HSL (*N*-dodecanoyl-L-homoserine lactone).

Cyclodextrins (CDs). α-cyclodextrin (α-CD); β-cyclodextrin (β-CD); 6-Monoamino-6-monodeoxy-α-Cyclodextrin (Mono(6-NH_2)-α-CD); 6-Monoamino-6-monodeoxy-β-Cyclodextrin (Mono(6-NH_2)-β-CD); and A,D-6-Diamino-6-dideoxy-β-Cyclodextrin (Di(6-NH_2)-β-CD) were purchased from Cyclodextrin-shop (Netherlands). 3A-Amino-3A-deoxy-(2AS,3AS)-α-cyclodextrin (Mono(3-NH_2)-α-CD); 2,6-Di-O-methyl-β-cyclodextrin (2,6-DiMe-β-CD); Trimethyl-β-cyclodextrin (TriMe-β-CD) were purchased from Tokyo Chemical Industries (TCI).

Fluorescence detected indicator displacement assays (IDA). Indicator displacement assays were carried out using 2-anilinonaphthalene-6-sulfonic acid (2,6-ANS, Synchem, Germany) as an indicator in 1% DMSO/water (DMSO from Sigma Aldrich) at room temperature, with 200 µL sample volumes. Spectra were measured on Biotek Cytation 3 and Synergy H1 plate readers on transparent 96-well plates (Thermo Fisher) and blank subtracted (λ_{ex}=325 nm). Binding constants between 2,6-ANS and hosts were determined by titrating the indicator at constant concentration (between 1 and 20 µM) with increasing concentrations of host (up to [Host]/[Indicator] ratios of 140 to 5000). Binding constants between hosts and HSLs were then determined by titrating indicator and host at constant concentration (20 µM, and between 20 and 100 µM respectively) with increasing HSL concentration (up to [HSL]/[Indicator] ratios of 10 to 50). Association constants were obtained with Musketeer software[1]; indicator titration data were fitted to a 1:1 binding model and displacement data were fitted to competing 1:1 binding of indicator-host and HSL-host pairs. First, coarse singlicate survey titrations were carried out both in indicator titrations and in IDA titrations, followed by triplicate titrations if shifting signals could be seen within the available concentration window (both certain longer-tailed HSLs and substituted β-CDs showed prohibitively low solubilities for complete titrations to a saturated plateau). In case of multiplicates the spectra were fitted separately per replicate. The binding constants are reported as averages of the fits, along with standard deviation calculated for the logarithmic form as:

$$\sigma_{\log{(K_a)}} = 0.434\frac{\sigma_{K_a}}{K_a}$$

Nuclear magnetic resonance (NMR) detected host-guest titrations. Host-guest titrations detected with NMR were carried out on a Bruker AV NEO 600 for 600 MHz measurements and a Bruker AV NEO 400 for 400 MHz measurements, using 1% DMSO-d_6/D_2O as solvent, at room temperature. Titrations concerning C12-HSLs and/or β-CD were measured on the 600 MHz device due to the compounds' low solubilities. Deuterated solvents were purchased from Eurisotop and Sigma-Aldrich. Chemical shifts were calibrated relative to the water solvent peak (δ = 4.79 ppm). Spectra were measured for a constant amount of target HSL (initially between 0.05 and 1 mM, corrected for the addition of host solution) with an increasing concentration of cyclodextrin (up to a

[Host]/[HSL] ratio of ca. 10). Association constants were obtained with Musketeer[1] by tracking the shift of peaks upon host addition, fitted to a 1:1 binding model.

Isothermal titration calorimetry (ITC). ITC was performed at 25°C using a GE MicroCal iTC 200 microcalorimeter, using degassed (sonicated for 1 h) deionized water. Samples were prepared in 1% DMSO/H_2O with 10 µL and 1000 µL micropipettes (Eppendorf) which were not readjusted in volume within the same sample pairs, in order to minimize solvent mismatch. Samples were filtered through 0.23 µm PTFE syringe filters (Merck) and degassed for 5 min prior to analysis using a Malvern Thermovac. The syringe was cleaned with water and methanol between runs. The cell was cleaned routinely using 14% Decon90 (Decon Laboratories), water, and methanol (Honeywell). A more thorough cleaning with 1 M NaOH (Sigma-Aldrich) was carried out when deemed necessary, based on a lowered baseline in solvent-to-solvent control titrations, performed between sample runs. The software NITPIC[2,3] was used to integrate the thermograms, and SEDPHAT[4] was used fit ITC data to obtain binding constants as well as enthalpic and entropic contributions to the binding free energy.

High-resolution mass spectrometry (HRMS). Mass spectra were recorded with electrospray ionization, on an Agilent 6530 Q-TOF in positive ion mode using H_2O:ACN 2:1 as solvent. Minimal amounts of formic acid were added to the lysinated β-CD samples to enhance ionization.

2.1. Syntheses

2.1.1. MonoLys-β-CD

517 mg of DiBoc-lysine DCHA salt (Sigma-Aldrich) was dissolved into 50 mL DCM (Honeywell). The solution was washed three times with 30 mL of 2 M $NaHSO_4$ (aq, Merck), then with 30 mL brine. The organic layer was dried over sodium sulfate (Merck) and filtered, then evaporated under vacuum to yield the DiBoc-lysine as a clear solid.

207 mg (0.60 mmol) of DiBoc-lysine was dissolved into 16 mL of DMF (Fluka) in a round-bottom flask, followed by addition of 51 mg (0.38 mmol) of 1-Hydroxybenzotriazole (HOBt, Fluka). The reaction mixture was deoxygenated by argon bubbling, and 10 drops of *N*-methylmorpholine (NMM, TCI) were added, followed by 69 mg (0.36 mmol) of *N*-(3-Dimethylaminopropyl)-*N'*-ethylcarbodiimide hydrochloride (EDC·HCl, Sigma-Aldrich). After 15 min, 200 mg (0.176 mmol) of mono(6-NH_2)-β-CD·HCl was added, previously dissolved in 4 mL of deaerated DMF. The reaction was carried out at room temperature for 70 h, after which the solvent was evaporated under vacuum to yield a yellow, slightly sticky crust. This crude was crushed under sonication and washed with a total of 50 mL EtOAc (Honeywell). The retentate was purified by silica (Sigma-Aldrich) chromatography column, using 1-BuOH (Honeywell)/EtOH (Anora)/H_2O 5:4:3 as mobile phase. Fractions were combined based on TLC results with a ninhydrin (Sigma-Aldrich) stain. Given the high polarity of solvents and partial solubility of silica, the product was redissolved into MeOH (Honeywell) and filtered through a 0.2 µm PVDF (Merck) filter, before evaporation under vacuum. This produced a white? Solid, with a mass of 42 mg (17% yield).

To deprotect the intermediate product, 30 mg of product was dissolved into MeOH (1 mL) and placed in an ice bath, and acetyl chloride (285 µL, Sigma Aldrich) was added dropwise to generate HCl in situ as described by Nudelman et al[5]. . After all acetyl chloride had been added, the reaction was allowed to return to RT and react over 5 h. The reaction was then thrice diluted with another 2 mL MeOH and evaporated back to 1 mL, before fully evaporating to dryness and scraping off into a vial for a final mass of 22 mg (80% yield).

^{1}H NMR(400 MHz, CD_3OD): δ 5.18 (m, 1H), δ 4.97 (m, 7H), δ 4.0–3.6 (m, 28H), δ 3.6–3.4 (m, 14H), δ 2.95 (t, 2H), δ 1.90 (m, 2H), δ 1.71 (m, 2H), δ 1.49 (m, 2H). **HRMS**(ESI+): for $C_{48}H_{83}N_3O_{35}$; $[M + 2H]^{2+}$, calcd. m/z 631.7476 (100%), found 631.7490 (100%); $[M + H]^+$, calcd. m/z 1262.4880 (100%), found 1262.4866 (100%).

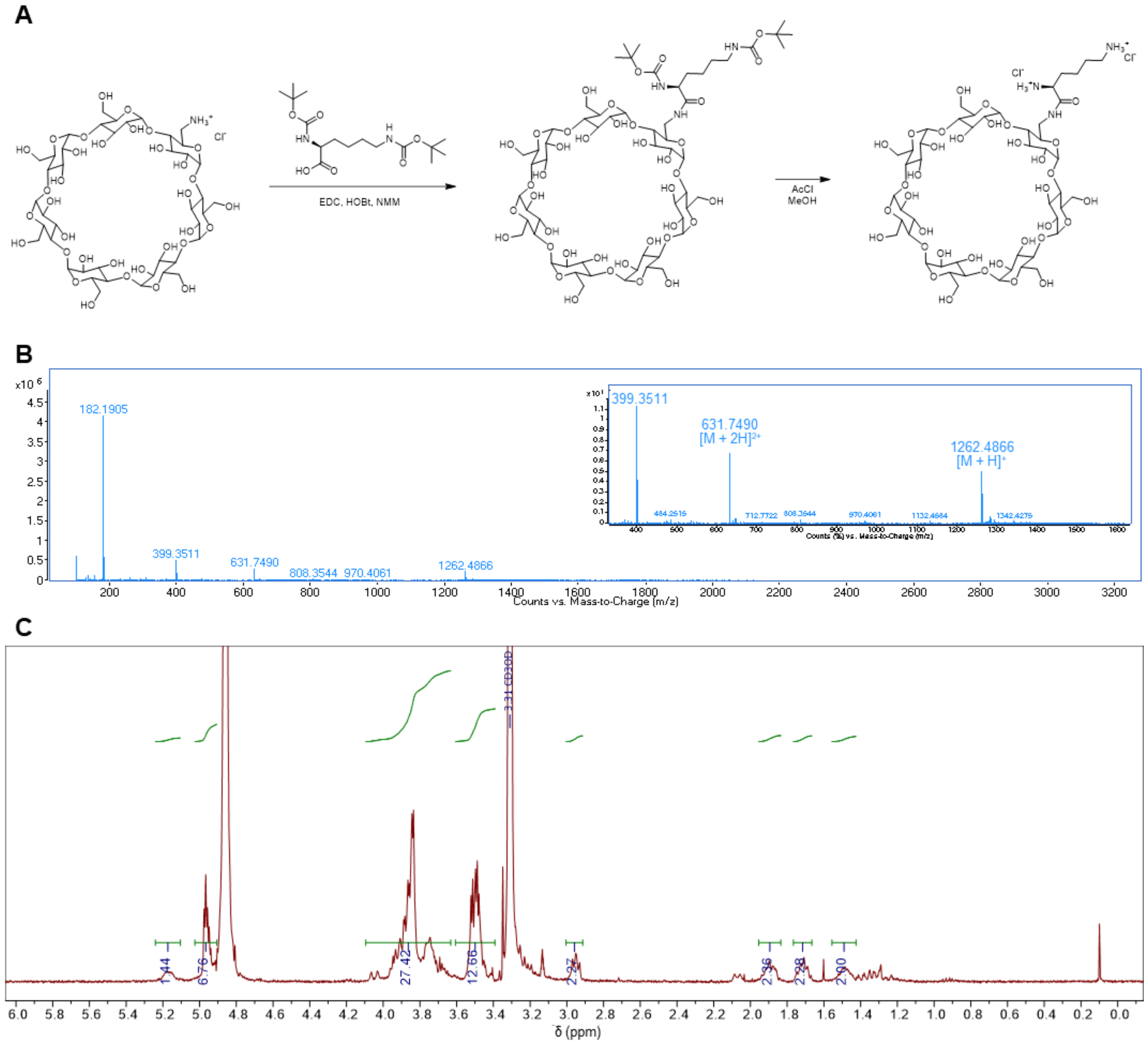

Figure S 1. **A** Synthesis of MonoLys-β-CD. **B** HRMS spectrum of MonoLys-β-CD. **C** ^{1}H NMR spectrum of MonoLys-β-CD.

2.1.2. DiLys-β-CD

132 mg of DiBoc-lysine DCHA salt (Sigma-Aldrich) was dissolved into 20 mL DCM. The solution was washed three times with 20 mL of 2 M NaHSO4 (aq), followed by three washed with 20 mL water, and finally with 20 mL brine. The organic layer was then evaporated under vacuum to yield the DiBoc-lysine as a clear solid (100 mg).

32 mg (0.17 mmol) of EDC·HCl was dissolved into 1.5 mL of DMF deaerated by nitrogen bubbling and cooled in an ice bath. 86 mg (0.25 mmol) of the previously prepared DiBoc-lysine dissolved in 300 µL of DMF was added, followed by 22 mg of HOBt (0.16 mmol). The solution was stirred for 1 min before adding 50 mg of di(6-NH_2)-β-CD·HCl (0.04 mmol) followed by 10 drops of NMM. The reaction was stirred in the ice bath for 30 minutes and allowed to proceed overnight at room temperature. After completion, 20 mL of diethyl ether () was added to the reaction mixture and the resulting product was filtered off. The product was again washed with 20 mL of diethyl ether, then 5 mL H_2O/ACN 1:8 twice. This yielded 30 mg (41% yield) of Boc-protected intermediate.

To deprotect the intermediate product, all of the intermediate was dissolved into 1 mL MeOH and placed in an ice bath, and acetyl chloride (570 µL) was added dropwise. The reaction was then allowed to return to RT and react 5 h. The reaction was then thrice diluted with 2 mL of MeOH and evaporated under vacuum to 1 mL, before fully evaporating to dryness to yield 25 mg of final product (96% yield).

^{1}H NMR(400 MHz, D_2O): δ 5.58–4.87 (m, 7H), δ 4.11–3.49 (m, 42H), δ 3.02 (t, 4H), δ 1.94 (m, 4H), δ 1.73 (q, 4H), δ 1.47 (q, 4H). **HRMS**(ESI+): for $C_{54}H_{96}N_6O_{35}$; $[M + 2H]^{+2}$, calcd. m/z 695.3031 (100%), found 695.3038 (100%); $[M + H]^+$, calcd. m/z 1389.5989 (100%), found 1389.5963 (100%).

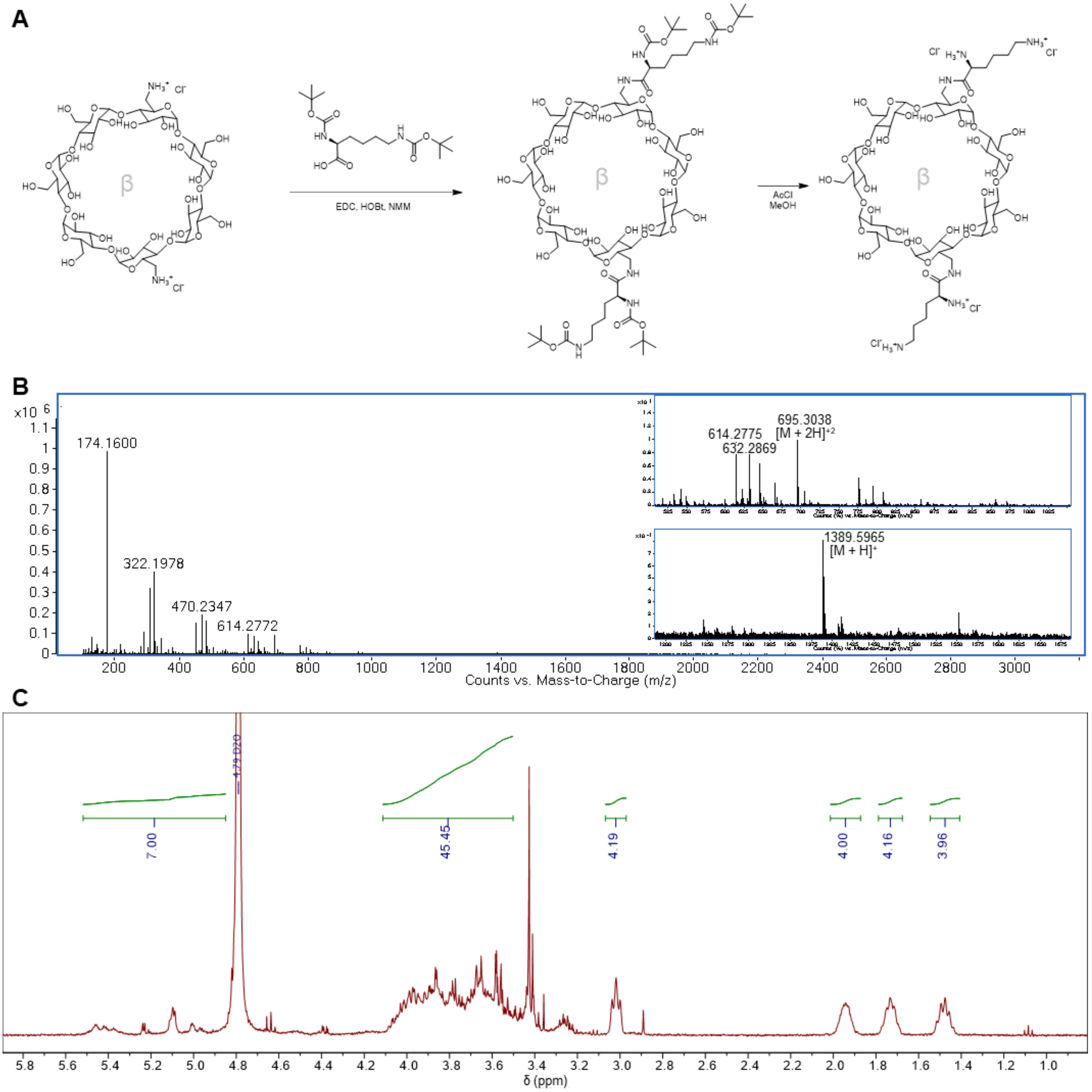

Figure S 2. **A** Synthesis of DiLys-β-CD. **B** HRMS spectrum of DiLys-β-CD. **C** ^{1}H NMR spectrum of DiLys-β-CD.

2.1.3. Mono(6-TMA)-β-CD

A 10 mL round-bottom flask equipped with a magnetic stirrer was placed under a nitrogen atmosphere and charged with 20 mg mono(6-NH_2)-β-CD·HCl (0.018 mmol). Subsequently, 2 mL of MeOH, dried over 3 Å molecular sieves, was added. 0.1 ml of methyl iodide (Sigma-Aldrich) and 6 mg (0.071 mmol) of anhydrous sodium bicarbonate (Sigma-Aldrich) were introduced into the solution, and the mixture was refluxed for 35 hours with additional portions of methyl iodide (0.1 ml) added at 15-hour and 20-hour intervals. Upon completion, the reaction mixture was diluted with 10 ml methanol, filtered, and the filtrate evaporated to dryness. The resulting residue was treated with 10 mL of acetone (Honeywell), and the supernatant was removed to yield a white solid. This washing process with acetone was repeated one more time, followed by a final wash with 10 ml diethyl ether to get 19 mg (82% yield) product.

^{1}H NMR(400 MHz, D_2O): δ 5.19–5.01 (m, 7H), δ 4.51 (m, 1H), δ 4.15–3.46 (m, 42H), δ 3.25 (m, 9H). **HRMS**(ESI+): for $C_{45}H_{78}NO_{34}$; $[M]^+$, calcd. m/z 1176.4400 (100%), found 1176.4380 (100%).

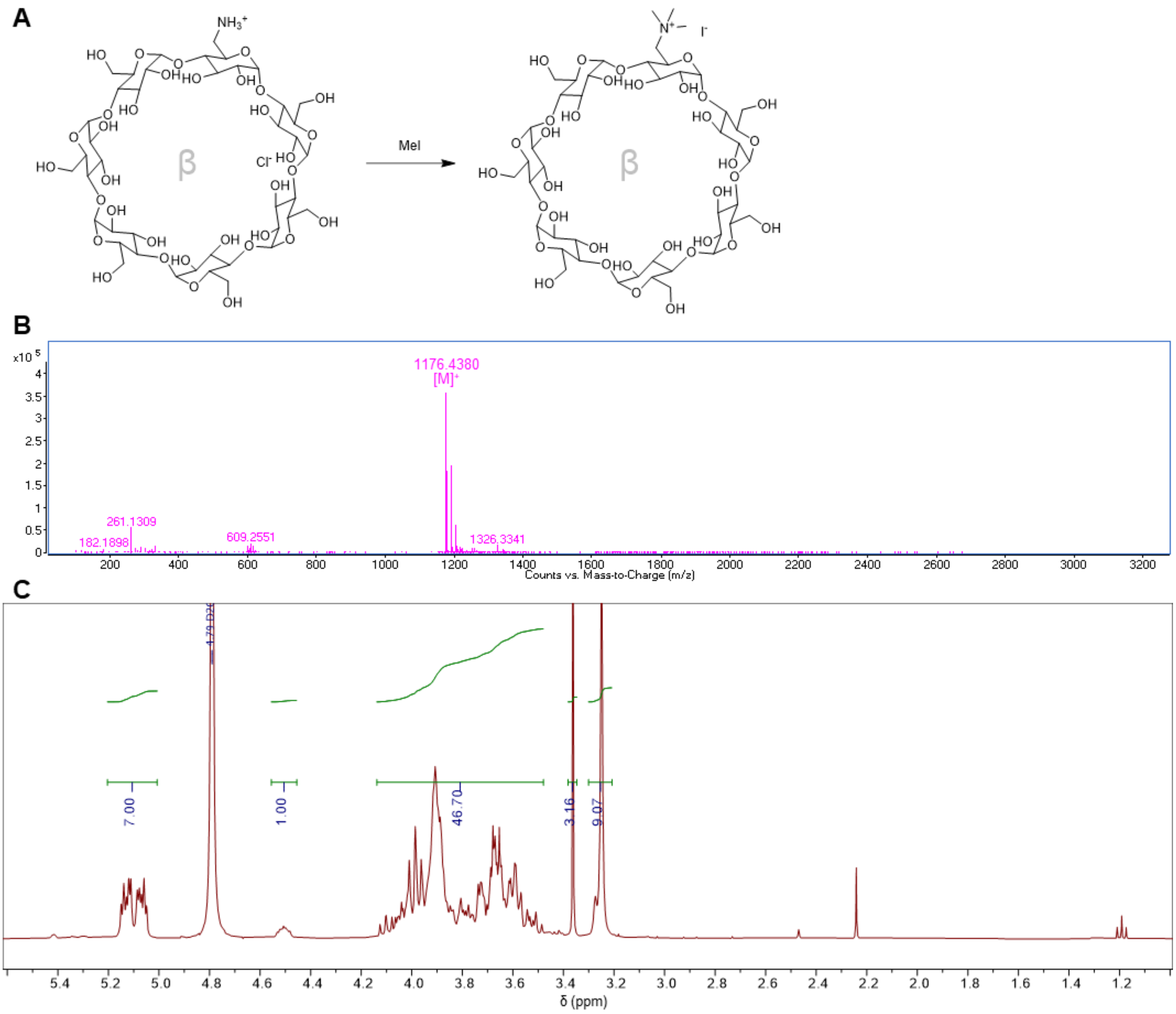

Figure S 3. **A** Synthesis of Mono-(6-TMA)-β-CD. **B** HRMS spectrum of Mono-(6-TMA)-β-CD. **C** ^{1}H NMR spectrum of Mono-(6-TMA)-β-CD.

2.1.4. Di(6-TMA)-β-CD

A 10 mL round-bottom flask equipped with a magnetic stirrer was placed under a nitrogen atmosphere and charged with 20 mg of di(6-NH_2)-β-CD·HCl (0.017 mmol). Subsequently, 2 mL of methanol, dried over 3 Å molecular sieves, was added. 0.1 ml methyl iodide and 11 mg (0.131 mmol) anhydrous sodium bicarbonate were introduced into the solution, and the mixture was refluxed for 35 hours with additional portions of methyl iodide (0.1 ml) added at 15-hour and 20-hour intervals. Following the reaction, the mixture was diluted with 10 mL of methanol, filtered, and the filtrate was evaporated to dryness. The resulting residue was treated with 10 mL of acetone, and the supernatant was removed to obtain a white solid. The washing process with acetone was repeated once, followed by a final wash with diethyl ether. The weight of the isolated solid was 15 mg (58% yield).

^{1}H NMR(400 MHz, D_2O): δ 5.12 (m, 7H), δ 4.52 (m, 2H), δ 4.17–3.46 (m, 42H), δ 3.27 (s, 18H). **HRMS**(ESI+): for $C_{48}H_{86}N_2O_{33}$; $[M]^{2+}$, calcd. m/z 609.2551 (100%), found 609.2562 (100%); $[M+I]^+$, calcd. m/z 1345.4152 (100%), found 1345.4146 (100%).

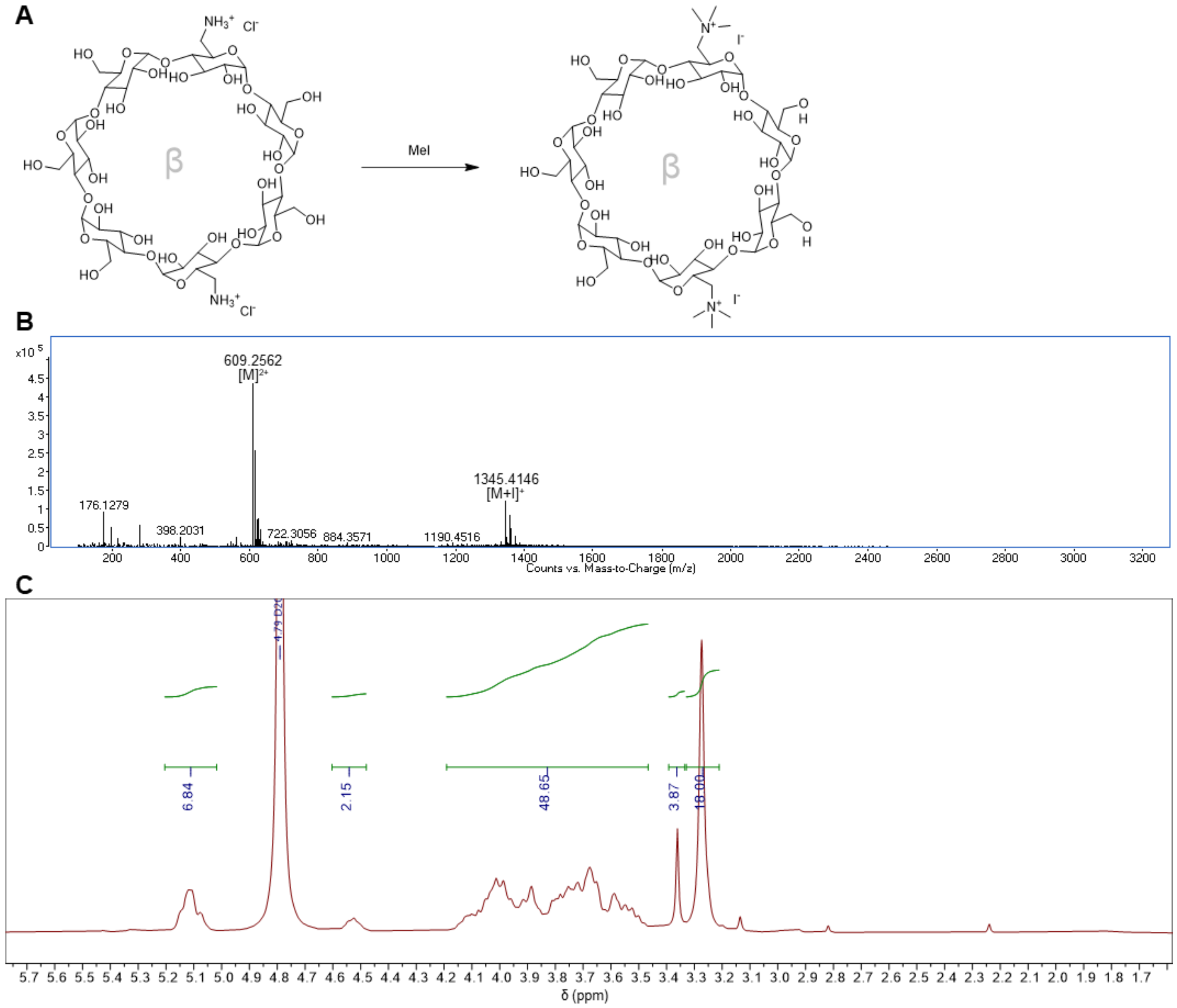

Figure S 4. **A** Synthesis of Di(6-TMA)-β-CD. **B** HRMS spectrum of Di(6-TMA)-β-CD. **C** ^{1}H NMR spectrum of Di(6-TMA)-β-CD.

3. Supplementary results and discussion

3.1. NMR Detected Titrations

3.1.1. With α-CD as a host

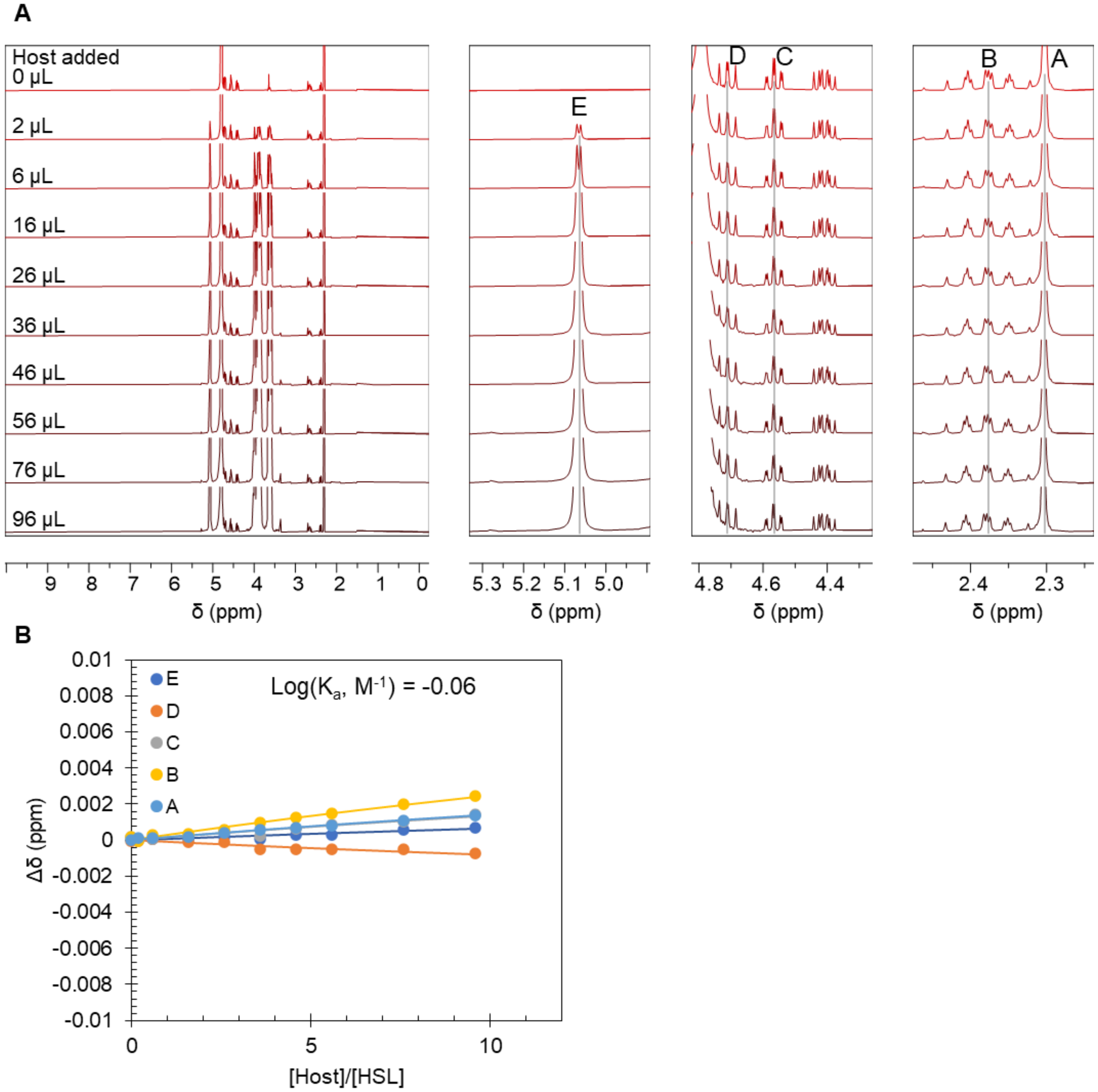

Figure S 5. **A** ^{1}H NMR titration of oxo-C4-HSL + α-CD. [Host]=60 mM, $[HSL]_{initial}$=1 mM, $V_{initial}$ = 600 μL. **B** Traces of Δδ from titration data per selected peak. Curves correspond to 1:1 model (Log(Ka) indicated) at each experimental [Host]/[HSL] value.

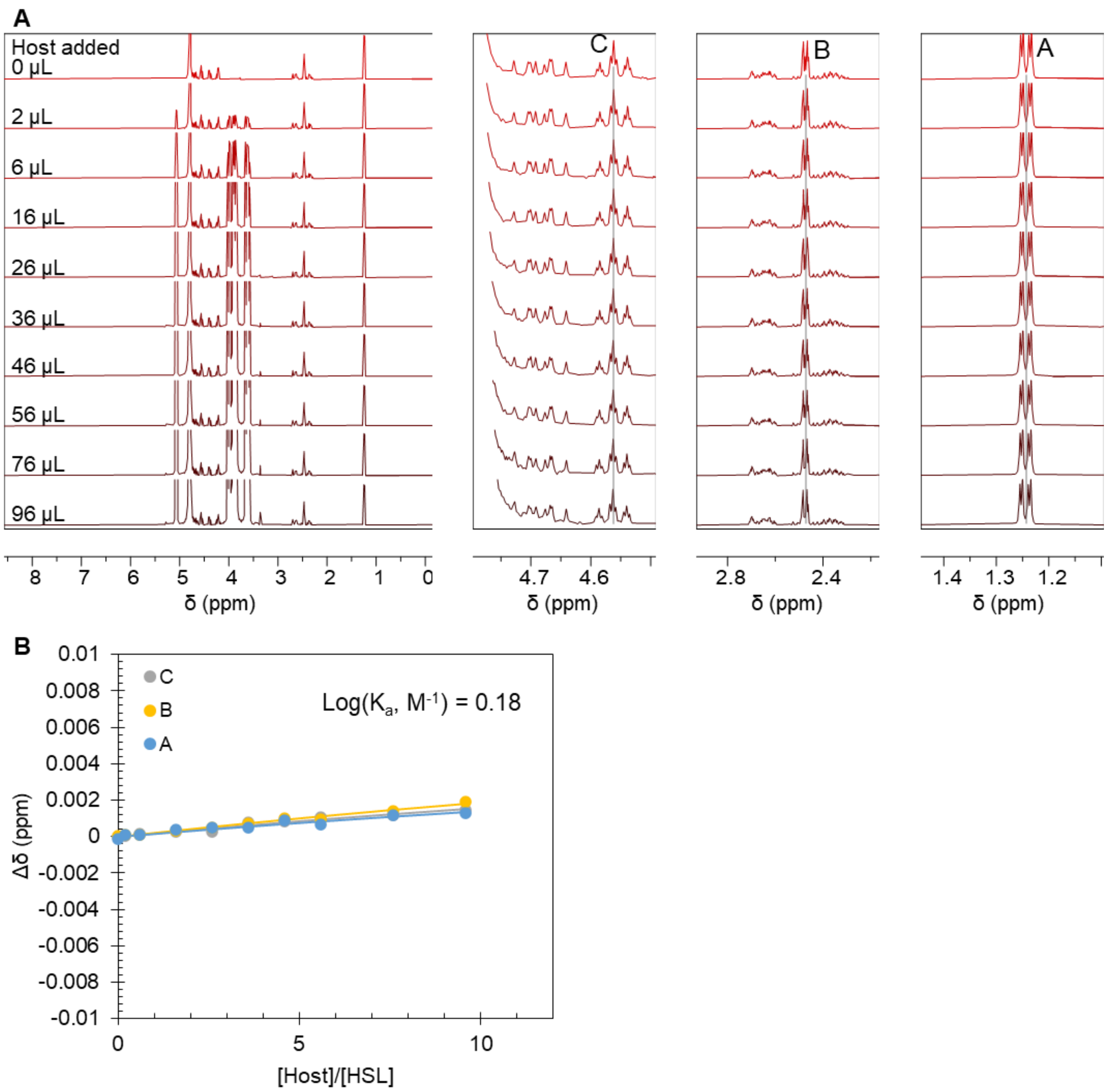

Figure S 6. **A** ^{1}H NMR titration of OH-C4-HSL + α-CD. [Host]=60 mM, $[HSL]_{initial}$=1 mM, $V_{initial}$ = 600 μL. **B** Traces of Δδ from titration data per selected peak. Curves correspond to 1:1 model (Log(Ka) indicated) at each experimental [Host]/[HSL] value.

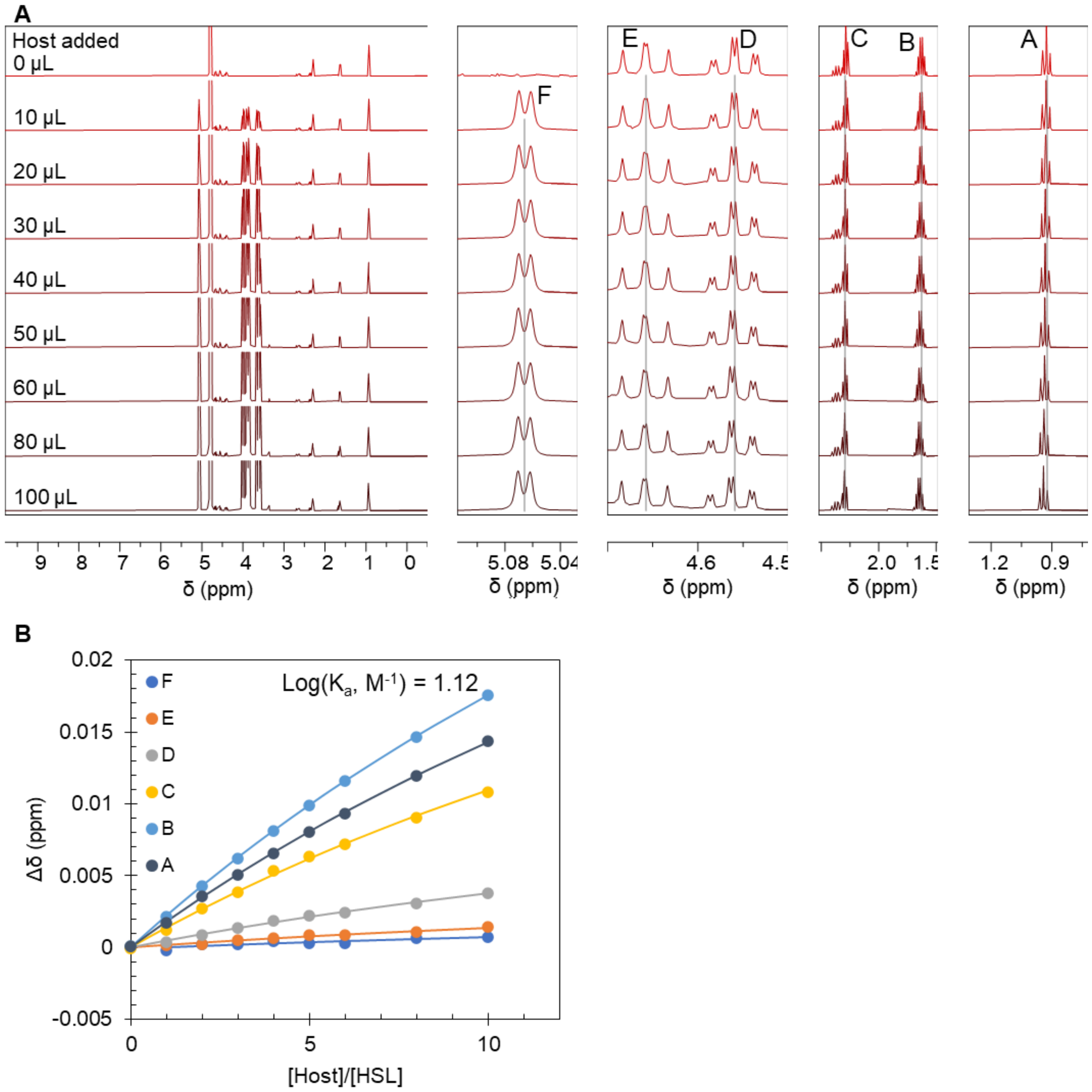

Figure S 7. **A** ^{1}H NMR titration of H-C4-HSL + α-CD. [Host]=60 mM, $[HSL]_{initial}$=1 mM, $V_{initial}$ = 600 µL. **B** Traces of Δδ from titration data per selected peak. Curves correspond to 1:1 model (Log(Ka) indicated) at each experimental [Host]/[HSL] value.

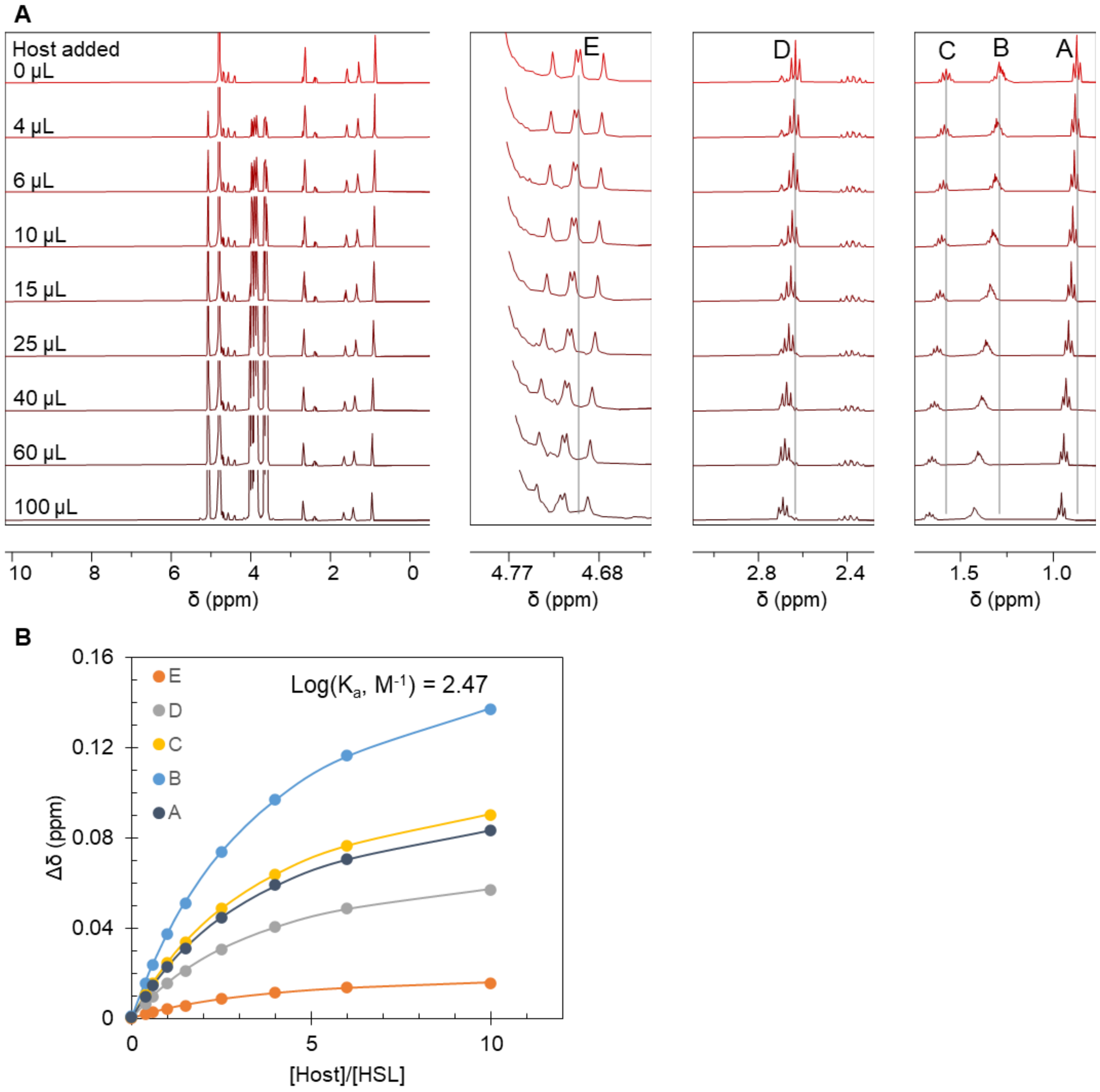

Figure S 8. **A** ^{1}H NMR titration of oxo-C8-HSL + α-CD. [Host]=60 mM, $[HSL]_{initial}$=1 mM, $V_{initial}$ = 600 µL. **B** Traces of Δδ from titration data per selected peak. Curves correspond to 1:1 model (Log(Ka) indicated) at each experimental [Host]/[HSL] value.

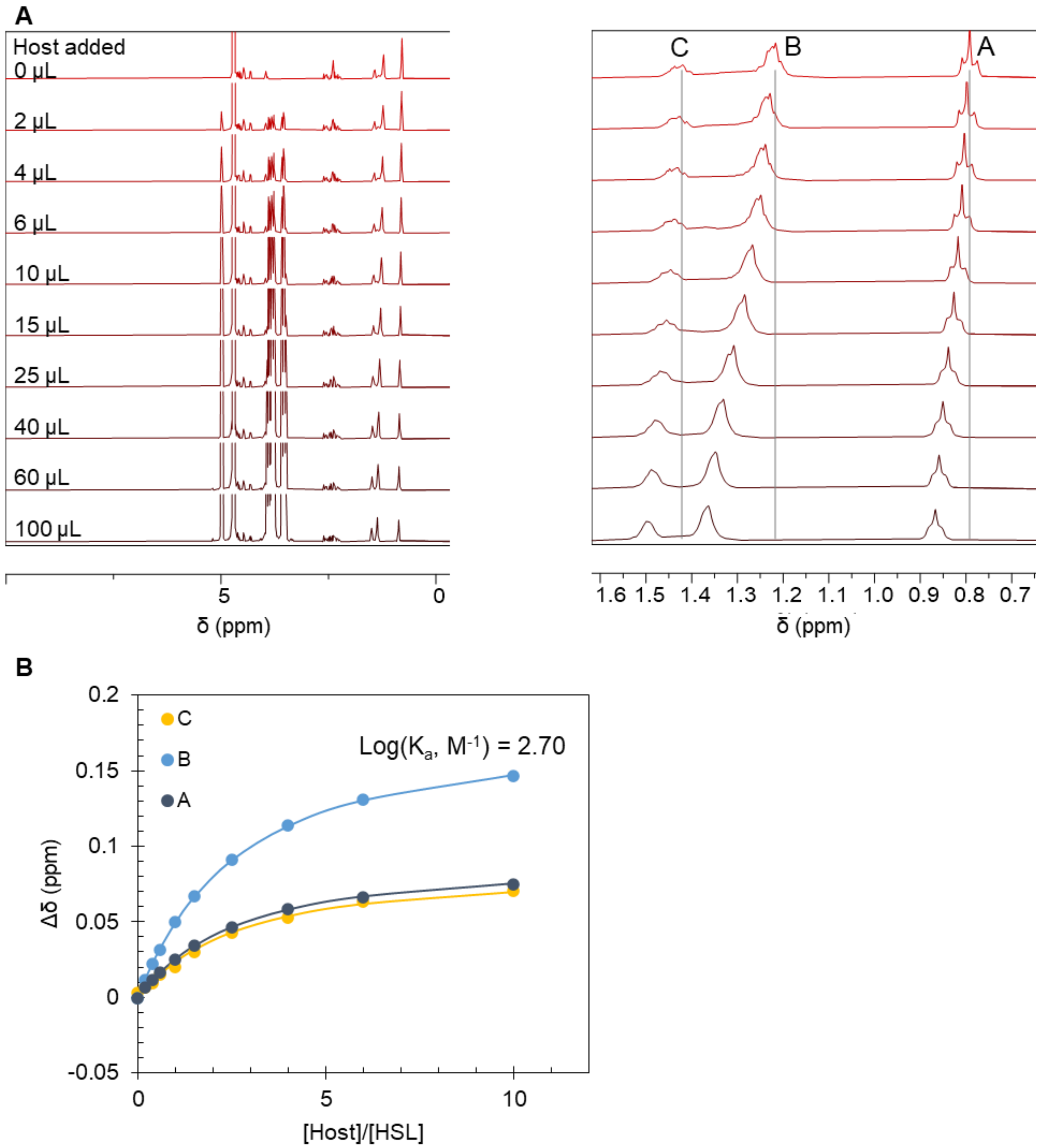

Figure S 9. **A** ^{1}H NMR titration of OH-C8-HSL + α-CD. [Host]=60 mM, $[HSL]_{initial}$=1 mM, $V_{initial}$ = 600 µL. **B** Traces of Δδ from titration data per selected peak. Curves correspond to 1:1 model (Log(Ka) indicated) at each experimental [Host]/[HSL] value.

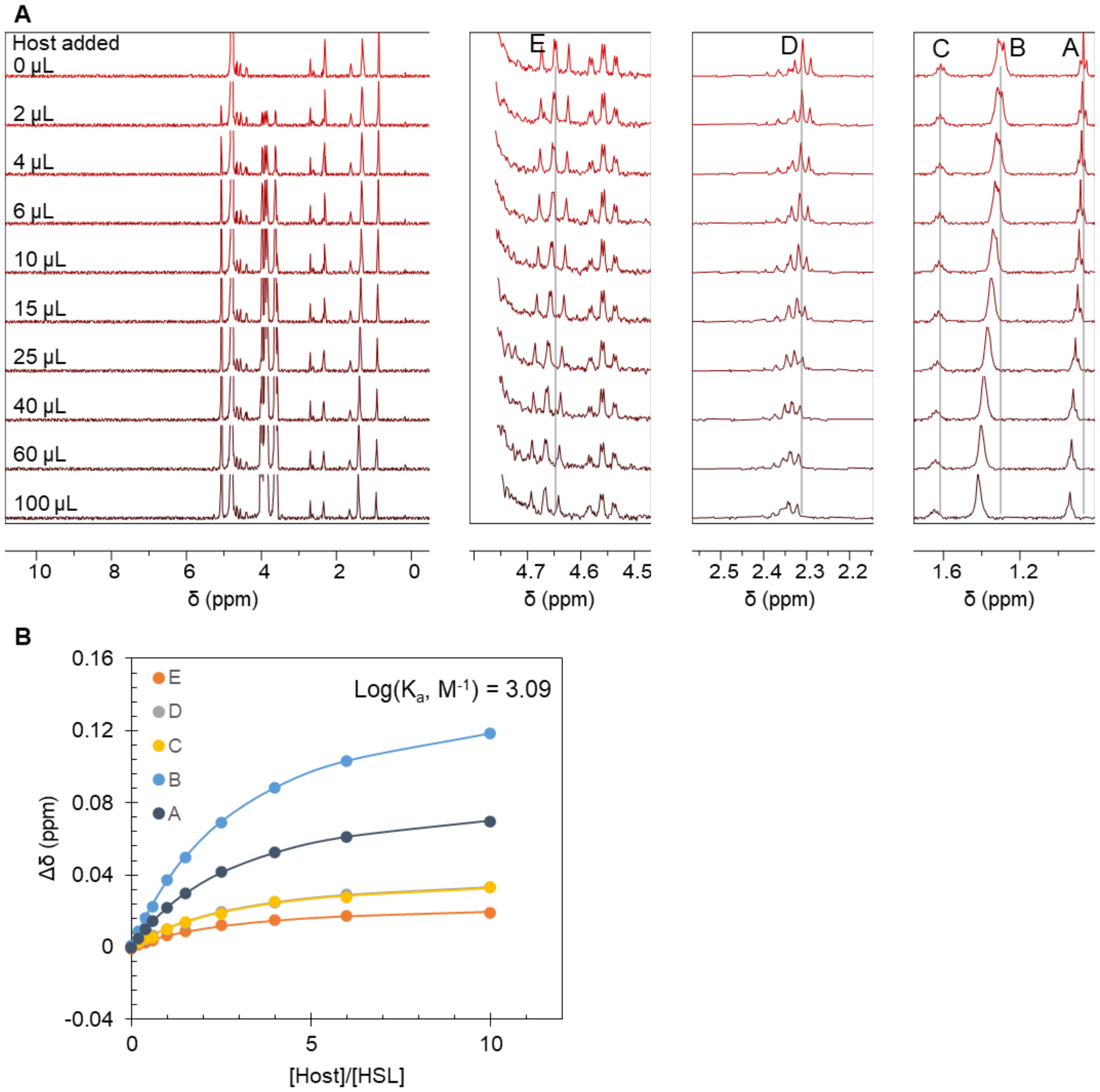

Figure S 10. **A** ^{1}H NMR titration of H-C8-HSL + α-CD. [Host]=20 mM, $[HSL]_{initial}$=0.33 mM, $V_{initial}$ = 600 µL. **B** Traces of Δδ from titration data per selected peak. Curves correspond to 1:1 model (Log(Ka) indicated) at each experimental [Host]/[HSL] value.

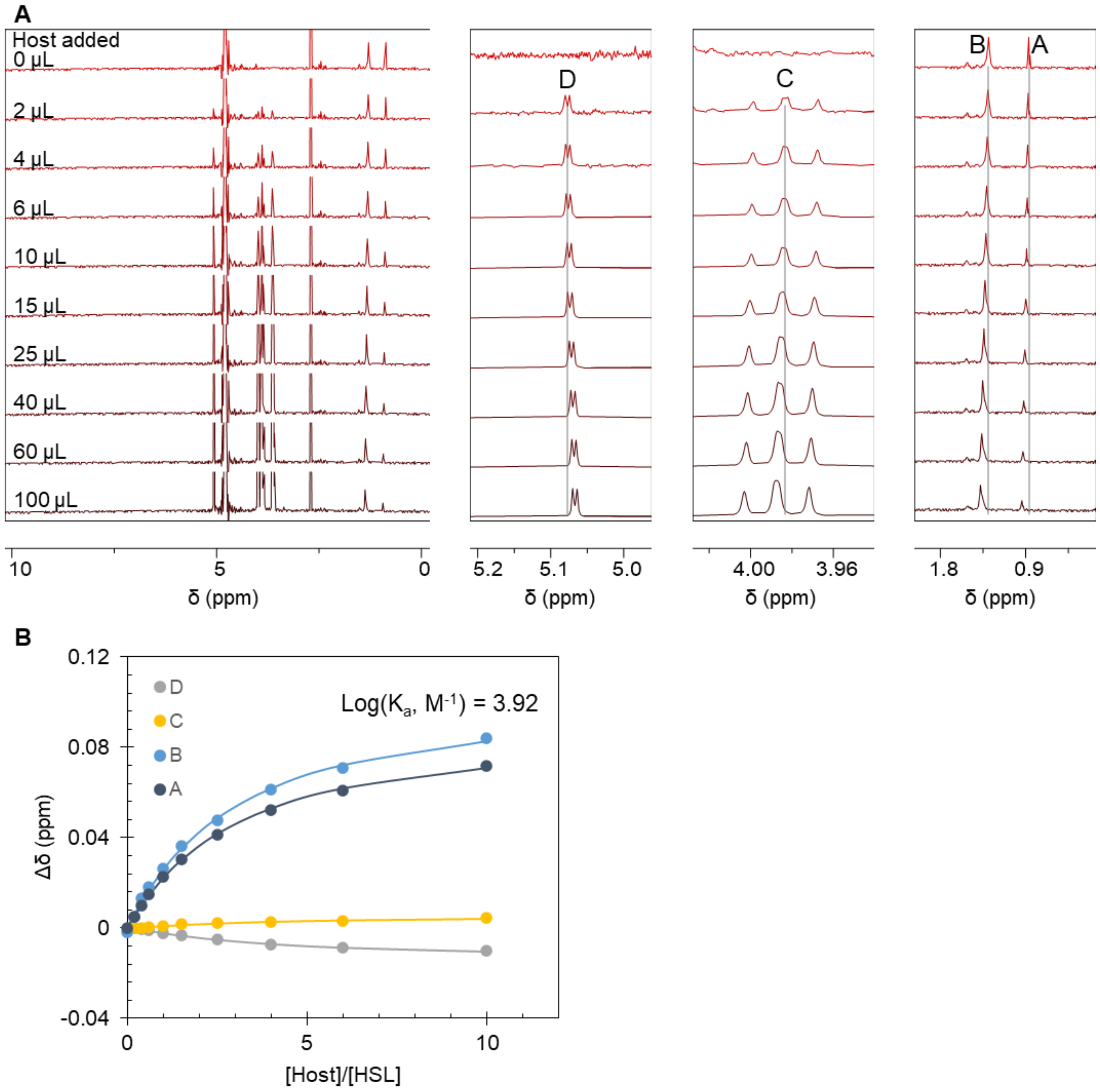

Figure S 11. **A** ^{1}H NMR titration of OH-C12-HSL + α-CD. [Host]=3 mM, $[HSL]_{initial}$=0.05 mM, $V_{initial}$ = 600 µL. **B** Traces of Δδ from titration data per selected peak. Curves correspond to 1:1 model (Log(Ka) indicated) at each experimental [Host]/[HSL] value.

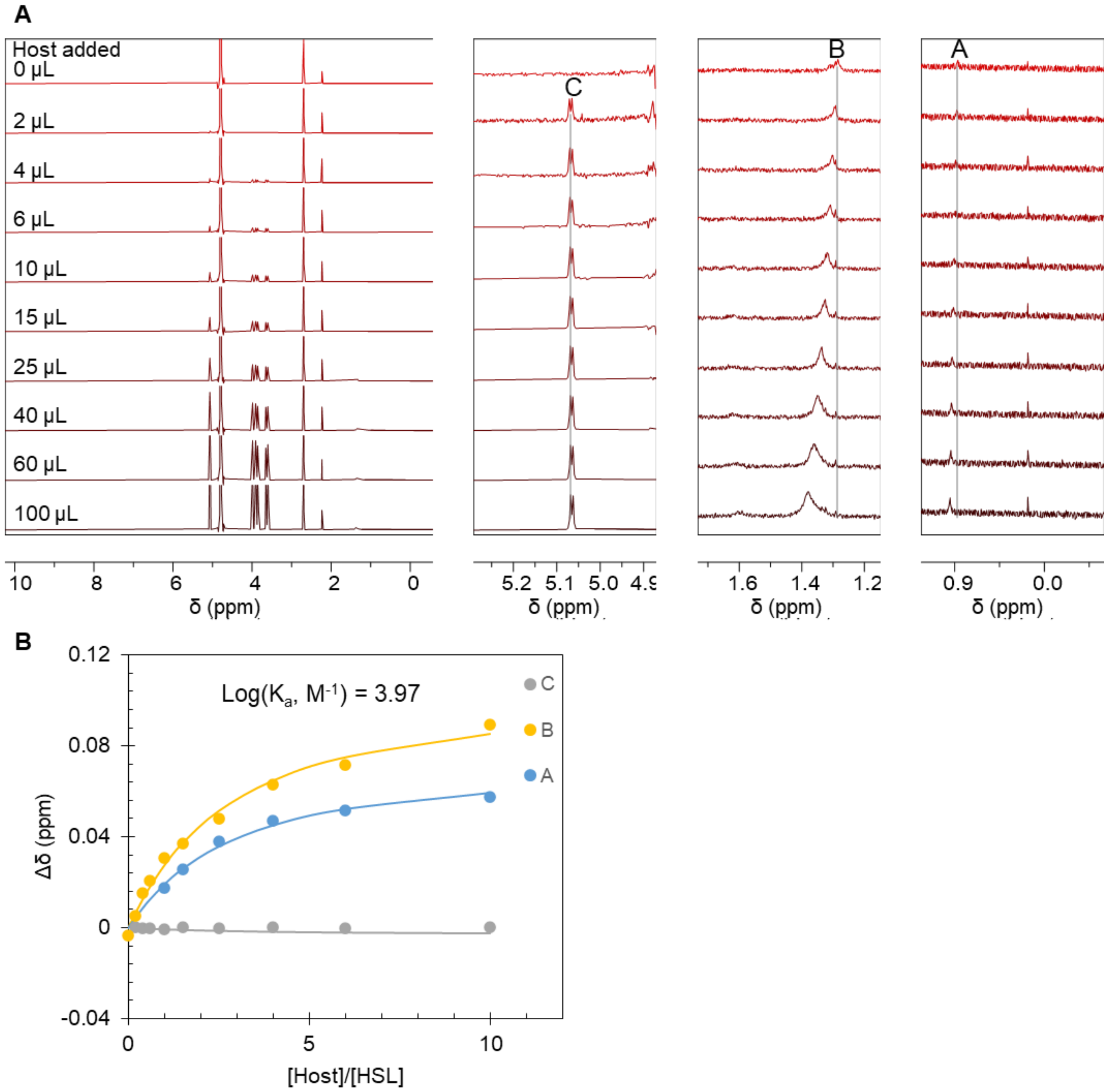

Figure S 12. **A** ^{1}H NMR titration of H-C12-HSL + α-CD. [Host]=3 mM, $[HSL]_{initial}$=0.05 mM, $V_{initial}$ = 600 µL. **B** Traces of Δδ from titration data per selected peak. Curves correspond to 1:1 model (Log(Ka) indicated) at each experimental [Host]/[HSL] value.

3.1.2. With β-CD as a host

In light of other titrations with C4-HSLs, the titration of oxo-C4-HSL against β-CD was performed with fewer concentration points. Based on the very minute recorded peak shifts (1/10 000s of ppm), the binding affinity was determined to be "very low" (in the context of this work).

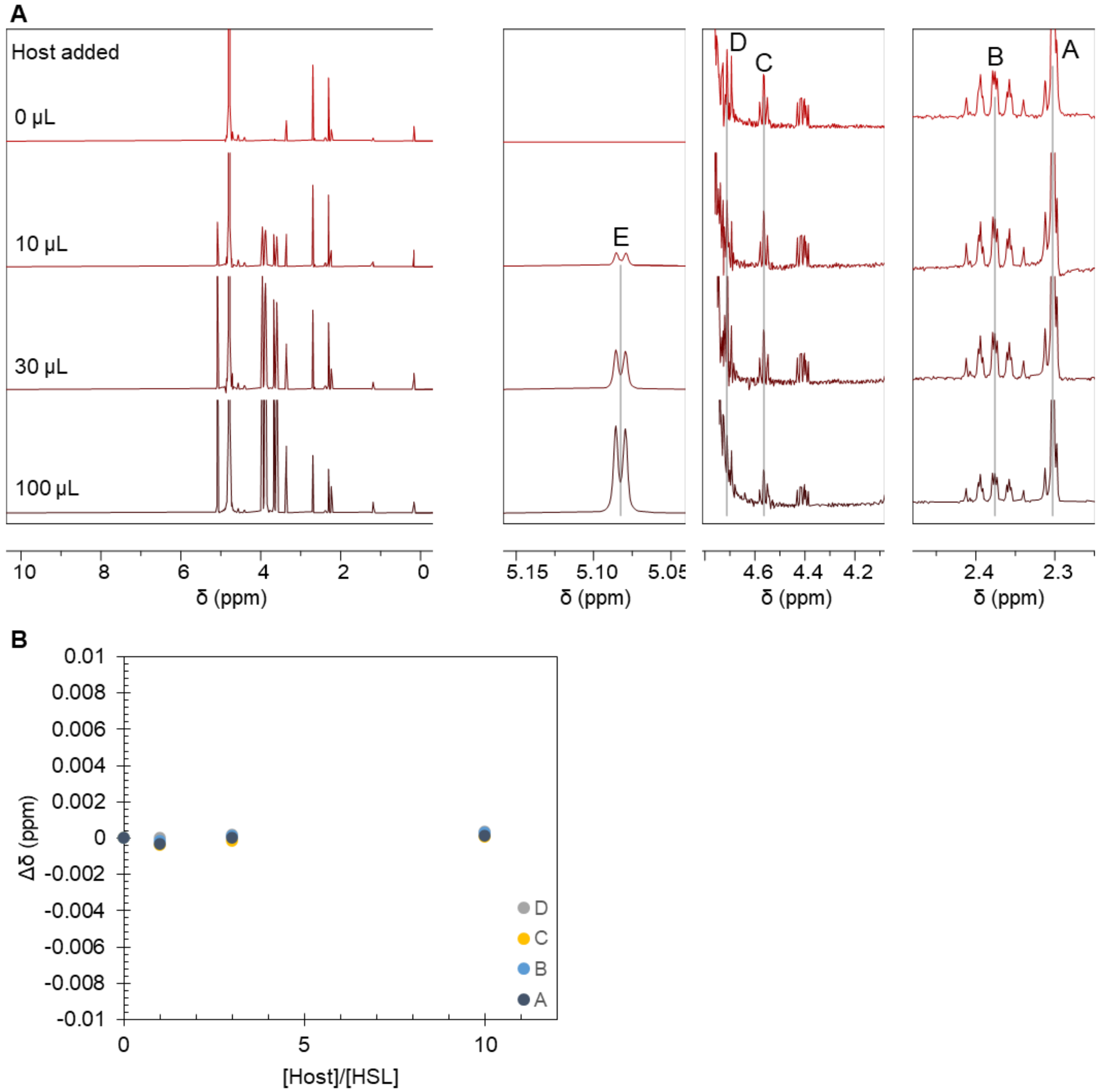

Figure S 13. **A** ^{1}H NMR titration of oxo-C4-HSL + β-CD. [Host]=9 mM, $[HSL]_{initial}$=0.15 mM, $V_{initial}$ = 600 µL. **B** Traces of Δδ from titration data per selected peak.

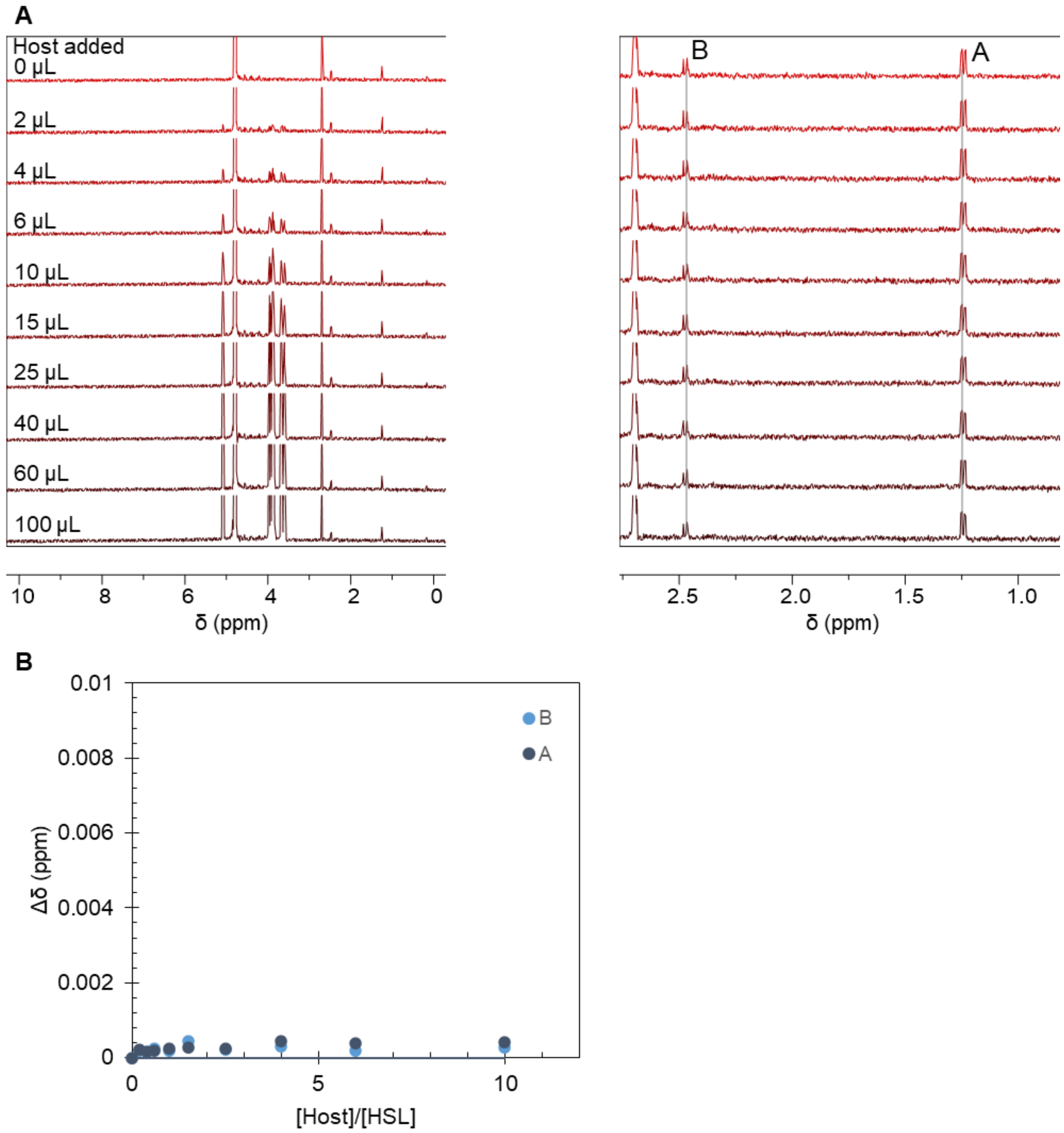

Figure S 14. **A** ^{1}H NMR titration of OH-C4-HSL + β-CD. [Host]=9 mM, $[HSL]_{initial}$=0.15 mM, $V_{initial}$ = 600 μL. **B** Traces of Δδ from titration data per selected peak.

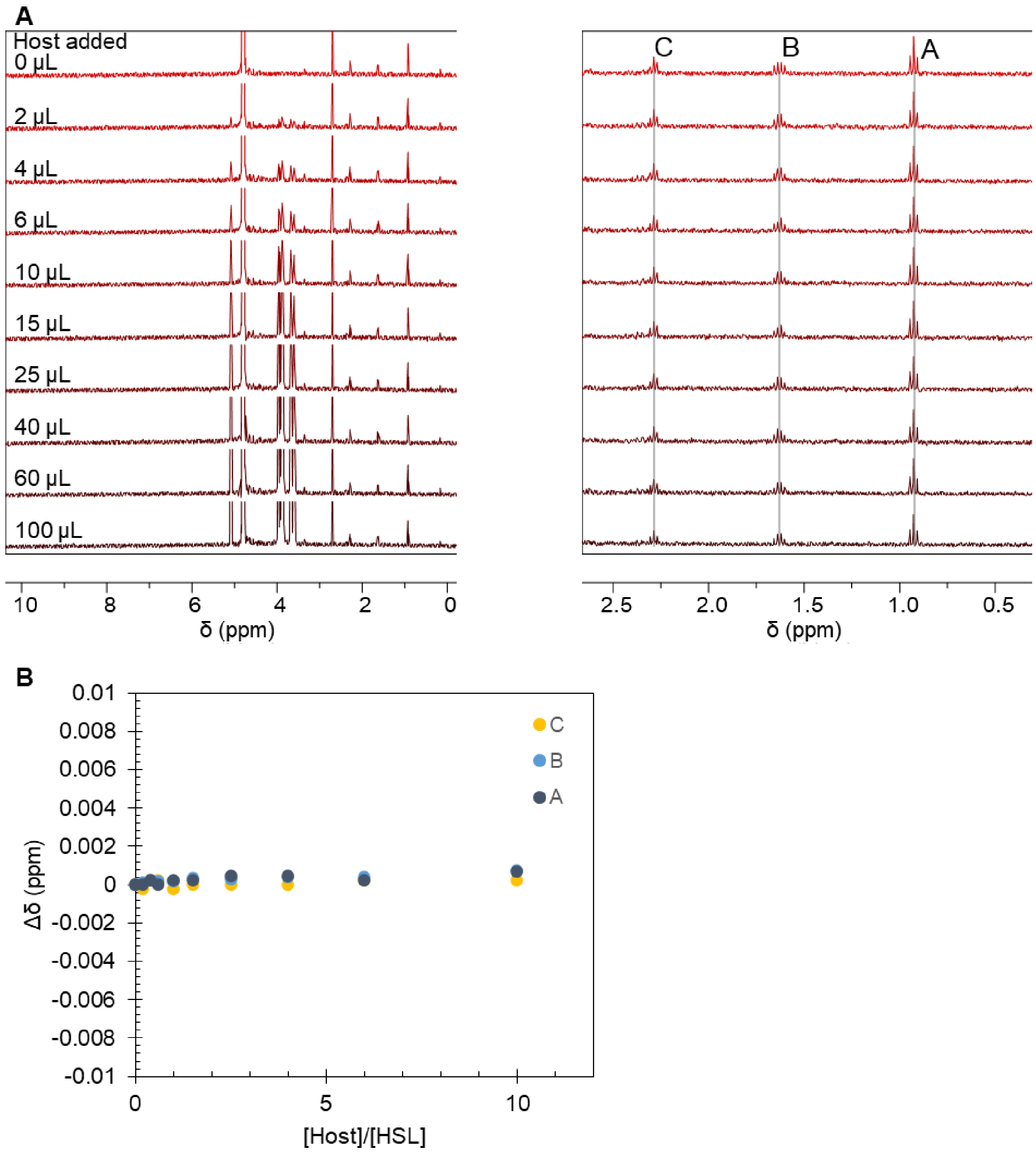

Figure S 15. **A** ^{1}H NMR titration of H-C4-HSL + β-CD. [Host]=9 mM, $[HSL]_{initial}$=0.15 mM, $V_{initial}$ = 600 µL. **B** Traces of Δδ from titration data per selected peak.

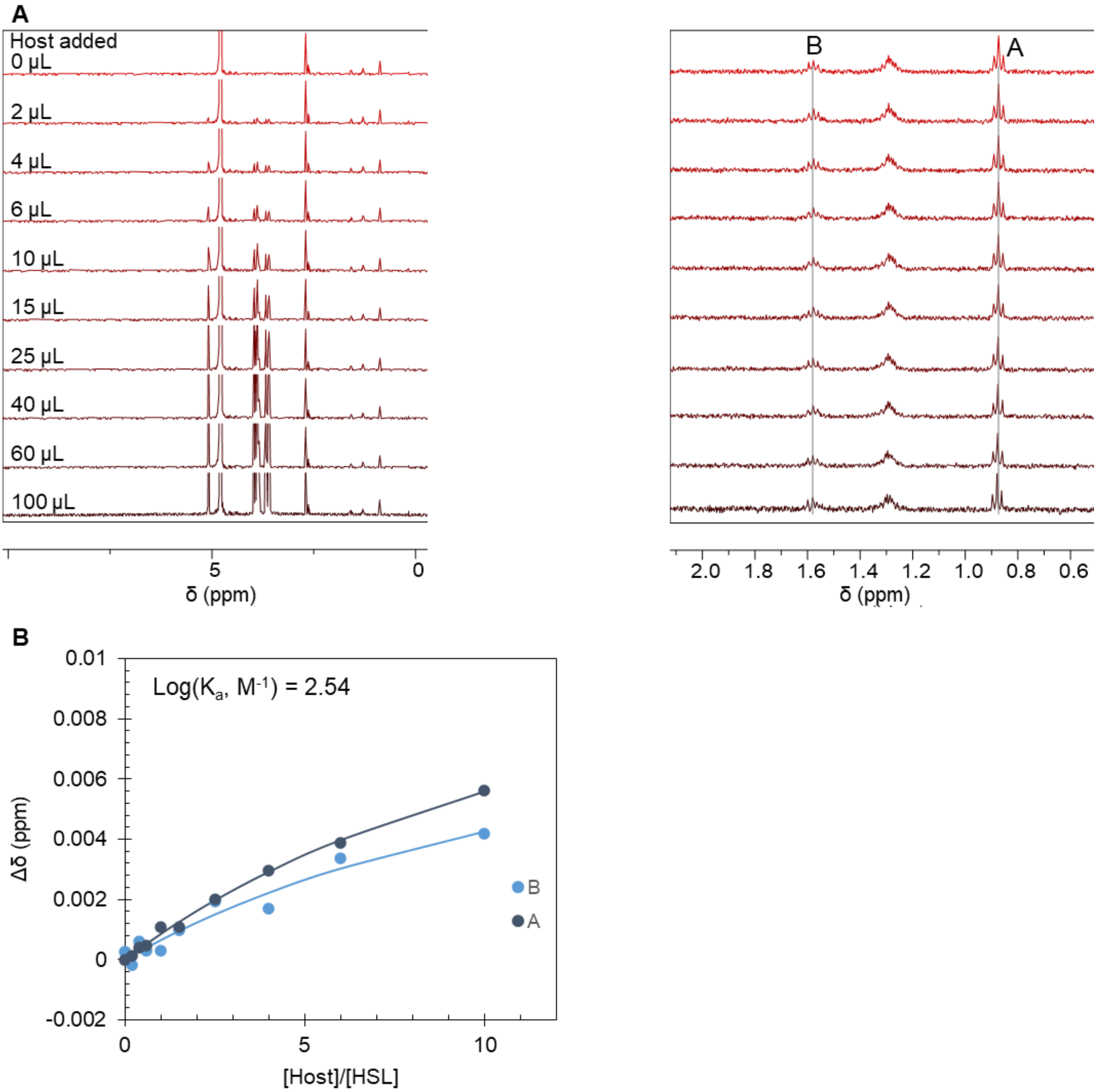

Figure S 16. **A** ^{1}H NMR titration of oxo-C8-HSL + β-CD. [Host]=9 mM, $[HSL]_{initial}$=0.15 mM, $V_{initial}$ = 600 µL. **B** Traces of Δδ from titration data per selected peak. Curves correspond to 1:1 model (Log(Ka) indicated) at each experimental [Host]/[HSL] value.

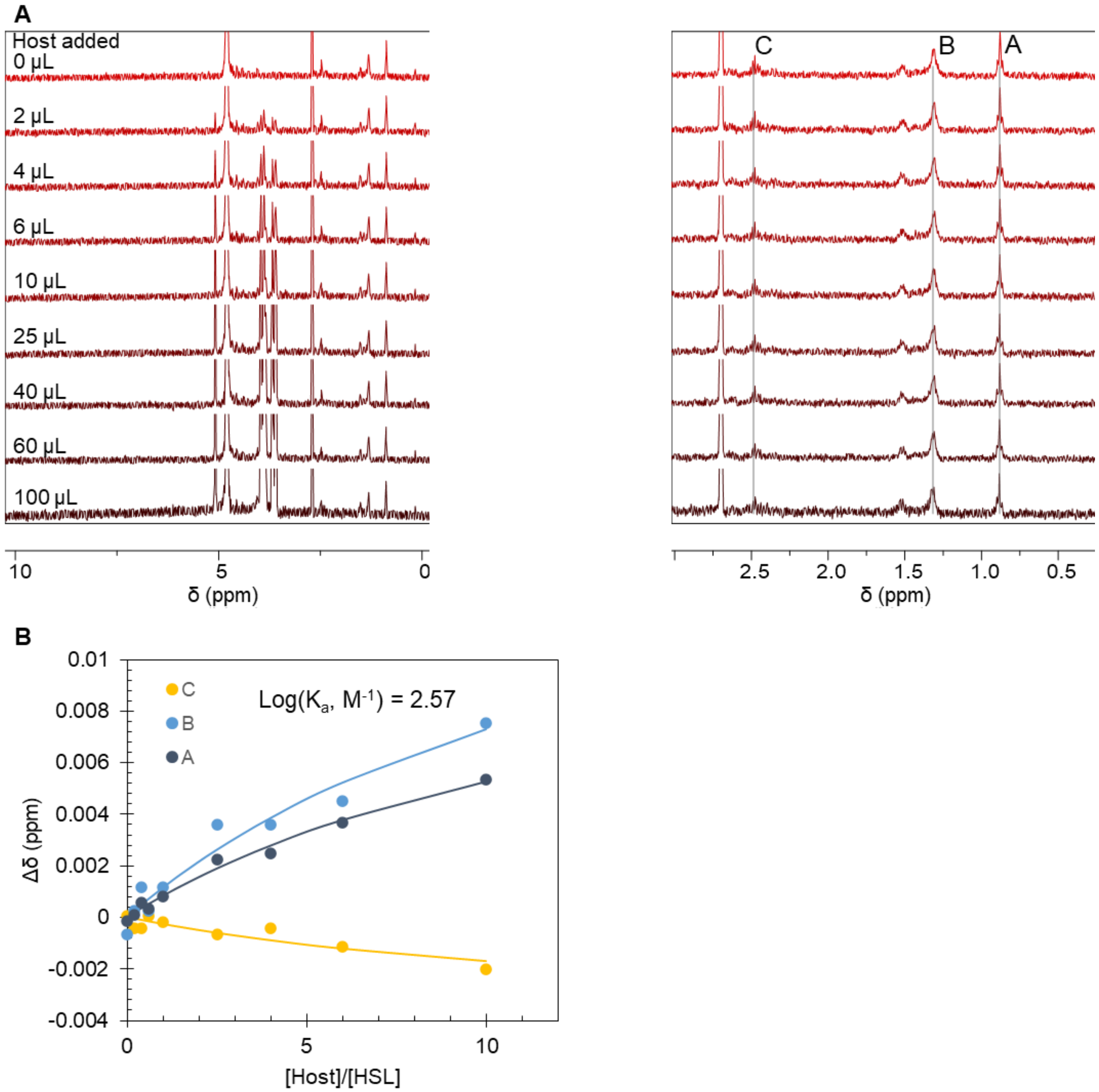

Figure S 17. **A** ^{1}H NMR titration of OH-C8-HSL + β-CD. [Host]=9 mM, $[HSL]_{initial}$=0.15 mM, $V_{initial}$ = 600 µL. **B** Traces of Δδ from titration data per selected peak. Curves correspond to 1:1 model (Log(Ka) indicated) at each experimental [Host]/[HSL] value.

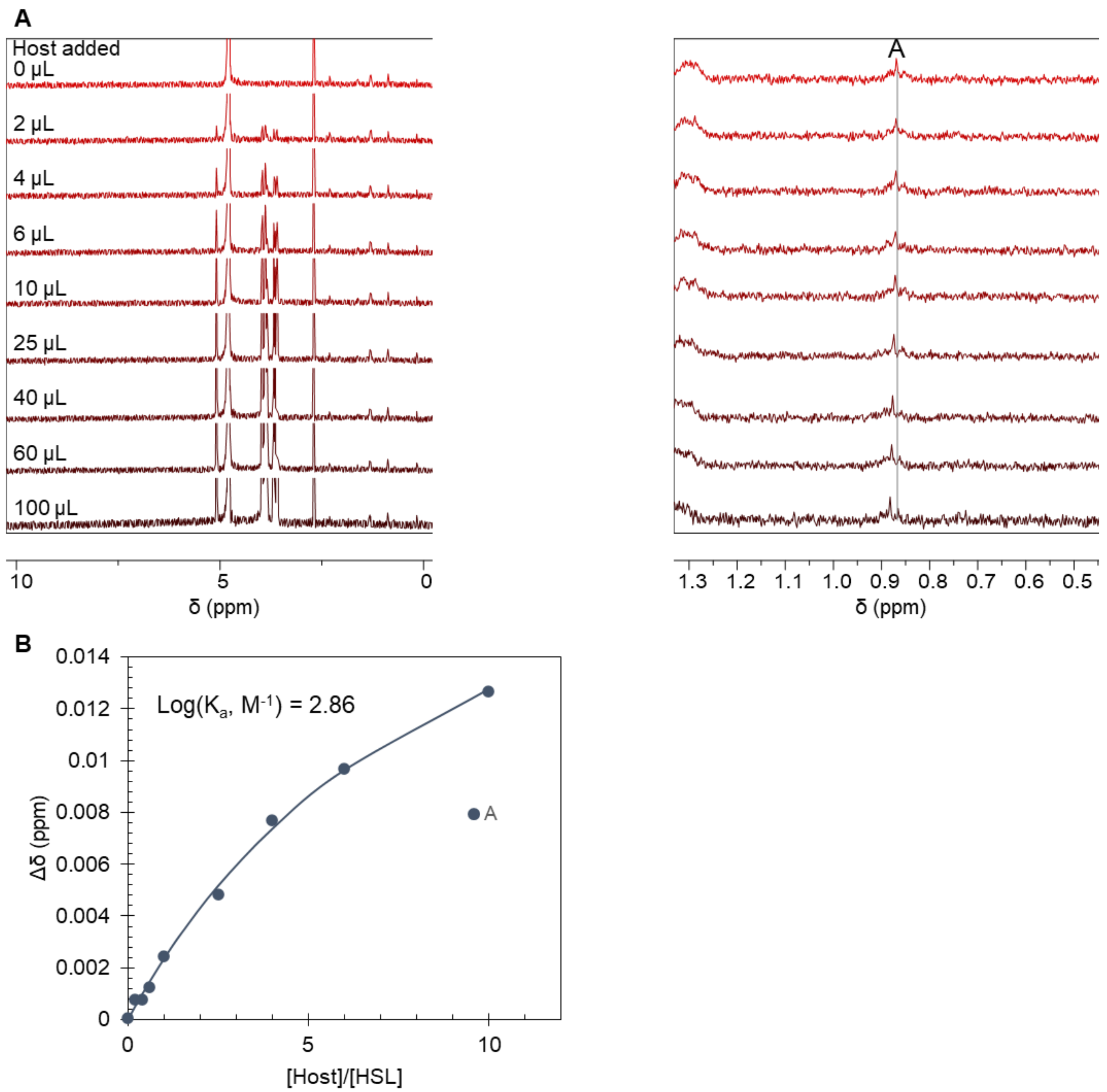

Figure S 18. **A** ^{1}H NMR titration of H-C8-HSL + β-CD. [Host]=9 mM, $[HSL]_{initial}$=0.15 mM, $V_{initial}$ = 600 µL. **B** Traces of Δδ from titration data per selected peak. Curves correspond to 1:1 model (Log(Ka) indicated) at each experimental [Host]/[HSL] value.

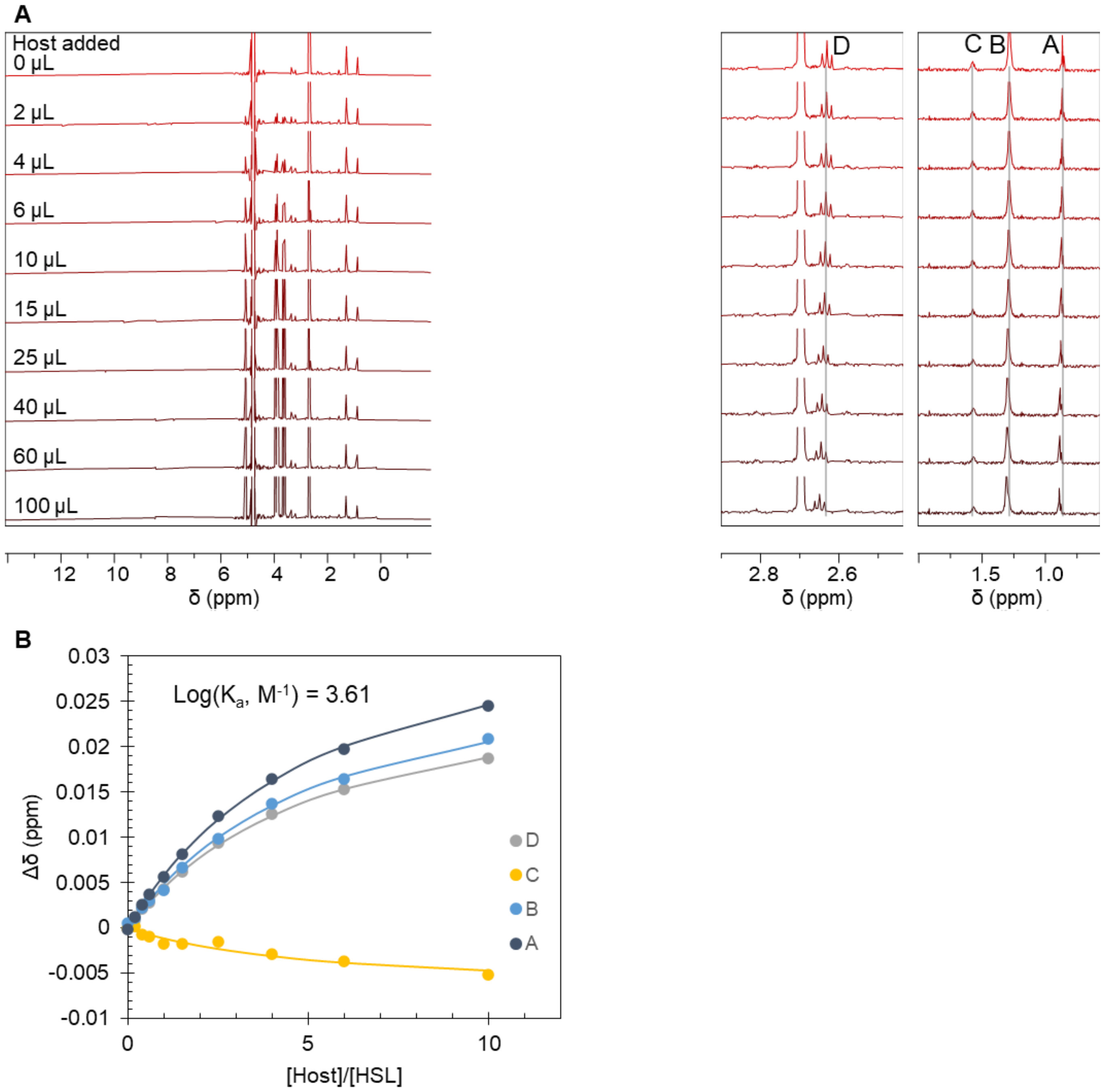

Figure S 19. **A** ^{1}H NMR titration of oxo-C12-HSL + β-CD. [Host]=3 mM, $[HSL]_{initial}$=0.05 mM, $V_{initial}$ = 600 µL. **B** Traces of Δδ from titration data per selected peak. Curves correspond to 1:1 model (Log(Ka) indicated) at each experimental [Host]/[HSL] value.

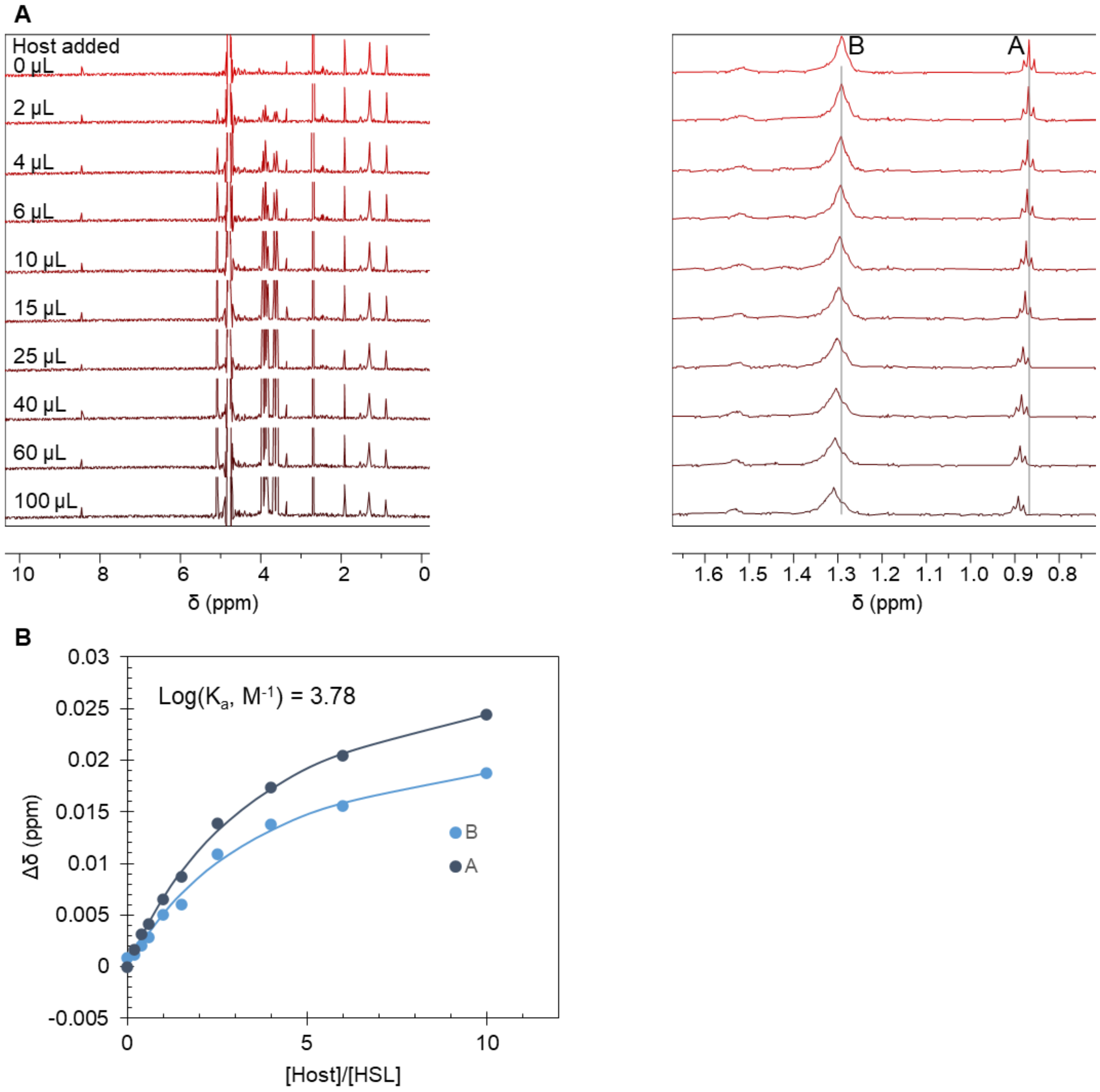

Figure S 20. **A** ^{1}H NMR titration of OH-C12-HSL + β-CD. [Host]=3 mM, $[HSL]_{initial}$=0.05 mM, $V_{initial}$ = 600 µL. **B** Traces of Δδ from titration data per selected peak. Curves correspond to 1:1 model (Log(Ka) indicated) at each experimental [Host]/[HSL] value.

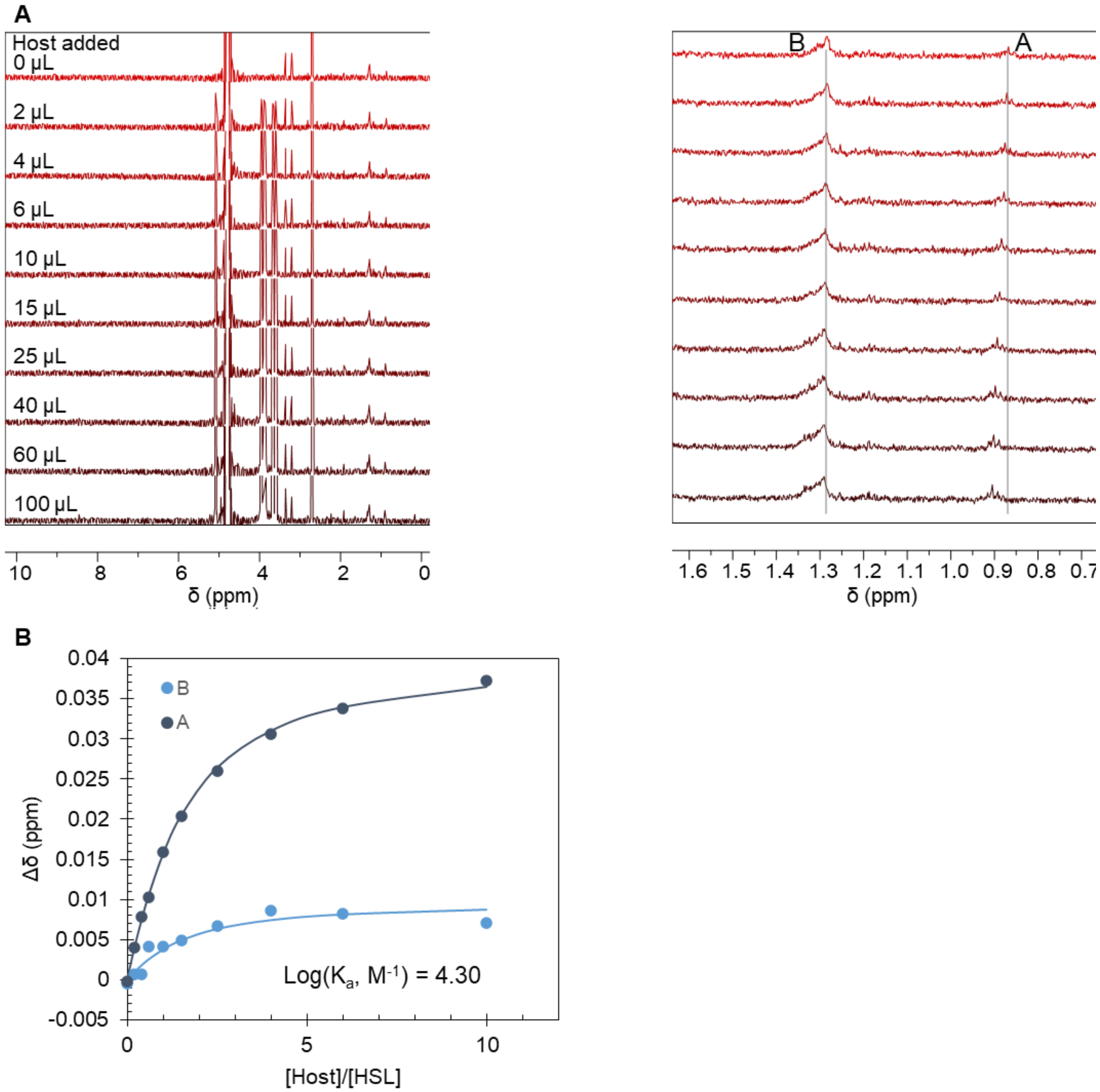

Figure S 21. **A** ^{1}H NMR titration of H-C12-HSL + β-CD. [Host]=3 mM, $[HSL]_{initial}$=0.05 mM, $V_{initial}$ = 600 µL. **B** Traces of Δδ from titration data per selected peak. Curves correspond to 1:1 model (Log(Ka) indicated) at each experimental [Host]/[HSL] value.

3.1.3. With aminated α-CDs as hosts

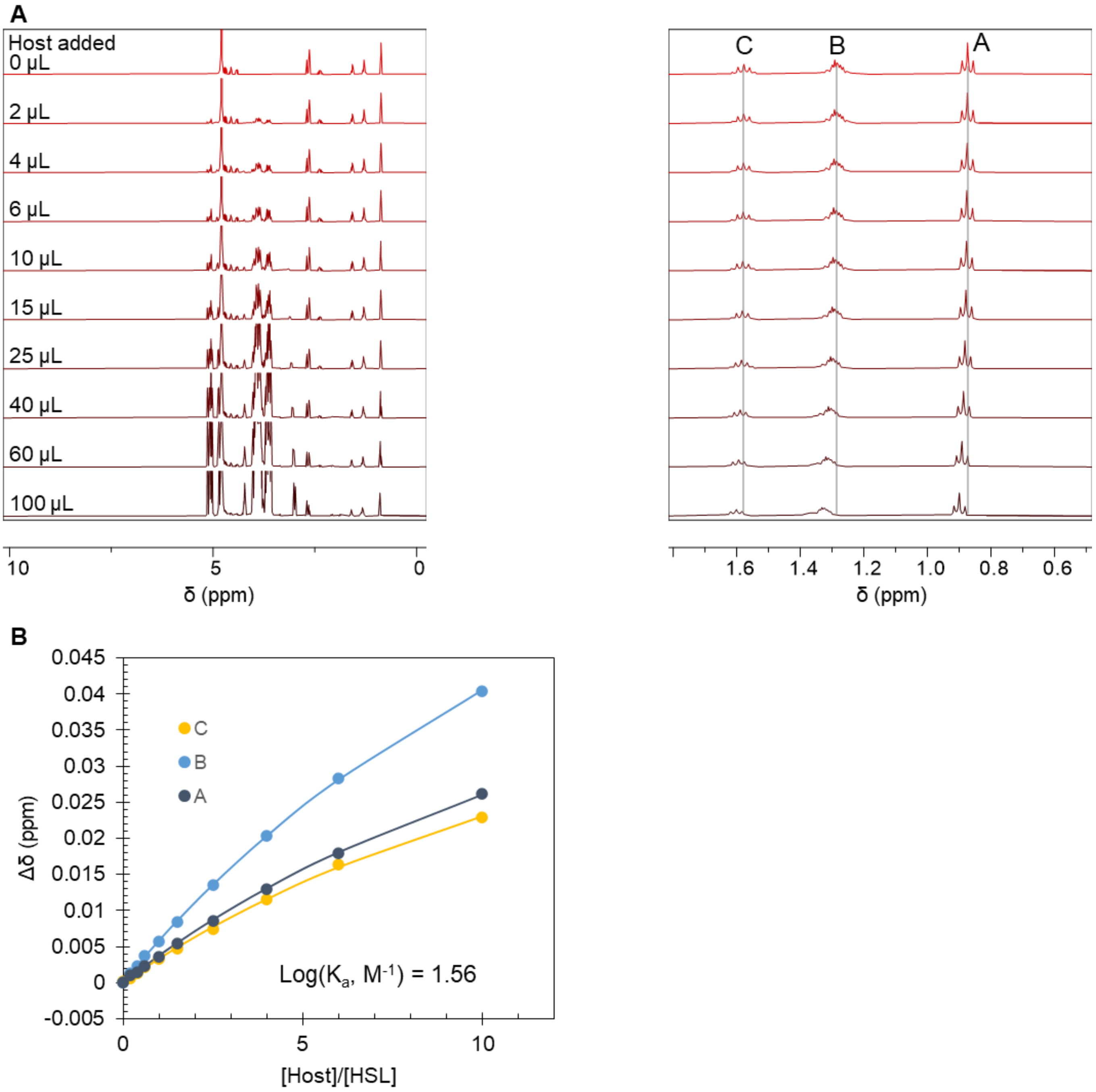

Figure S 22. **A** ^{1}H NMR titration of oxo-C8-HSL + Mono(3-NH2)-α-CDs. [Host]= 60 mM, $[HSL]_{initial}$= 1 mM, $V_{initial}$ = 600 μL. **B** Traces of Δδ from titration data per selected peak. Curves correspond to 1:1 model (Log(Ka) indicated) at each experimental [Host]/[HSL] value.

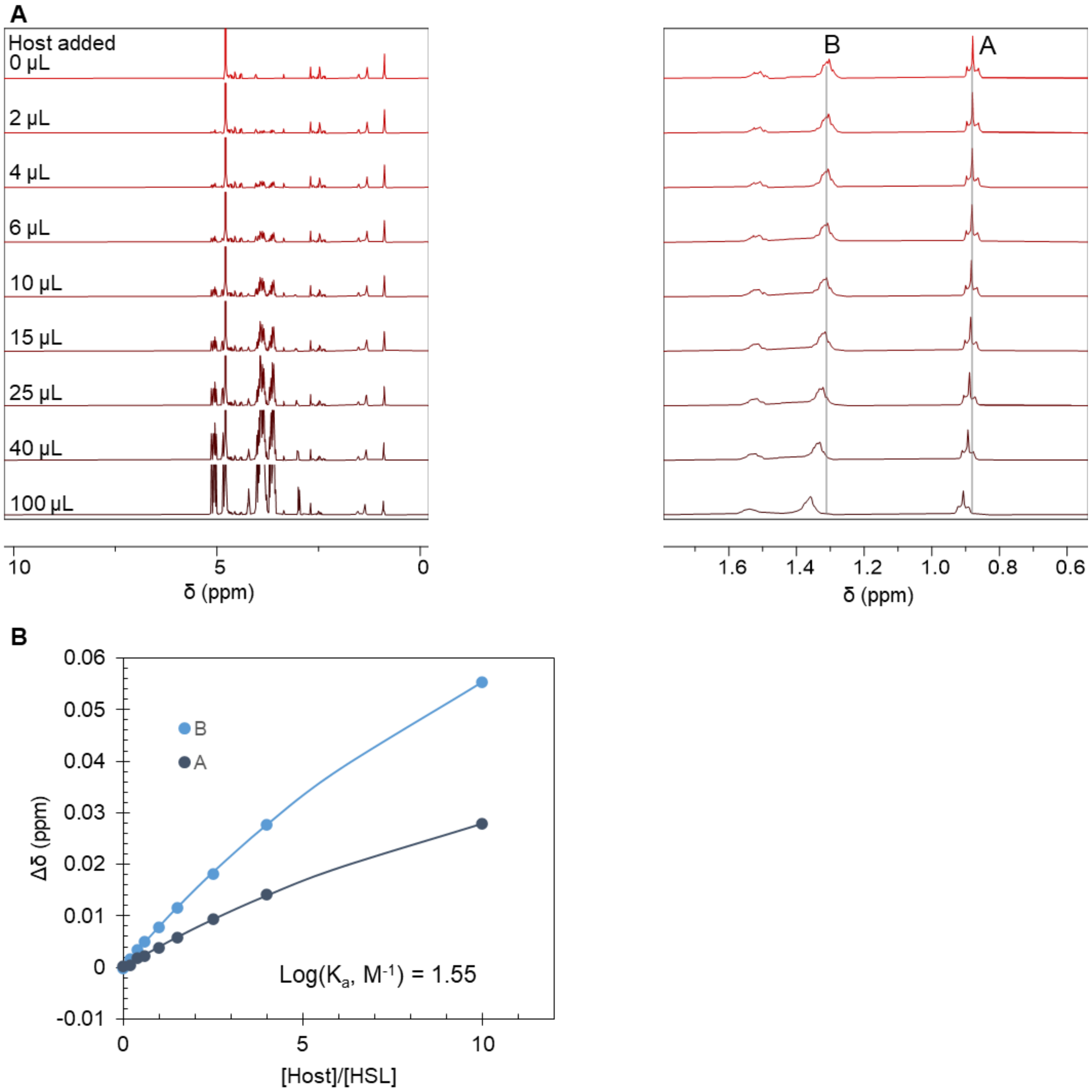

Figure S 23. **A** ^{1}H NMR titration of OH-C8-HSL + Mono(3-NH_2)-α-CDs. [Host]= 60 mM, $[HSL]_{initial}$= 1 mM, $V_{initial}$ = 600 μL. **B** Traces of Δδ from titration data per selected peak. Curves correspond to 1:1 model (Log(Ka) indicated) at each experimental [Host]/[HSL] value.

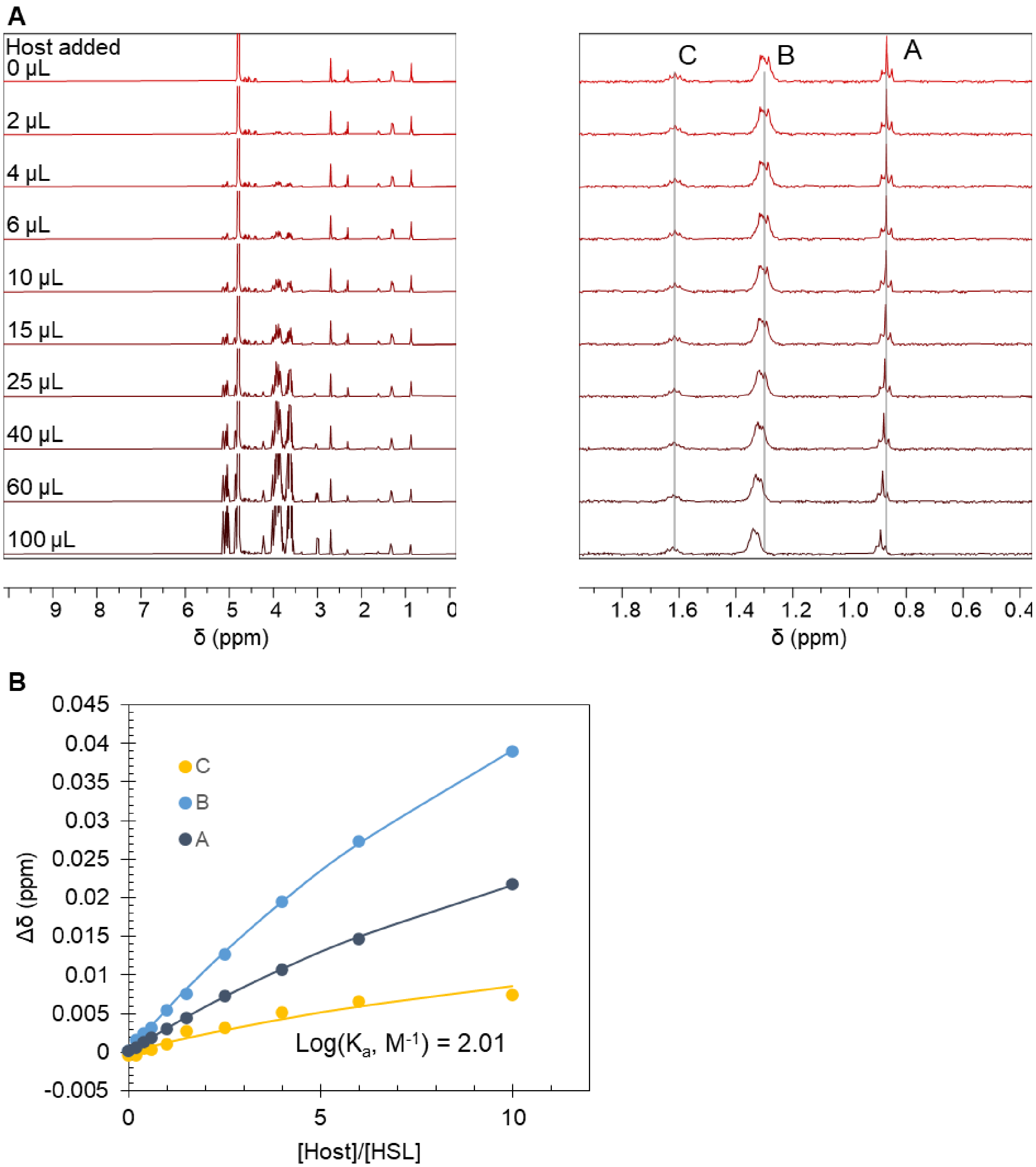

Figure S 24. **A** ^{1}H NMR titration of H-C8-HSL + Mono(3-NH$_2$)-α-CDs. [Host]= 20 mM, $[HSL]_{initial}$= 0.33 mM, $V_{initial}$ = 600 µL. **B** Traces of Δδ from titration data per selected peak. Curves correspond to 1:1 model (Log(Ka) indicated) at each experimental [Host]/[HSL] value.

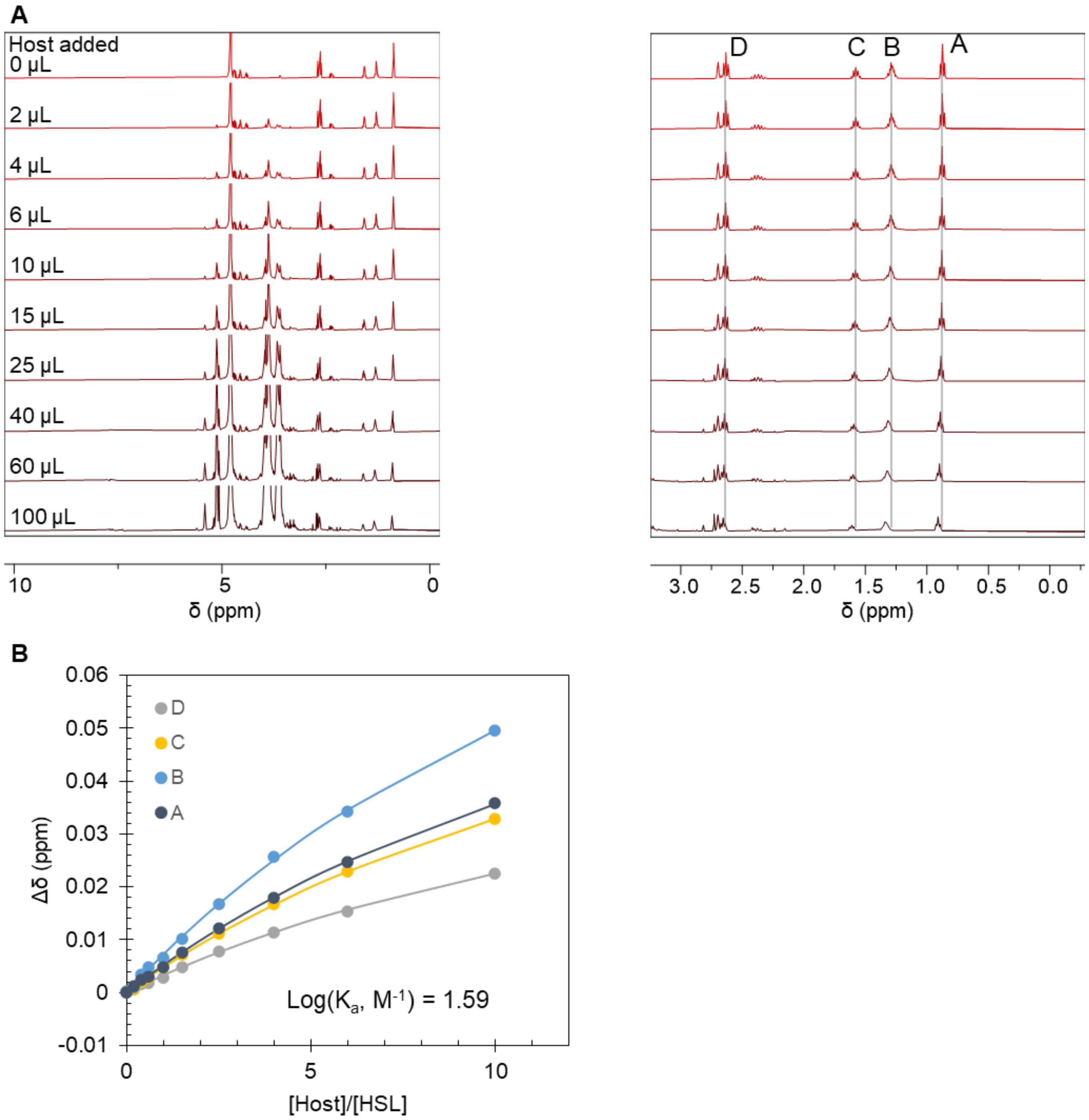

Figure S 25. **A** ^{1}H NMR titration of oxo-C8-HSL + Mono(6-NH2)-α-CDs. [Host]= 60 mM, $[HSL]_{initial}$= 1 mM, $V_{initial}$ = 600 µL. **B** Traces of Δδ from titration data per selected peak. Curves correspond to 1:1 model (Log(Ka) indicated) at each experimental [Host]/[HSL] value.

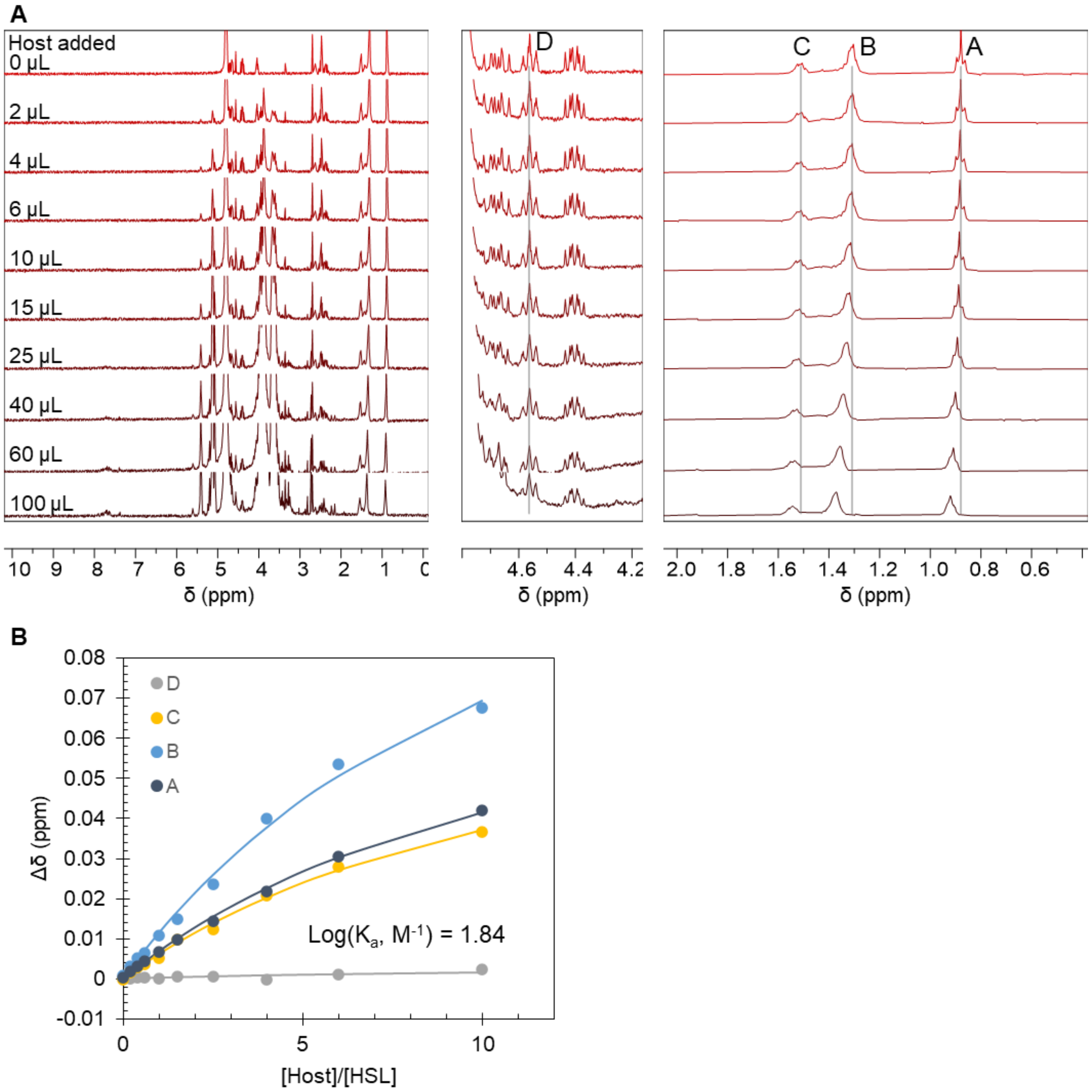

Figure S 26. **A** ^{1}H NMR titration of OH-C8-HSL + Mono(6-NH_2)-α-CDs. [Host]= 60 mM, $[HSL]_{initial}$= 1 mM, $V_{initial}$ = 600 µL. **B** Traces of Δδ from titration data per selected peak. Curves correspond to 1:1 model (Log(Ka) indicated) at each experimental [Host]/[HSL] value.

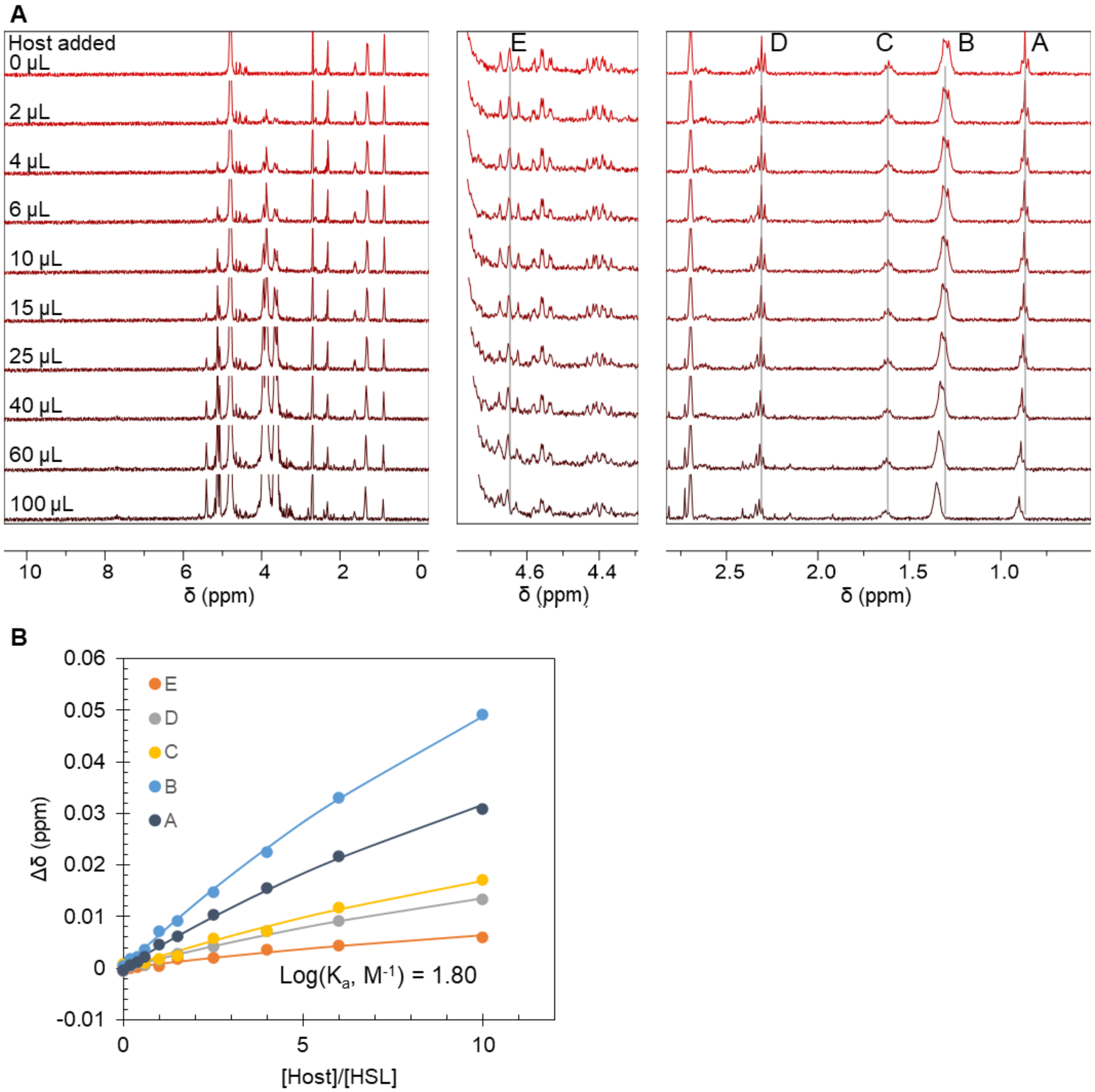

Figure S 27. **A** ^{1}H NMR titration of H-C8-HSL + Mono(6-NH$_2$)-α-CDs. [Host]= 20 mM, $[HSL]_{initial}$= 0.33 mM, $V_{initial}$ = 600 µL. **B** Traces of Δδ from titration data per selected peak. Curves correspond to 1:1 model (Log(Ka) indicated) at each experimental [Host]/[HSL] value.

3.2. Isothermal Titration Calorimetry

The settled-on parameters for ITC were a reference power of 10 µcal/s and 3 µL injections. Initial HSL concentration of 0.05 mM in cell, and CD concentration of 2.475 mM in syringe only led to detectable signals for C12-HSLs. For C8-HSLs, higher concentration runs with 0.2 mM HSL in cell and 9.9 mM CD in syringe were carried out, which yielded detectable signals. The overall concentration increase likely led to considerable adsorption of H-C8-HSL onto the ITC cell surfaces, as evidenced by a steady decrease from the pre-run nominal baseline (within 1 µcal/s of the reference power of 10 µcal/s) to ca. 8.3 µcal/s upon switching on stirring. The baseline then partially recovered throughout the titration, corresponding with a partial solubilization of the HSL with α-CD. HSLs are known to show aggregation behavior past critical concentrations[6], so adsorption onto the cell's steel surface at high enough concentrations is not unreasonable. This adsorption likely explains the considerably lower ITC-derived binding constant for H-C8-HSL, compared to the NMR-derived value (log(K_a, M^{-1}) 2.2 versus 3.1).

Among the C4-HSLs, the titration of α-CD against H-C4-HSL seemingly yielded a binding signal; however, the curving part of the integral series only consists of 4 points in the titration, the thermogram signals of which only mildly differ from the further points. The points can be fitted to a 1:1 binding model, yielding an abnormally high binding constant (log(Ka) ca. 4.6). Based on the abnormal binding constant and few points constituting a binding-like curve, this data was deemed an outlier.

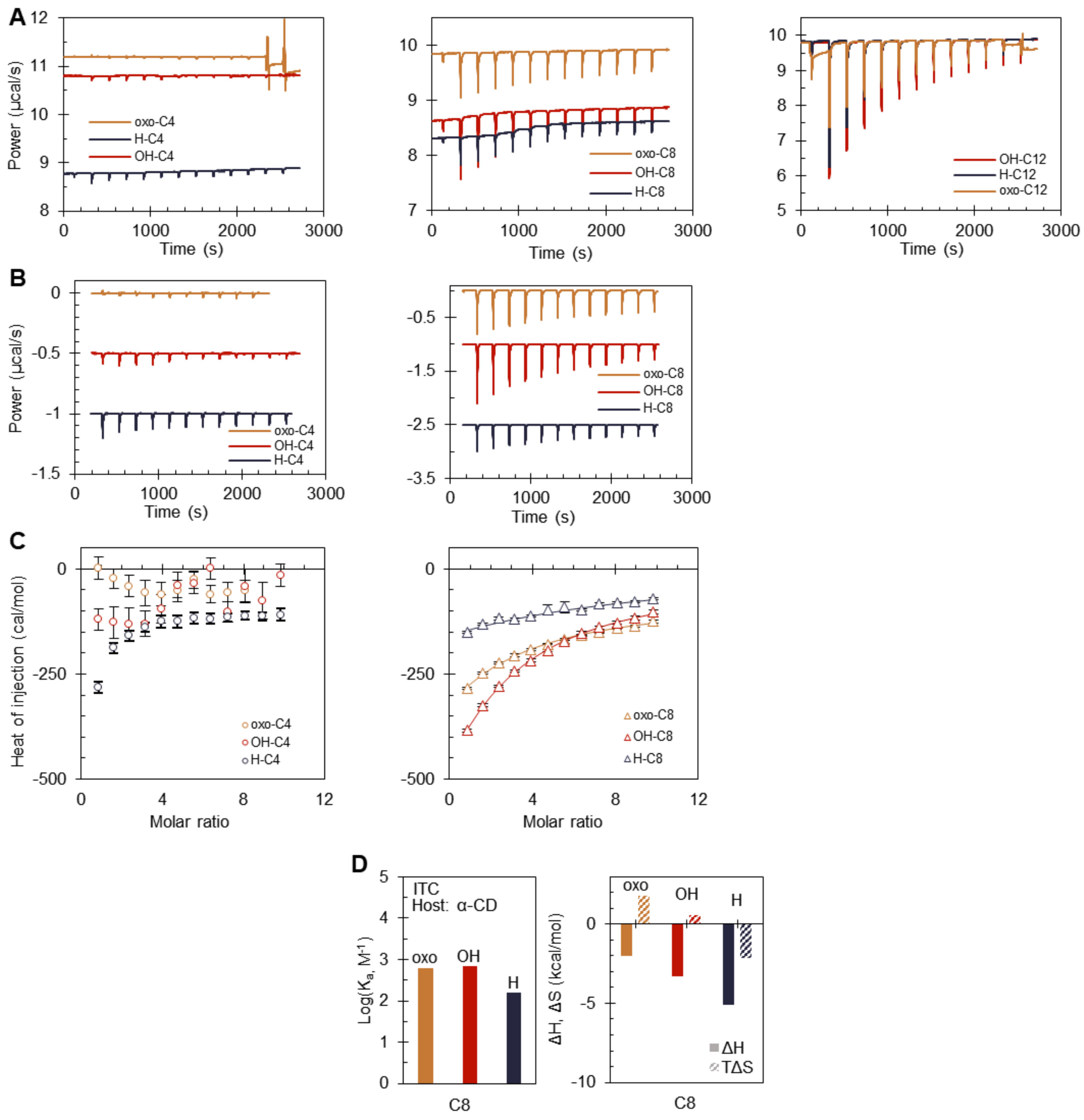

Figure S 28. **A** Raw thermograms of (left to right) C4-HSLs, C8-HSLs, C12-HSLs titrated against α-CD. **B** NITPIC-corrected thermograms with aberrant peaks removed for (left to right) C4-HSLs and C8-HSLs titrated against α-CD. **C** Integrated injection peaks of thermograms for (left to right) C4-HSLs and C8-HSLs. **D** Fitted binding constants (left) and thermodynamic parameters (right) for C8-HSLs titrated against α-CD.

3.3. Fluorescence Detected titrations

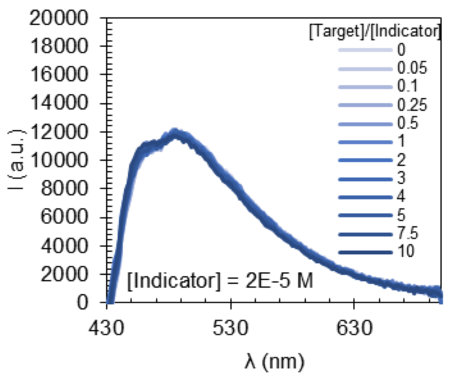

Figure S 29. Fluorescence measurements of control with H-C12-HSL added to only 2,6-ANS.

3.3.1. Indicator titrations

Table S 1. Binding constants obtained between hosts and 2,6-ANS.

**Host**	**Ka's (M^{-1})**	**$n_{replicates}$**	**Average**	**Log(K_a)**	**StDev**
β-CD	1080, 1020, 1050	3	1050	3.02	0.01
Mono(6-NH_2)-β-CD	643, 575, 609	3	609	2.78	0.02
Di(6-NH_2)-β-CD	3500, 3280, 3220	3	3333	3.52	0.02
2,6-DiMe-β-CD	6430, 8130, 5410	3	6657	3.82	0.09
TriMe-β-CD	1010, 1070, 1020	3	1033	3.01	0.01
Lys-β-CD	3010, 2410	2	2710	3.43	0.07
Di-Lys-β-CD	11700	1	11700	4.07	N/A
Mono(6-TMA)-β-CD	9750	1	9750	3.99	N/A
Di(6-TMA)-β-CD	1370	1	1370	3.14	N/A

A

[Host], M	[Indicator], M
0	5E-6
2.5E-6	5E-6
5E-6	5E-6
5E-5	5E-6
1E-4	5E-6
2E-4	5E-6
5E-4	5E-6
1E-3	5E-6
2E-3	5E-6
4E-3	5E-6
6E-3	5E-6
7.5E-3	5E-6

B

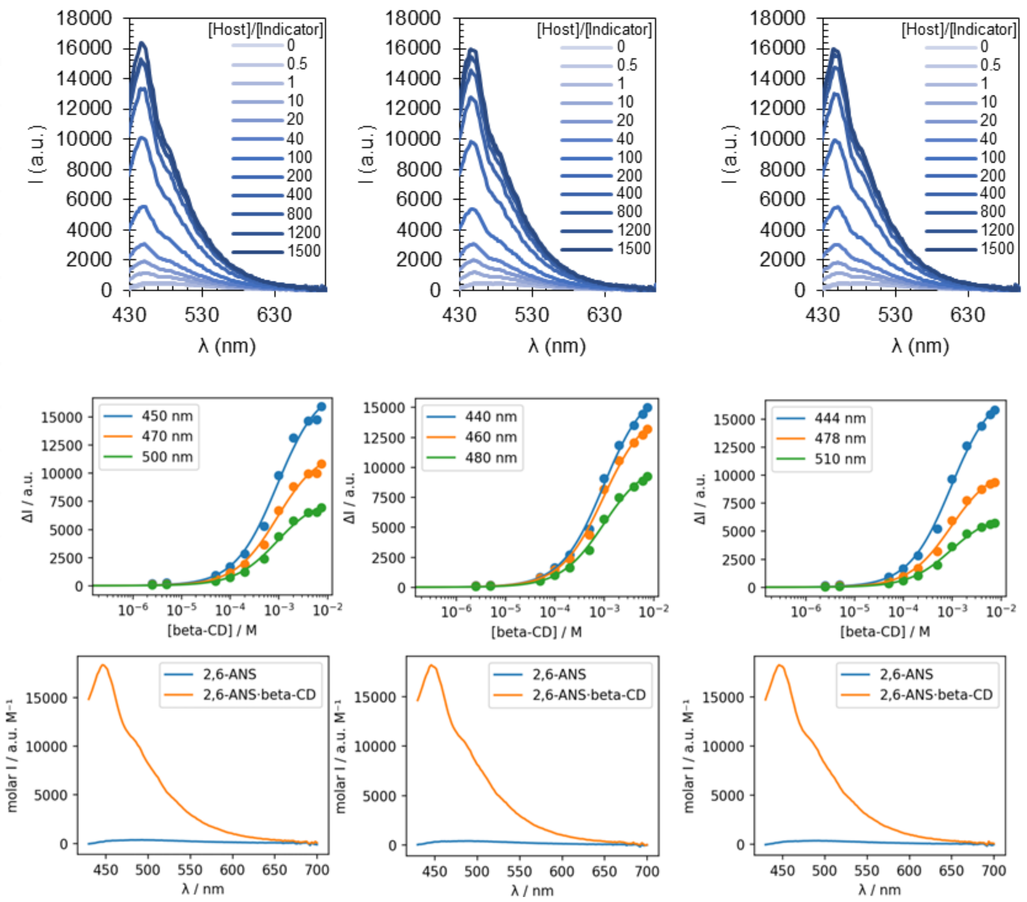

Figure S 30. **A** Host and indicator concentrations used for the titration of 2,6-ANS versus β-CD. **B** From top to bottom: fluorescence spectra, intensity traces at given wavelengths across the titration, and corresponding species' fluorescence emission spectra in the optimized model. Left to right: replicates.

A

[Host], M	[Indicator], M
0	5E-6
2.5E-6	5E-6
5E-6	5E-6
5E-5	5E-6
1E-4	5E-6
2E-4	5E-6
5E-4	5E-6
1E-3	5E-6
2E-3	5E-6
4E-3	5E-6
6E-3	5E-6
7.5E-3	5E-6

B

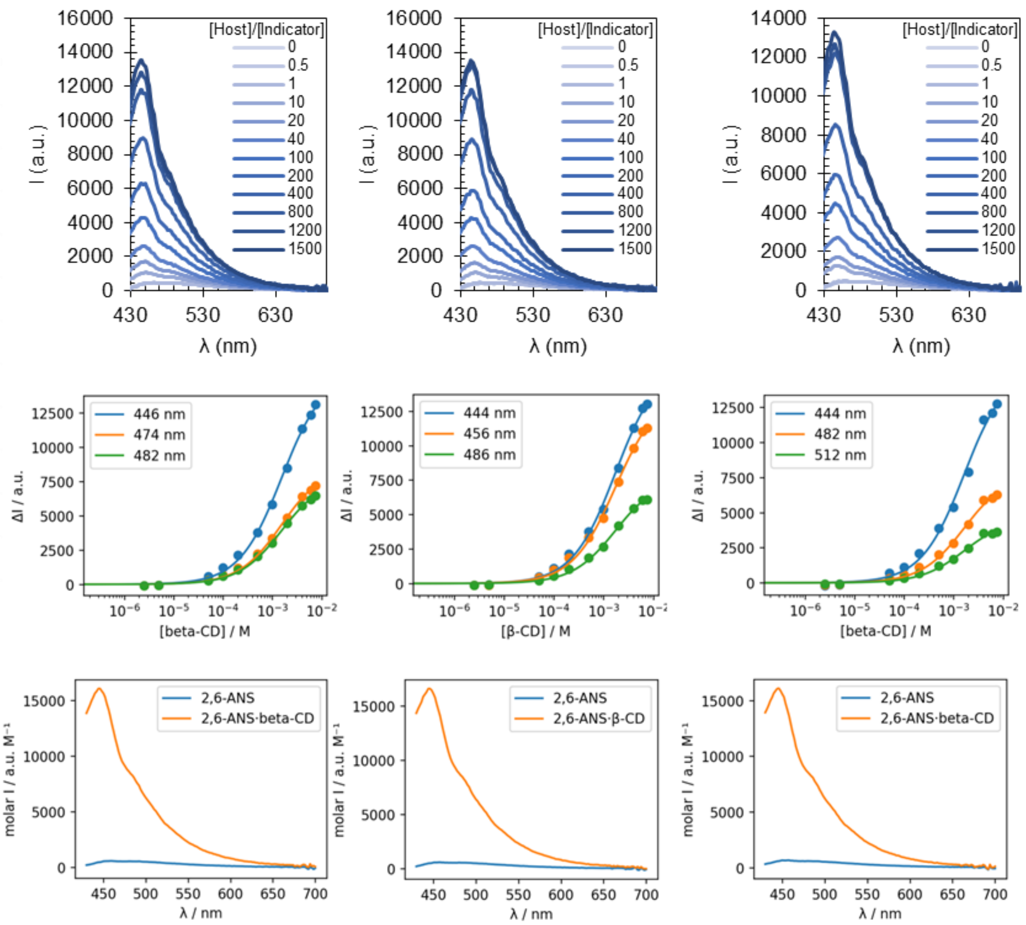

Figure S 31. **A** Host and indicator concentrations used for the titration of 2,6-ANS versus Mono(6-NH2)-β-CD. **B** From top to bottom: fluorescence spectra, intensity traces at given wavelengths across the titration, and corresponding species' fluorescence emission spectra in the optimized model. Left to right: replicates.

A

[Host], M	[Indicator], M
0	5E-6
1E-6	5E-6
2.5E-6	5E-6
5E-6	5E-6
1E-5	5E-6
2.5E-5	5E-6
5E-5	5E-6
1E-4	5E-6
2.5E-4	5E-6
5E-4	5E-6
1E-3	5E-6
2E-3	5E-6

B

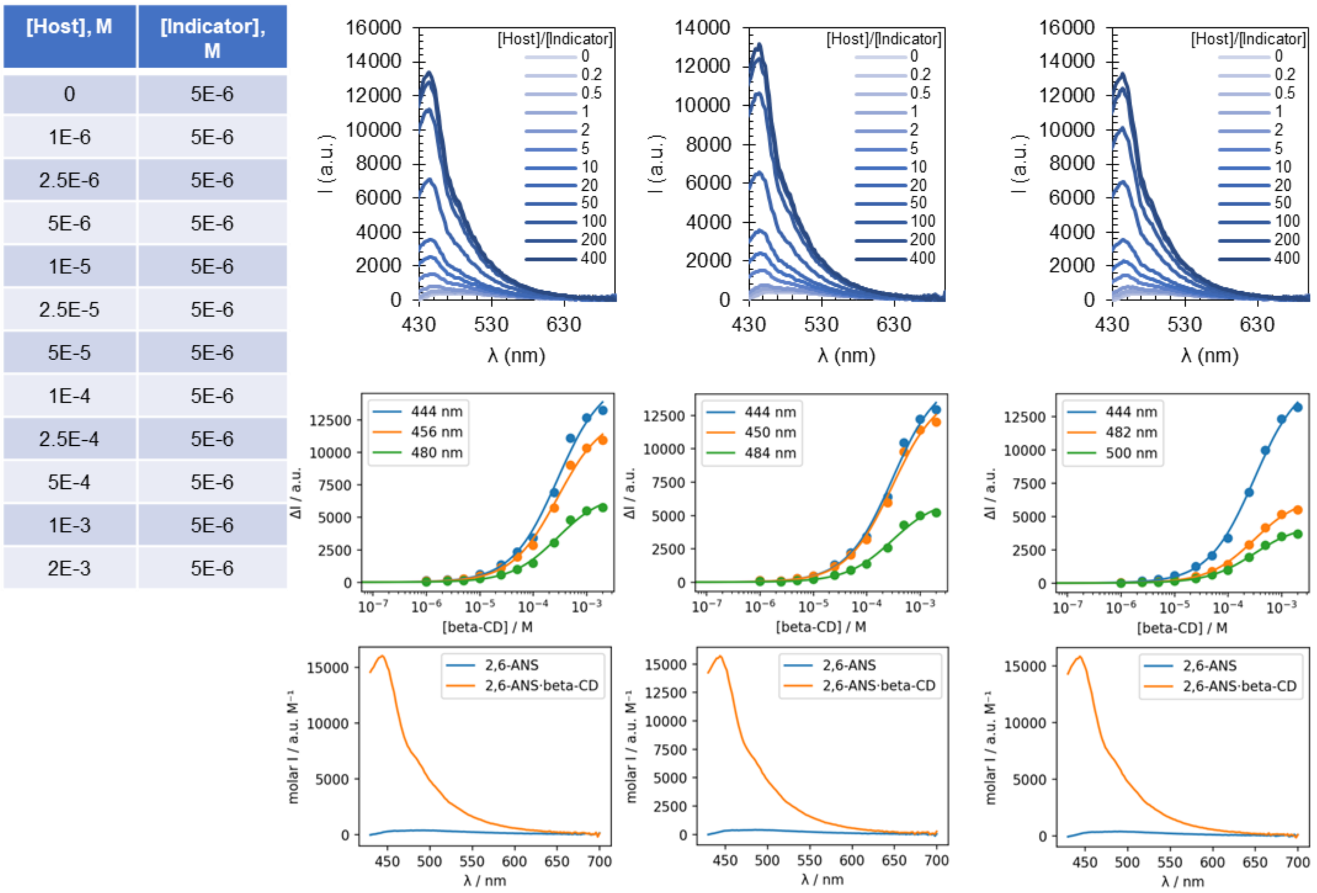

Figure S 32. **A** Host and indicator concentrations used for the titration of 2,6-ANS versus Di(6-NH2)-β-CD. **B** From top to bottom: fluorescence spectra, intensity traces at given wavelengths across the titration, and corresponding species' fluorescence emission spectra in the optimized model. Left to right: replicates.

[Host], M	[Indicator], M
0	1E-6
1E-5	1E-6
5E-5	1E-6
1E-4	1E-6
1.5E-4	1E-6
2E-4	1E-6

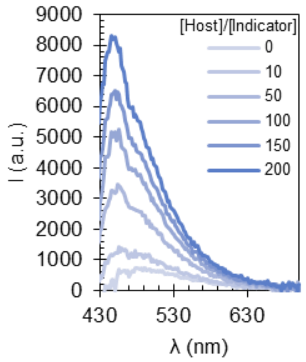

[Host], M	[Indicator], M
0	5E-6
5E-5	5E-6
1E-4	5E-6
2.5E-4	5E-6
5E-4	5E-6
7E-4	5E-6

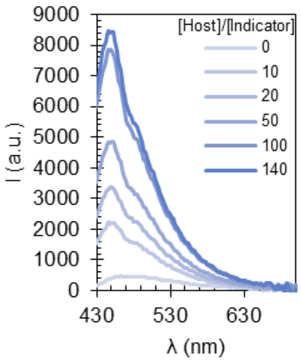

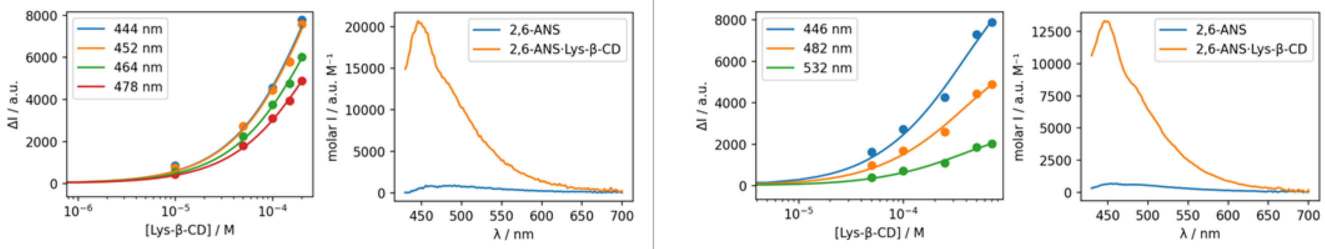

Figure S 33. For two titrations at different concentrations, separated by grey line, clockwise from top left: Host and indicator concentrations used for the titration of 2,6-ANS versus MonoLys-β-CD,

fluorescence spectra, intensity traces at given wavelengths across the titration, and corresponding species' fluorescence emission spectra in the optimized model.

[Host], M	[Indicator], M
0	1E-6
1E-5	1E-6
5E-5	1E-6
1E-4	1E-6
1.5E-4	1E-6
2E-4	1E-6

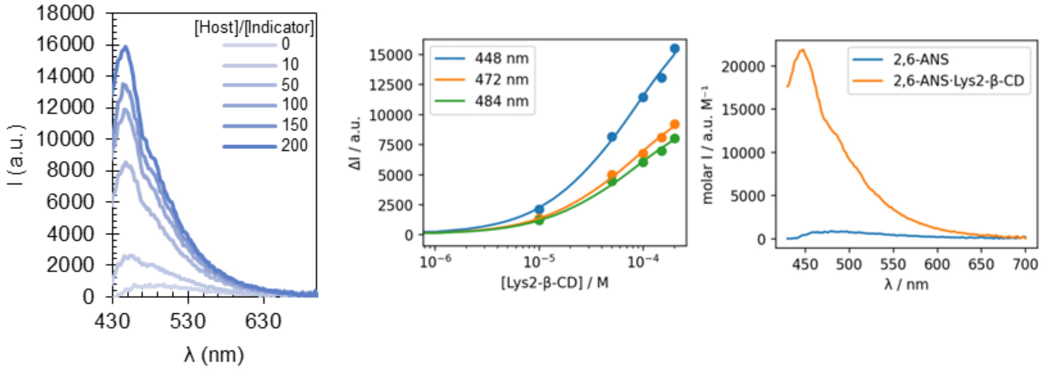

Figure S 34. Left to right: Host and indicator concentrations used for the titration of 2,6-ANS versus DiLys-β-CD, fluorescence spectra, intensity traces at given wavelengths across the titration, and corresponding species' fluorescence emission spectra in the optimized model.

[Host], M	[Indicator], M
0	2E-5
1E-5	2E-5
2E-5	2E-5
4E-5	2E-5
1E-4	2E-5
2E-4	2E-5

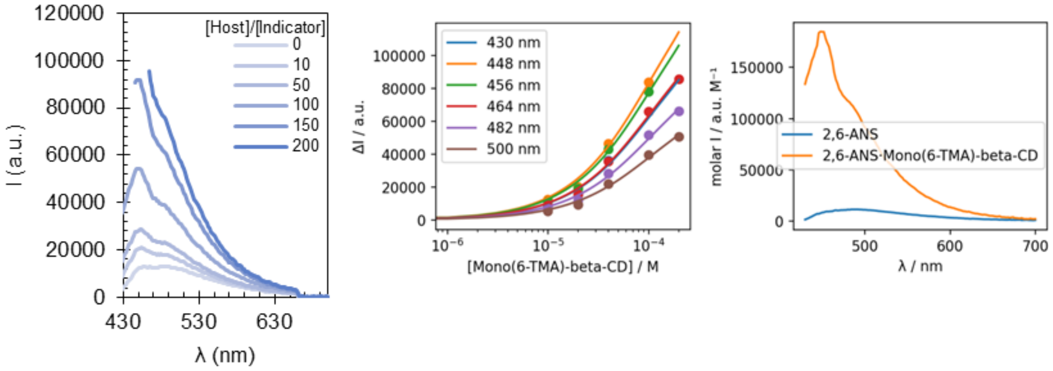

Figure S 35. Left to right: Host and indicator concentrations used for the titration of 2,6-ANS versus Mono(6-TMA)-β-CD, fluorescence spectra, intensity traces at given wavelengths across the titration, and corresponding species' fluorescence emission spectra in the optimized model.

[Host], M	[Indicator], M
0	1E-6
1E-5	1E-6
5E-5	1E-6
1E-4	1E-6
1.5E-4	1E-6
2E-4	1E-6

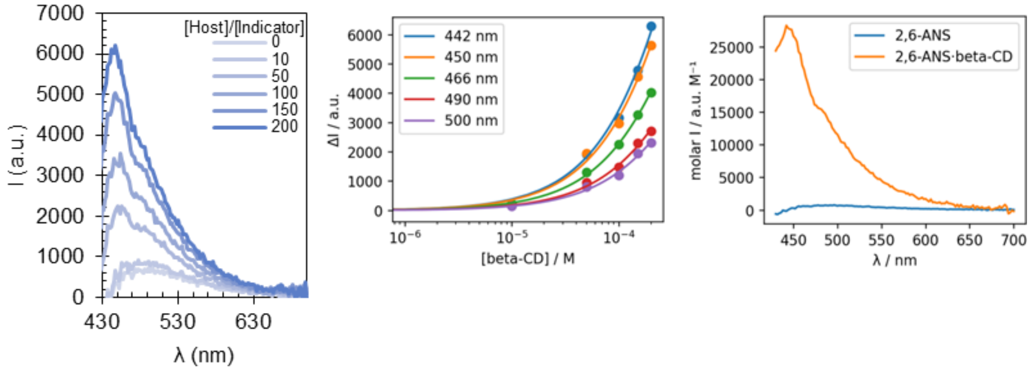

Figure S 36. Left to right: Host and indicator concentrations used for the titration of 2,6-ANS versus Di(6-TMA)-β-CD, fluorescence spectra, intensity traces at given wavelengths across the titration, and corresponding species' fluorescence emission spectra in the optimized model.

A

[Host], M	[Indicator], M
0	1E-6
1E-6	1E-6
1E-5	1E-6
2E-5	1E-6
5E-5	1E-6
1E-4	1E-6
2E-4	1E-6
5E-4	1E-6
8E-4	1E-6
1E-3	1E-6
2E-3	1E-6
3E-3	1E-6

B

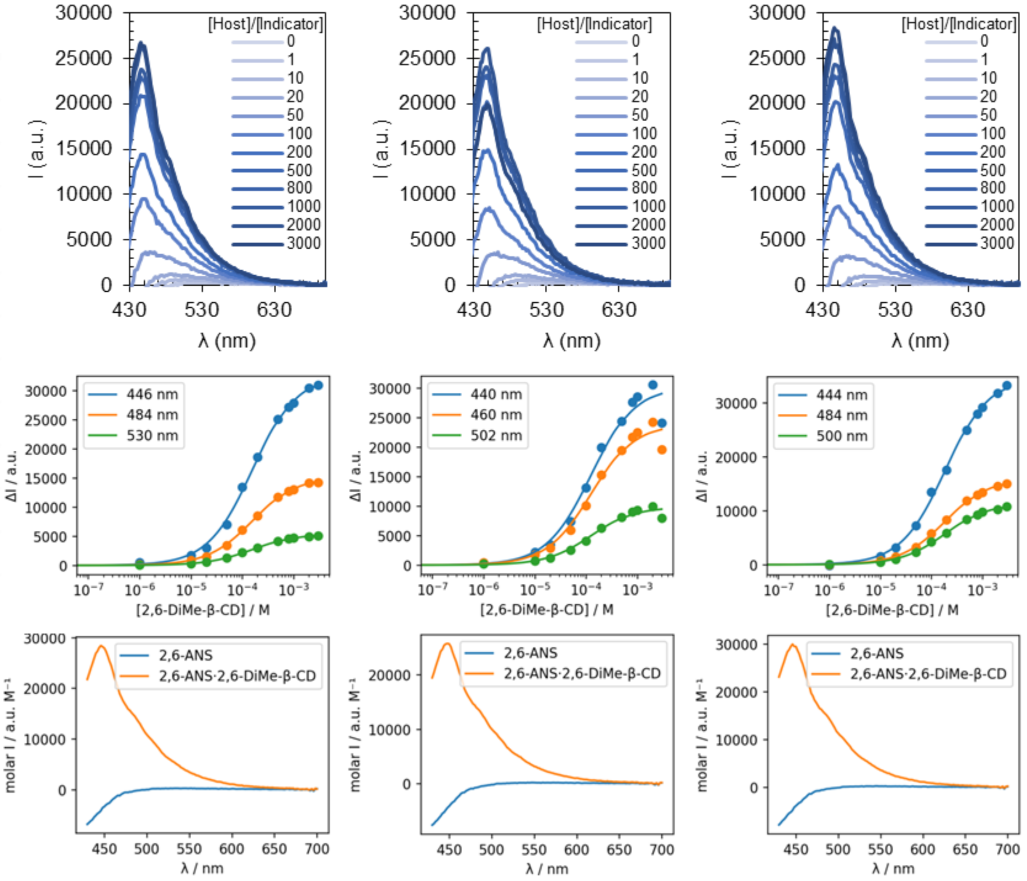

Figure S 37. **A** Host and indicator concentrations used for the titration of 2,6-ANS versus 2,6-DiMe-β-CD. **B** From top to bottom: fluorescence spectra, intensity traces at given wavelengths across the titration, and corresponding species' fluorescence emission spectra in the optimized model. Left to right: replicates.

A

[Host], M	[Indicator], M
0	1E-6
1E-6	1E-6
1E-5	1E-6
5E-5	1E-6
1E-4	1E-6
2E-4	1E-6
5E-4	1E-6
8E-4	1E-6
1E-3	1E-6
2E-3	1E-6
4E-3	1E-6
5E-3	1E-6

B

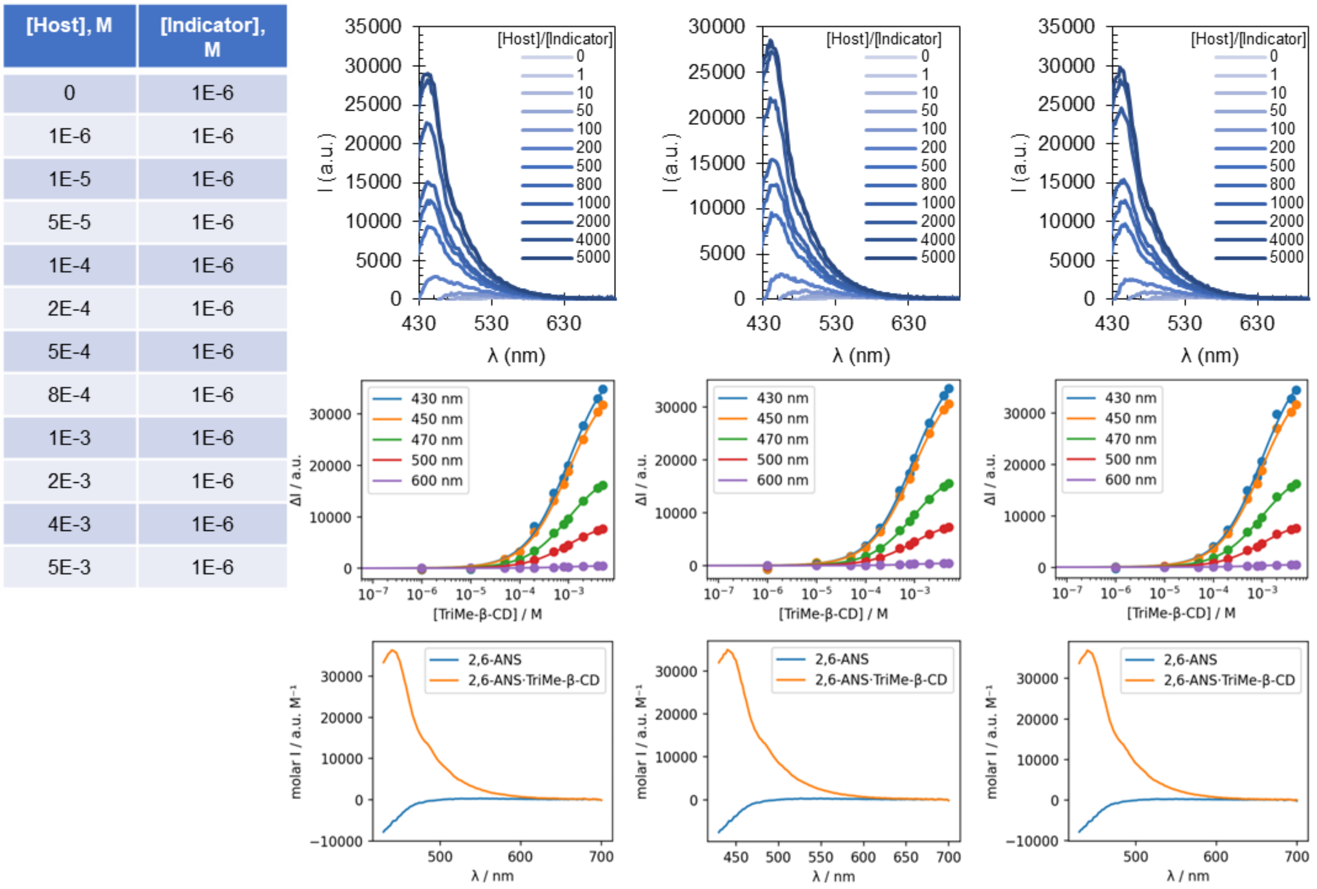

Figure S 38. **A** Host and indicator concentrations used for the titration of 2,6-ANS versus TriMe-β-CD. **B** From top to bottom: fluorescence spectra, intensity traces at given wavelengths across the titration, and corresponding species' fluorescence emission spectra in the optimized model. Left to right: replicates.

3.3.2. Indicator displacement assays

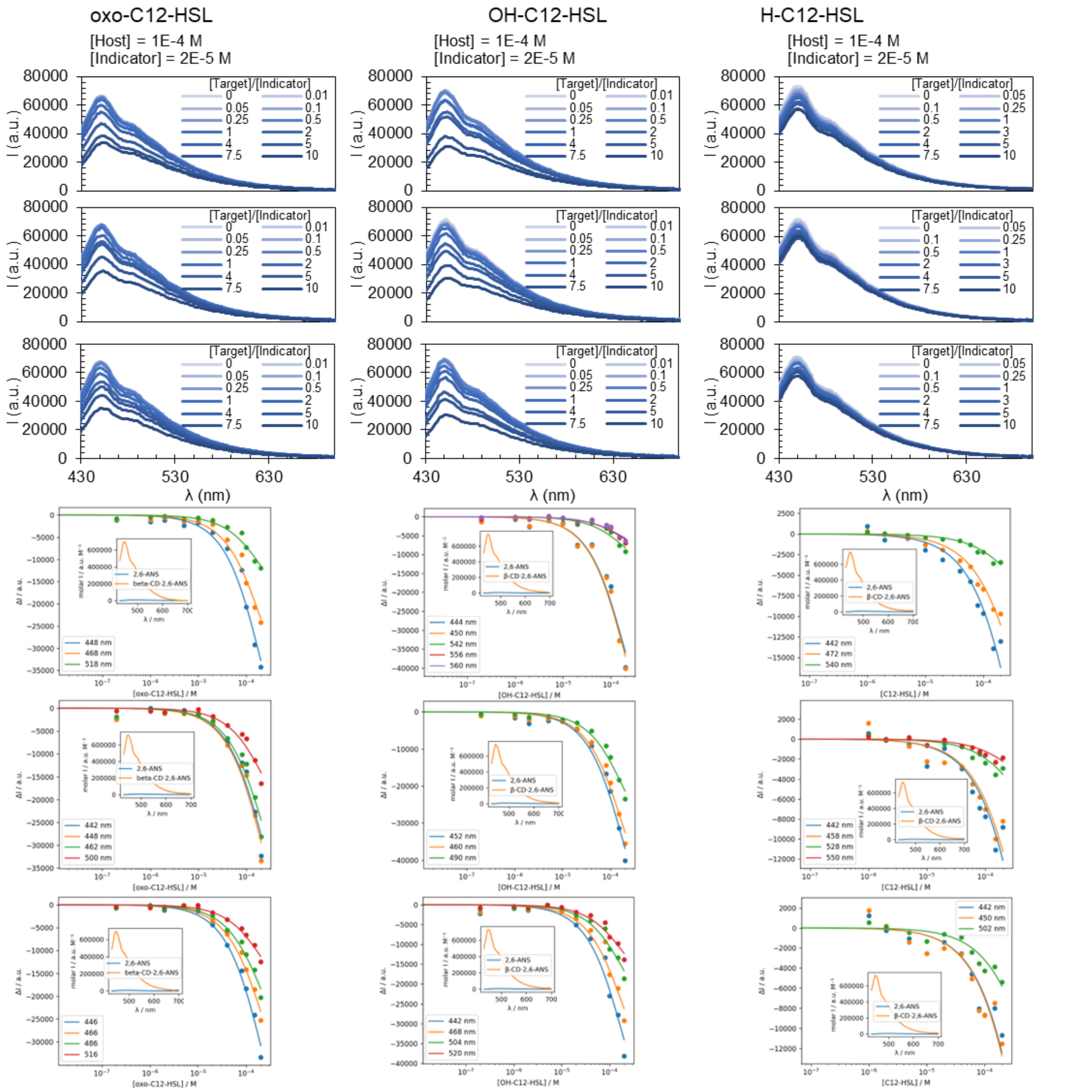

Figure S 39. IDA titrations of (left to right) oxo-C12-HSL, OH-C12-HSL, H-C12-HSL versus β-CD. From top to bottom: spectra of replicates, intensity traces at given wavelengths across the titration (inset: corresponding species' fluorescence emission spectra in the optimized model). For H-C12-HSL fitting, the unbound 2,6-ANS spectrum was fixed, obtained from the corresponding host's OH-C12-HSL averaged IDA model.

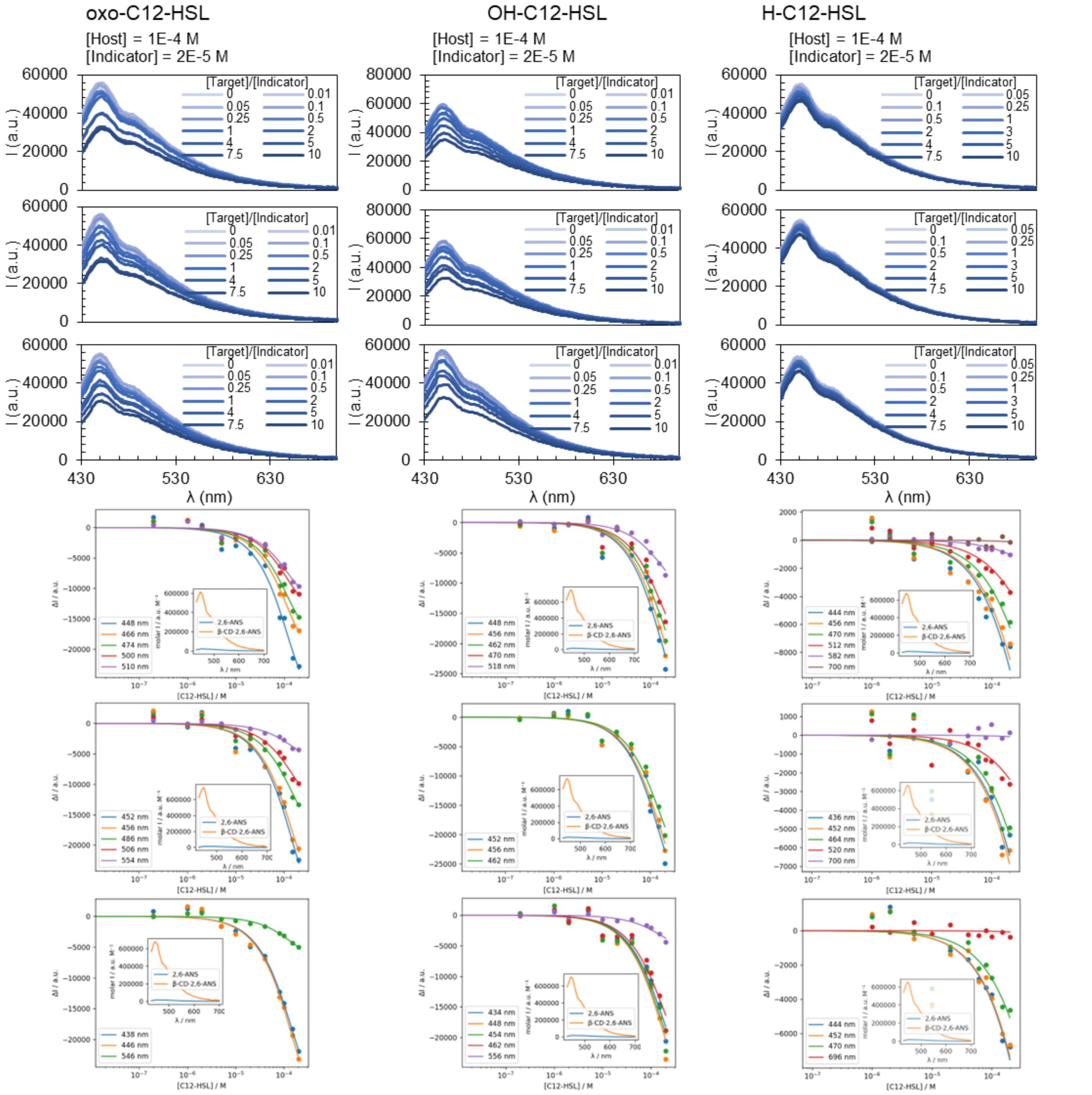

Figure S 40. IDA titrations of (left to right) oxo-C12-HSL, OH-C12-HSL, H-C12-HSL versus Mono(6-NH_2)-β-CD. From top to bottom: spectra of replicates, intensity traces at given wavelengths across the titration (inset: corresponding species' fluorescence emission spectra in the optimized model). For H-C12-HSL and OH-C12-HSL fitting, the unbound 2,6-ANS spectrum was fixed, obtained from the corresponding host's OH-C12-HSL averaged IDA model.

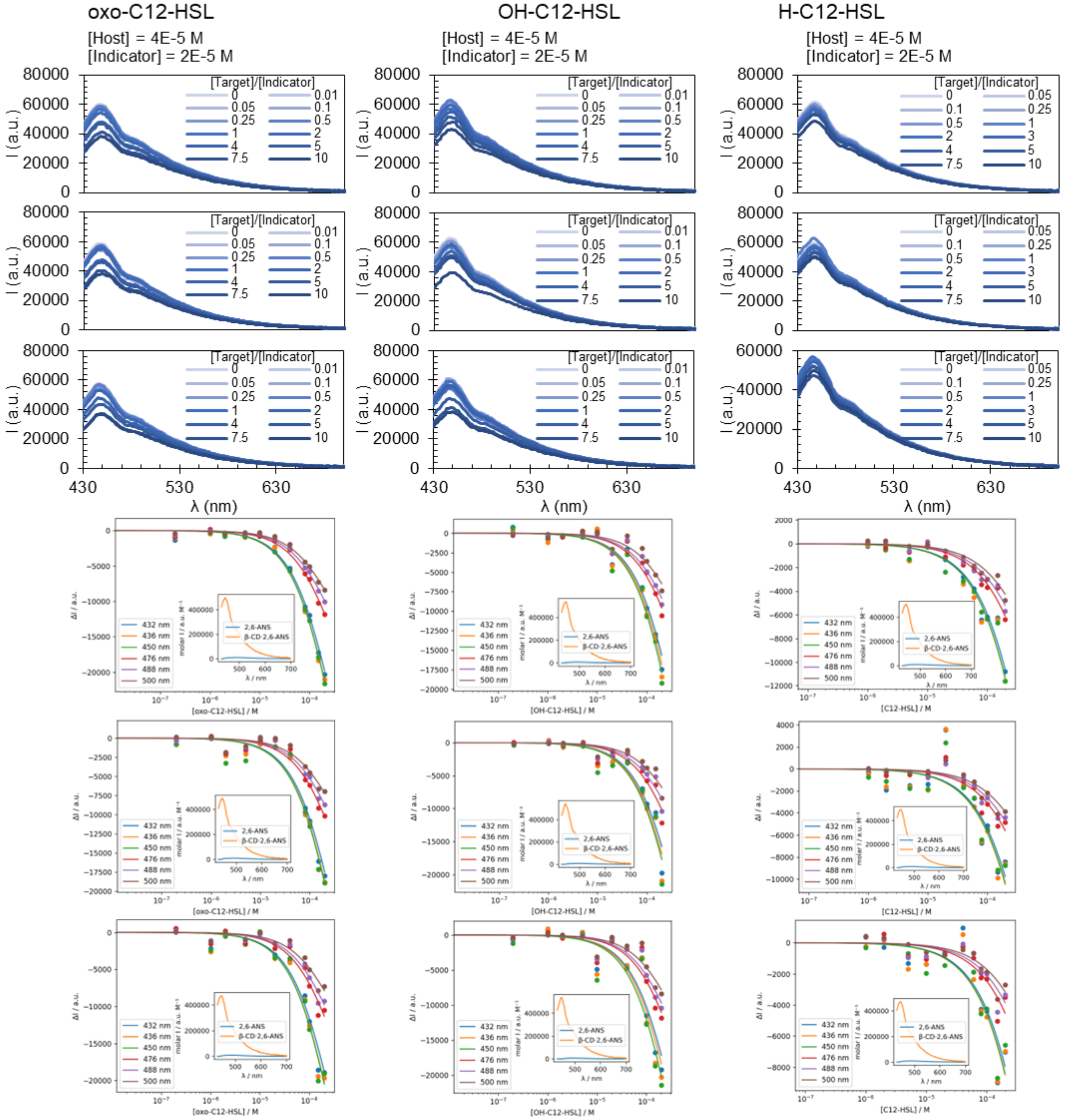

Figure S 41. IDA titrations of (left to right) oxo-C12-HSL, OH-C12-HSL, H-C12-HSL versus Di(6-NH_2)-β-CD. From top to bottom: spectra of replicates, intensity traces at given wavelengths across the titration (inset: corresponding species' fluorescence emission spectra in the optimized model). For H-C12-HSL and OH-C12-HSL fitting, the unbound 2,6-ANS spectrum was fixed, obtained from the corresponding host's oxo-C12-HSL averaged IDA model.

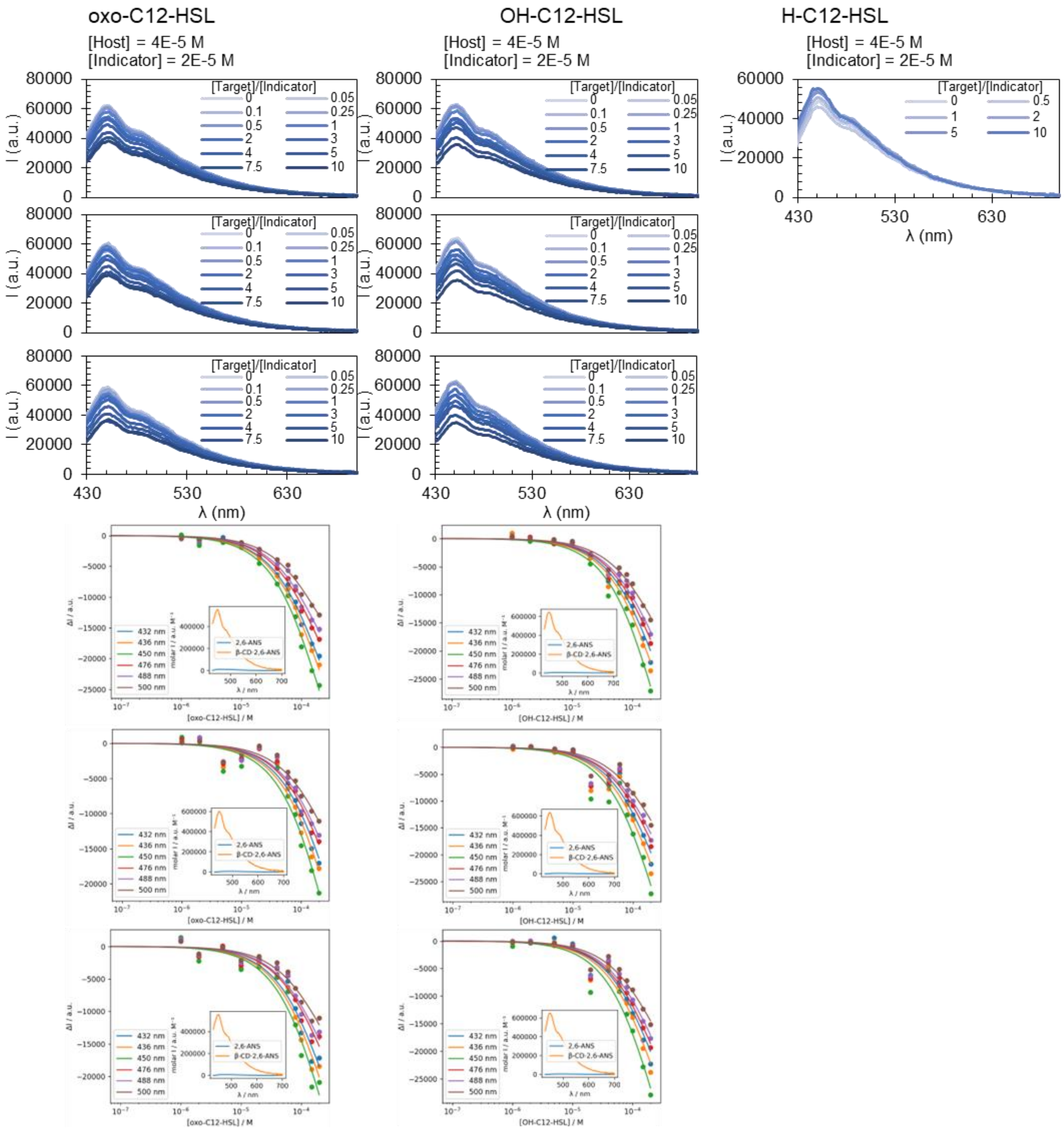

Figure S 42. IDA titrations of (left to right) oxo-C12-HSL, OH-C12-HSL, H-C12-HSL versus MonoLys-β-CD. From top to bottom: spectra of replicates, intensity traces at given wavelengths across the titration (inset: corresponding species' fluorescence emission spectra in the optimized model). Results for H-C12-HSL could not be fitted satisfactorily.

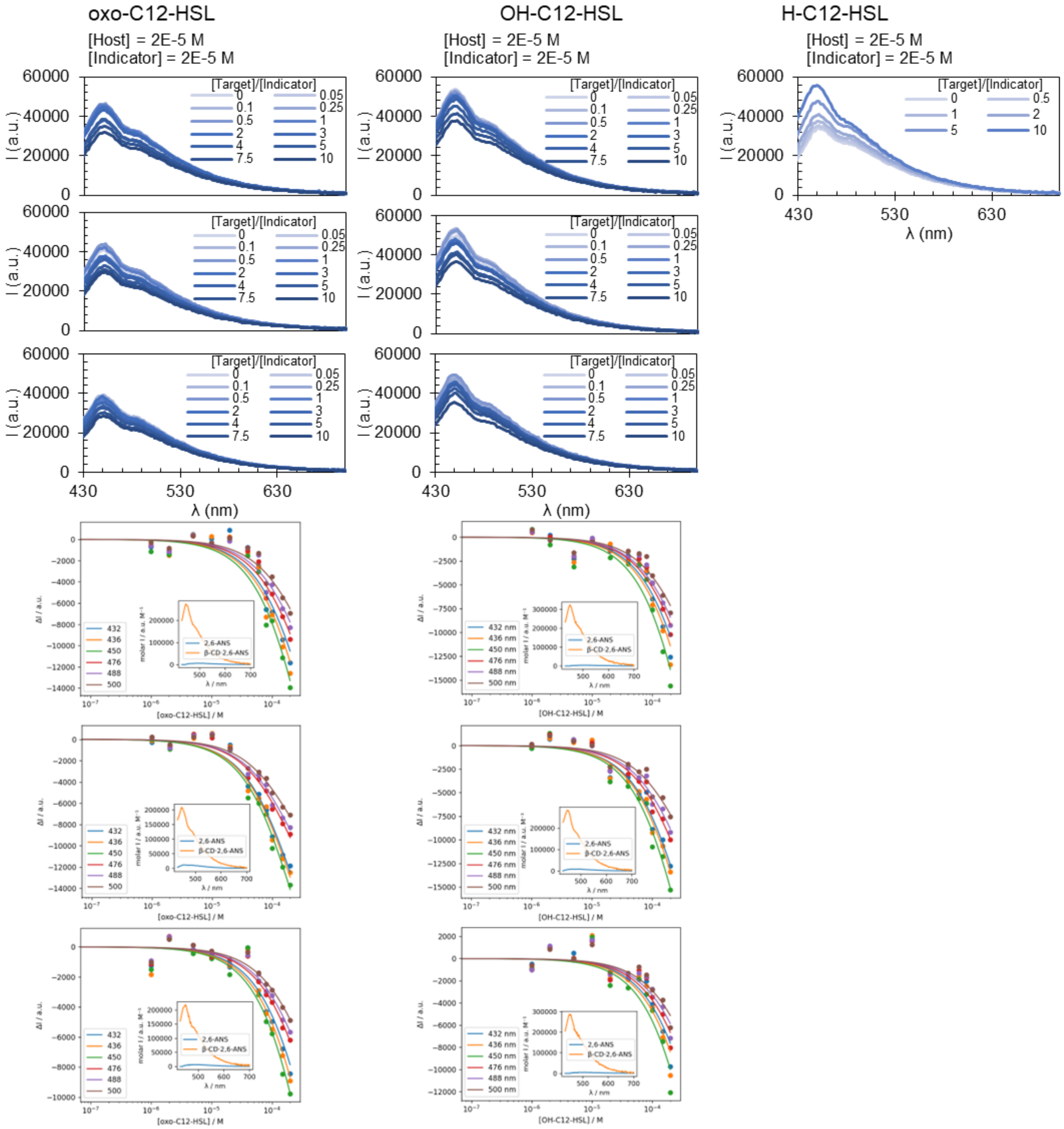

Figure S 43. IDA titrations of (left to right) oxo-C12-HSL, OH-C12-HSL, H-C12-HSL versus DiLys-β-CD. From top to bottom: spectra of replicates, intensity traces at given wavelengths across the titration (inset: corresponding species' fluorescence emission spectra in the optimized model). Results for H-C12-HSL could not be fitted satisfactorily.

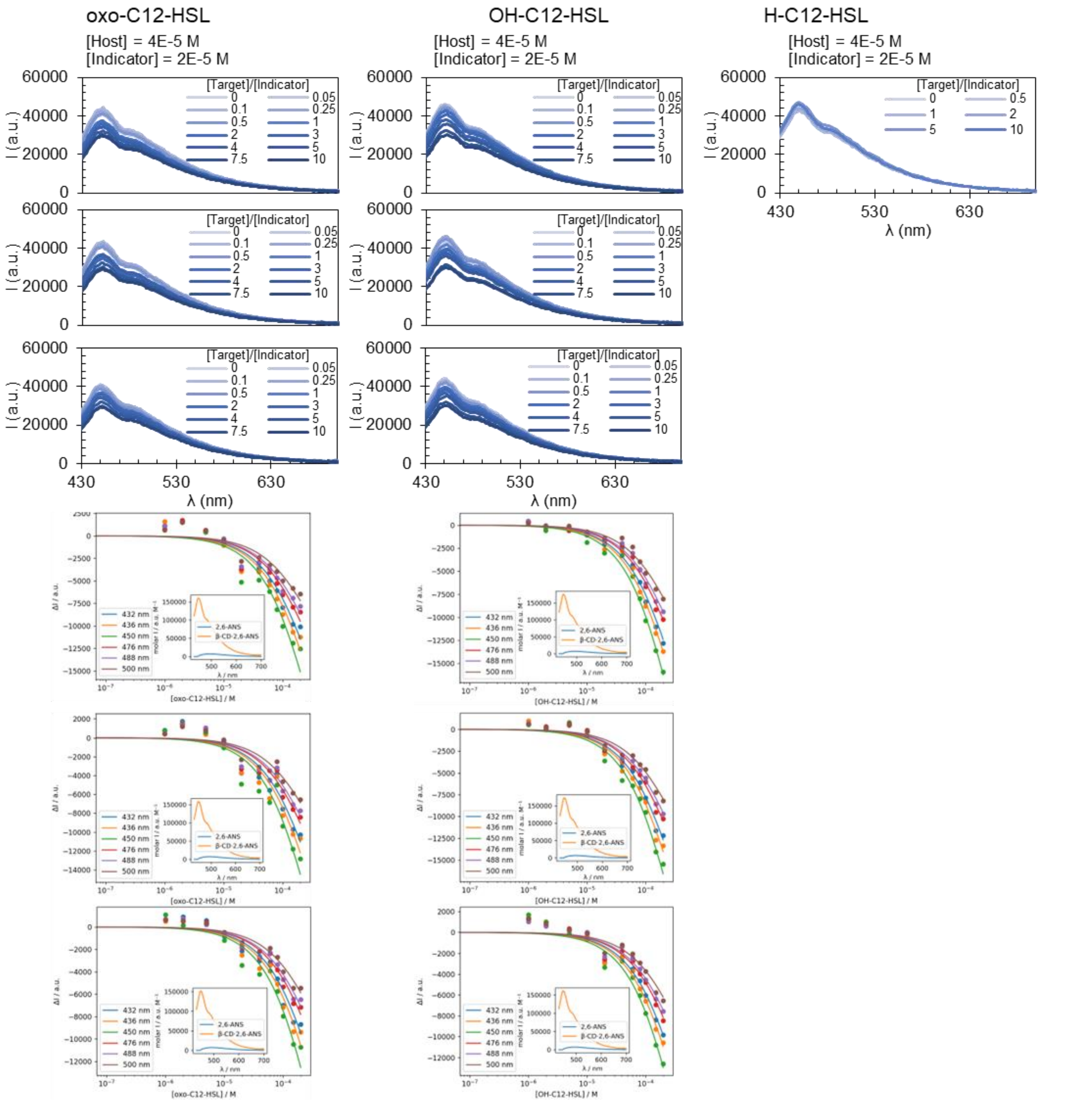

Figure S 44. IDA titrations of (left to right) oxo-C12-HSL, OH-C12-HSL, H-C12-HSL versus Mono(6-TMA)-β-CD. From top to bottom: spectra of replicates, intensity traces at given wavelengths across the titration (inset: corresponding species' fluorescence emission spectra in the optimized model). For oxo-C12-HSL fitting, the unbound 2,6-ANS spectrum was fixed, obtained from the corresponding host's OH-C12-HSL averaged IDA model. Results for H-C12-HSL could not be fitted satisfactorily.

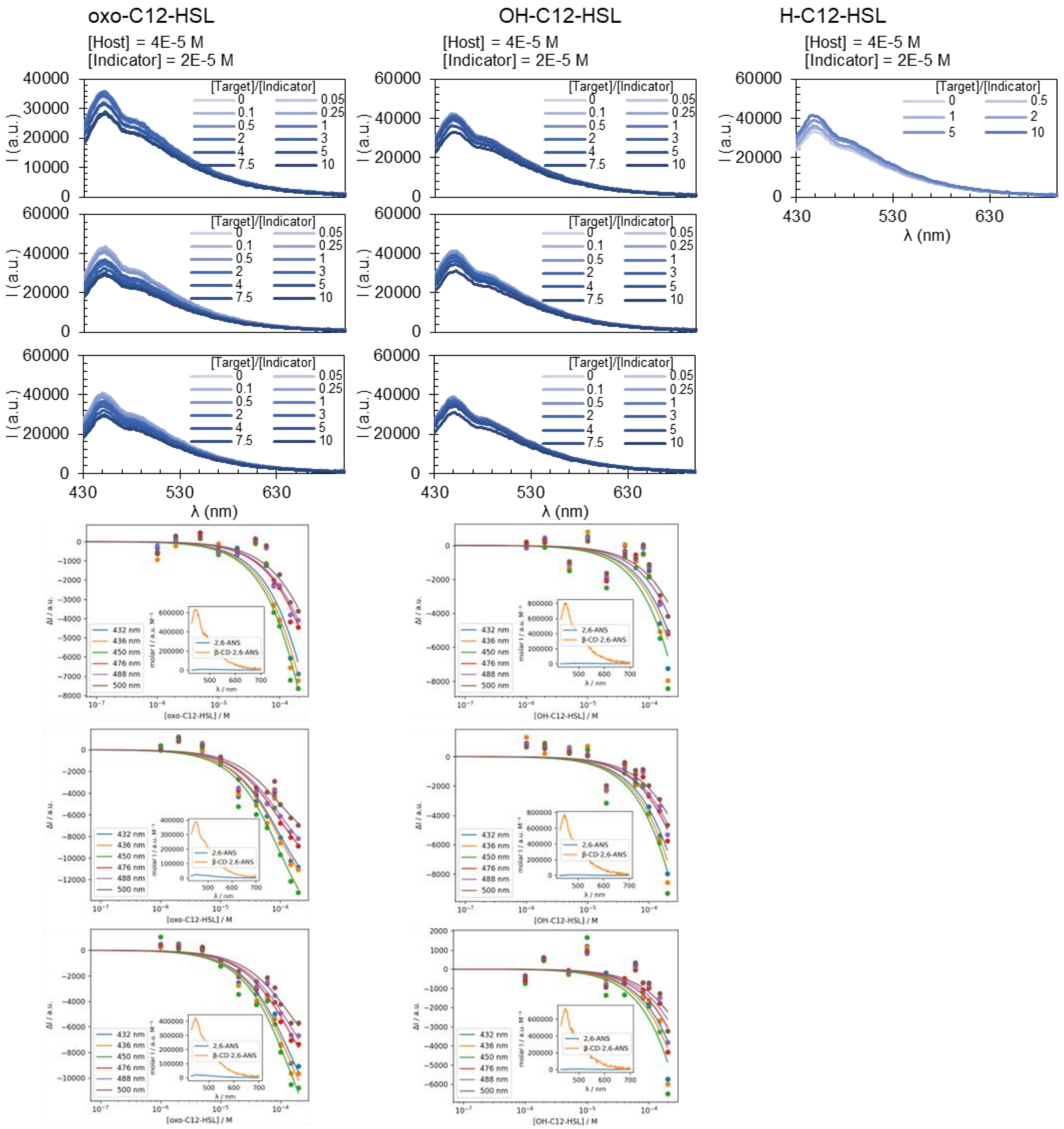

Figure S 45. IDA titrations of (left to right) oxo-C12-HSL, OH-C12-HSL, H-C12-HSL versus Di(6-TMA)-β-CD. From top to bottom: spectra of replicates, intensity traces at given wavelengths across the titration (inset: corresponding species' fluorescence emission spectra in the optimized model). Results for H-C12-HSL could not be fitted satisfactorily.

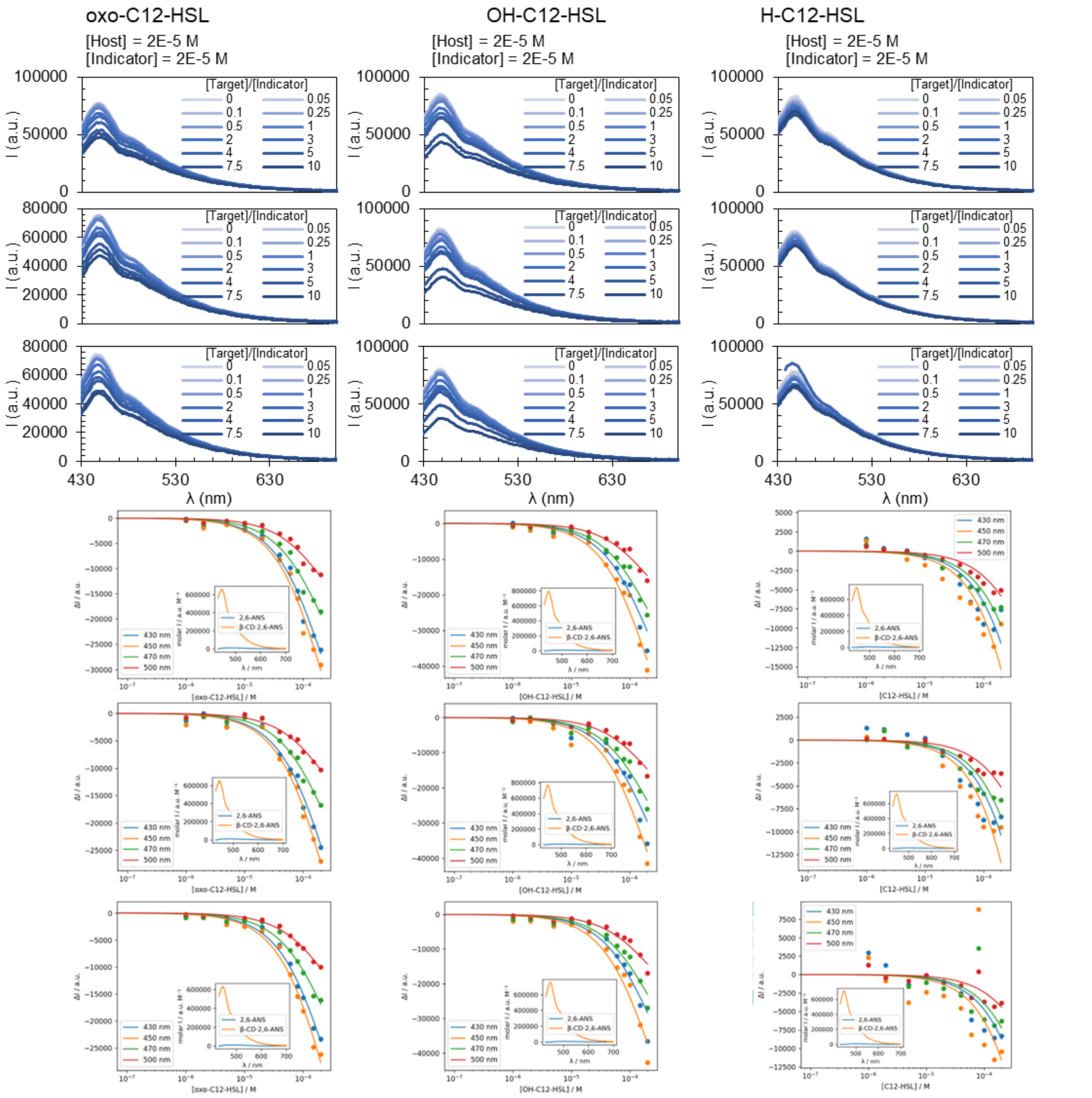

Figure S 46. IDA titrations of (left to right) oxo-C12-HSL, OH-C12-HSL, H-C12-HSL versus 2,6-DiMe-β-CD. From top to bottom: spectra of replicates, intensity traces at given wavelengths across the titration (inset: corresponding species' fluorescence emission spectra in the optimized model). For H-C12-HSL fitting, the unbound 2,6-ANS spectrum was fixed, obtained from the corresponding host's OH-C12-HSL averaged IDA model.

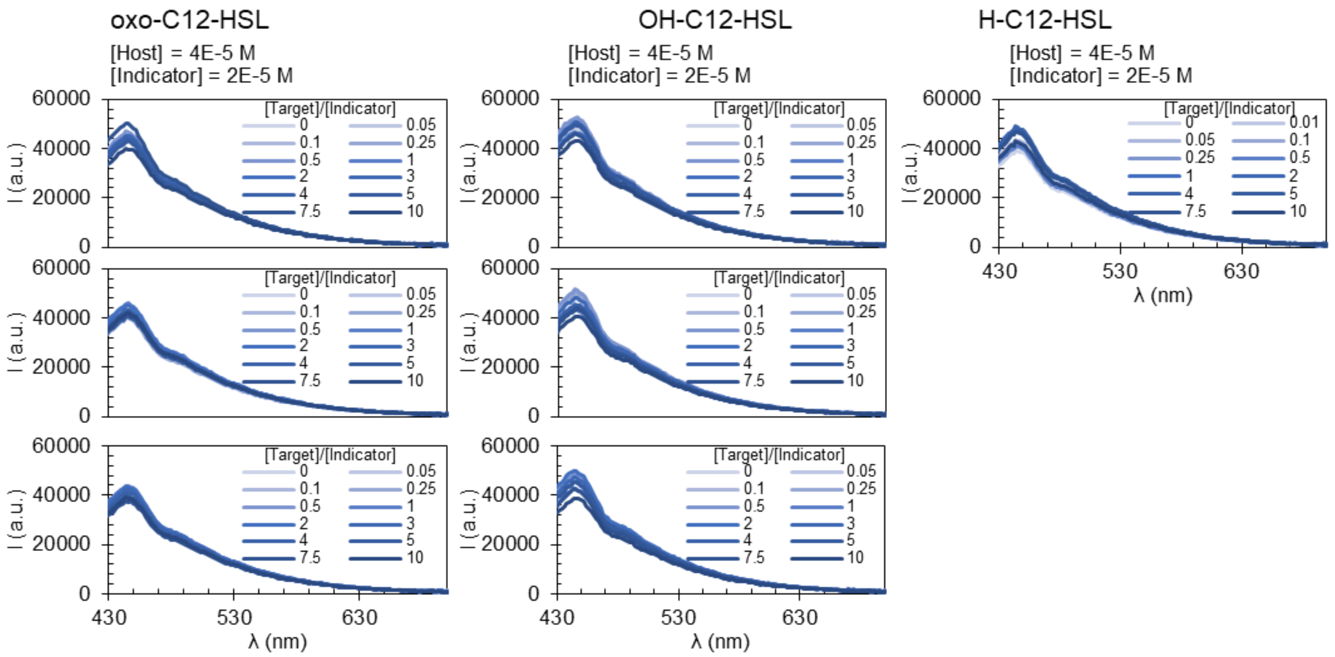

Figure S 47. IDA titrations of (left to right) oxo-C12-HSL, OH-C12-HSL, H-C12-HSL versus TriMe-β-CD. From top to bottom: spectra of replicates, intensity traces at given wavelengths across the titration (inset: corresponding species' fluorescence emission spectra in the optimized model). All titrations were deemed inconclusive in terms of binding constant.

A

[Target], M	[Host], M	[Indicator], M
0	1E-4	2E-5
1E-5	1E-4	2E-5
2E-5	1E-4	2E-5
1E-4	1E-4	2E-5
2E-4	1E-4	2E-5
5E-4	1E-4	2E-5

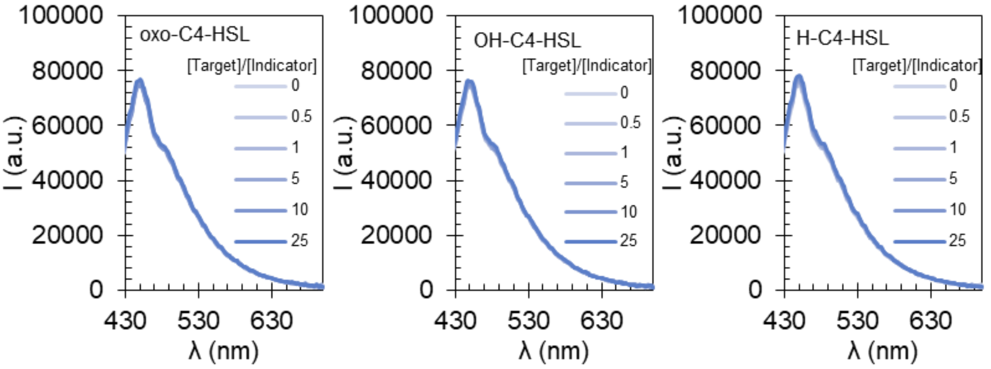

B

[Target], M	[Host], M	[Indicator], M
0	1E-4	2E-5
1E-5	1E-4	2E-5
2E-5	1E-4	2E-5
1E-4	1E-4	2E-5
2E-4	1E-4	2E-5
5E-4	1E-4	2E-5

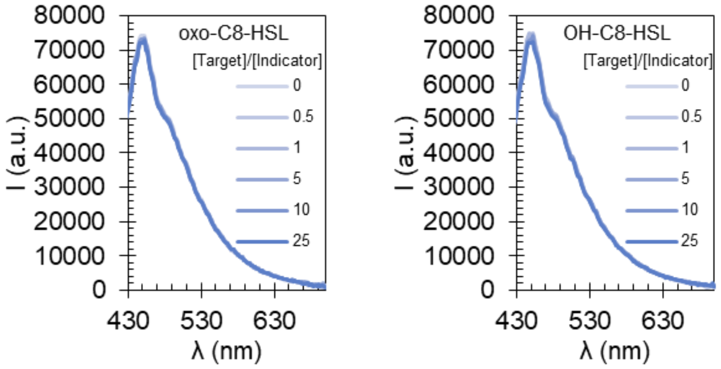

C

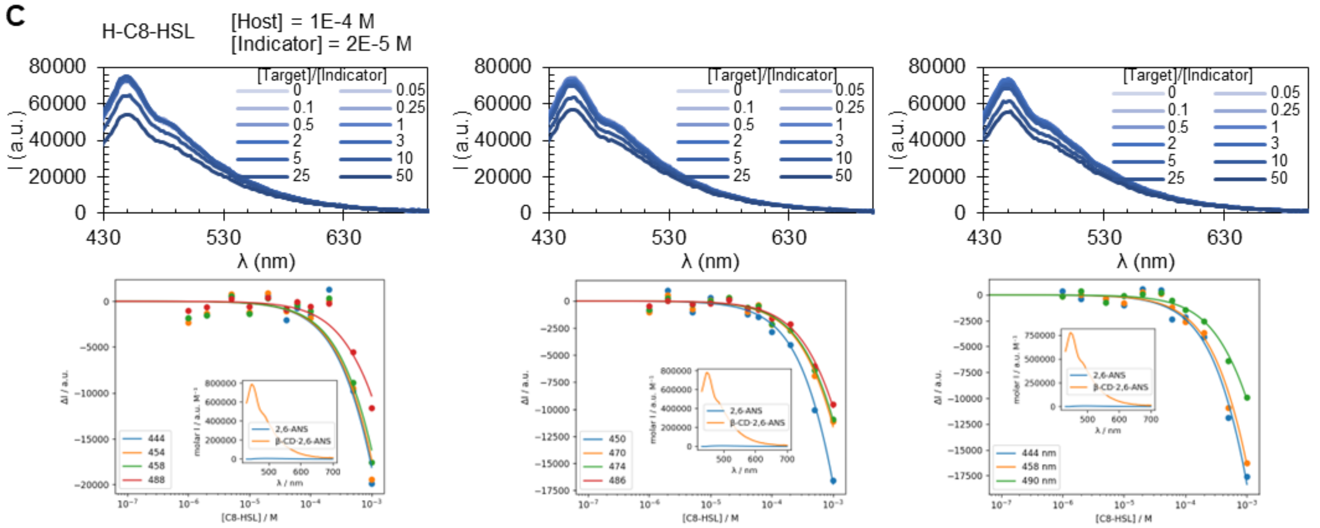

Figure S 48. **A** Concentrations of target, host and indicator for IDAs of β-CD against C4-HSLs; spectra of IDAs. Results for these HSLs could not be fitted satisfactorily. **B** Concentrations of target, host and indicator for IDAs of β-CD against oxo-C8-HSL and OH-C8-HSL; spectra of IDAs. Results for these HSLs could not be fitted satisfactorily. **C** IDA titration replicates of β-CD against H-C8-HSL; intensity traces at given wavelengths across the titration (inset: corresponding species' fluorescence emission spectra in the optimized model). The unbound 2,6-ANS spectrum was fixed, obtained from the corresponding host's OH-C12-HSL averaged IDA model.

A

[Target], M	[Host], M	[Indicator], M
0	1E-4	2E-5
2E-5	1E-4	2E-5
1E-4	1E-4	2E-5
2E-4	1E-4	2E-5
4E-4	1E-4	2E-5
1E-3	1E-4	2E-5

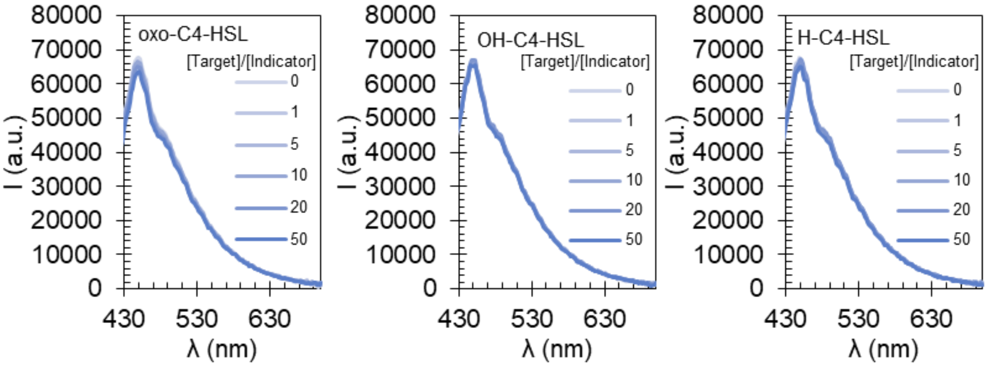

B

[Target], M	[Host], M	[Indicator], M
0	1E-4	2E-5
1E-5	1E-4	2E-5
2E-5	1E-4	2E-5
1E-4	1E-4	2E-5
2E-4	1E-4	2E-5
1E-3	1E-4	2E-5

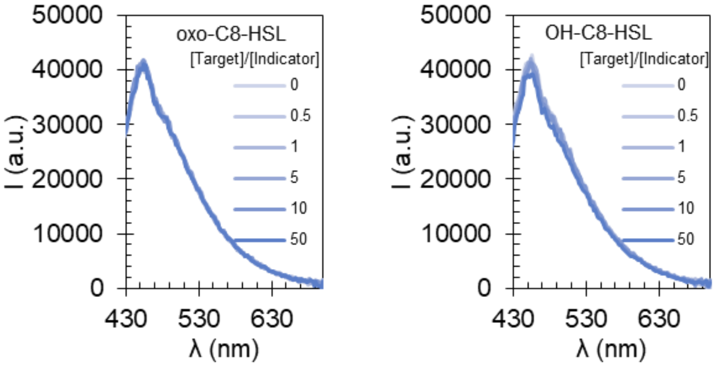

C

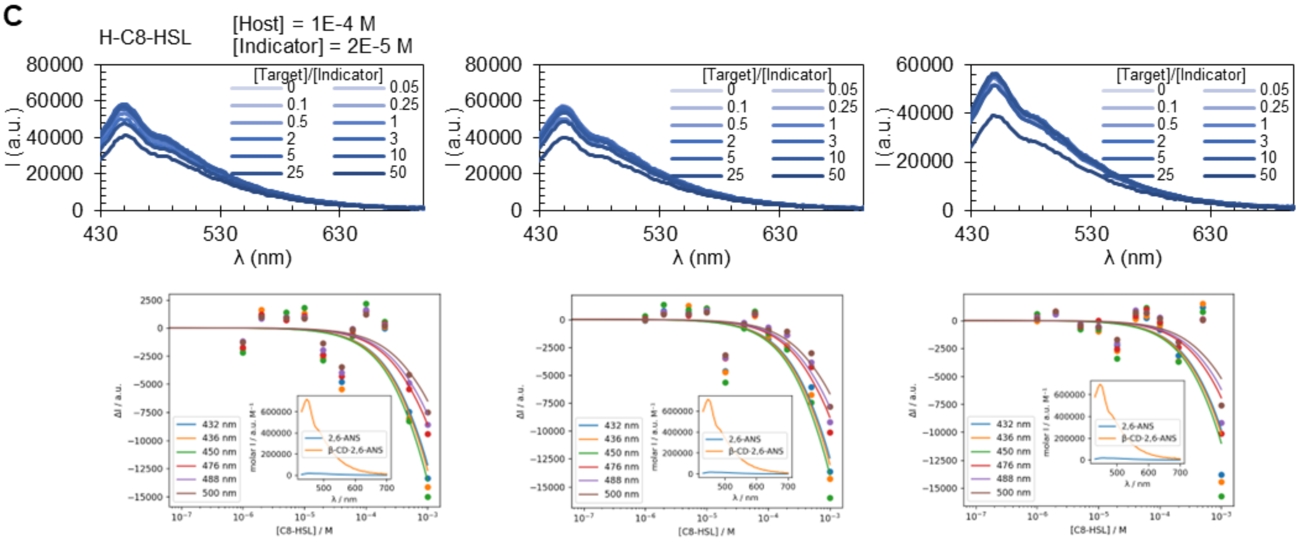

Figure S 49. **A** Concentrations of target, host and indicator for IDAs of Mono(6-NH_2)-β-CD against C4-HSLs; spectra of IDAs. Results for these HSLs could not be fitted satisfactorily. **B** Concentrations of target, host and indicator for IDAs of Mono(6-NH_2)-β-CD against oxo-C8-HSL and OH-C8-HSL; spectra of IDAs. Results for these HSLs could not be fitted satisfactorily. **C** IDA titration replicates of Mono(6-NH_2)-β-CD against H-C8-HSL; intensity traces at given wavelengths across the titration (inset: corresponding species' fluorescence emission spectra in the optimized model). The unbound 2,6-ANS spectrum was fixed, obtained from the corresponding host's oxo-C12-HSL averaged IDA model.

A

[Target], M	[Host], M	[Indicator], M
0	4E-5	2E-5
2E-5	4E-5	2E-5
1E-4	4E-5	2E-5
2E-4	4E-5	2E-5
4E-4	4E-5	2E-5
1E-3	4E-5	2E-5

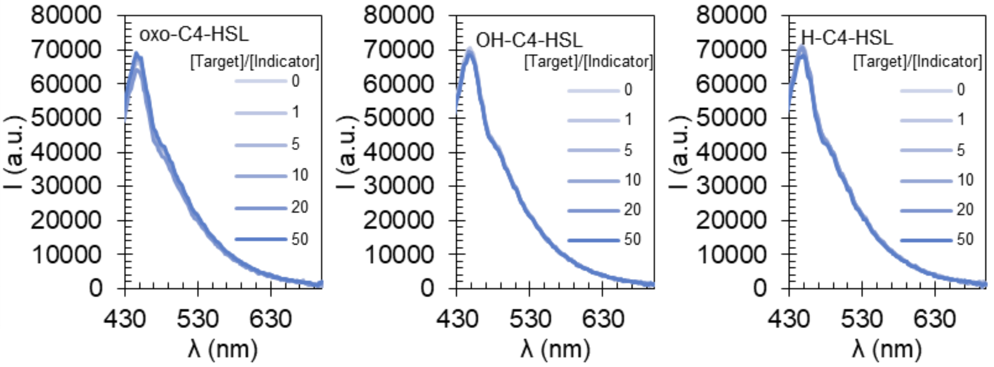

B

[Target], M	[Host], M	[Indicator], M
0	4E-5	2E-5
1E-5	4E-5	2E-5
2E-5	4E-5	2E-5
4E-5	4E-5	2E-5
1E-4	4E-5	2E-5
2E-4	4E-5	2E-5

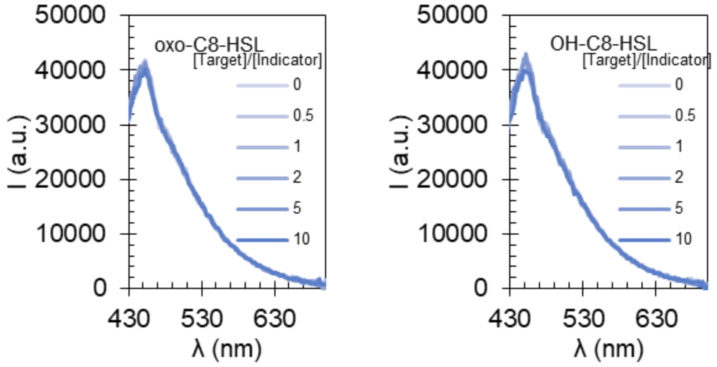

C

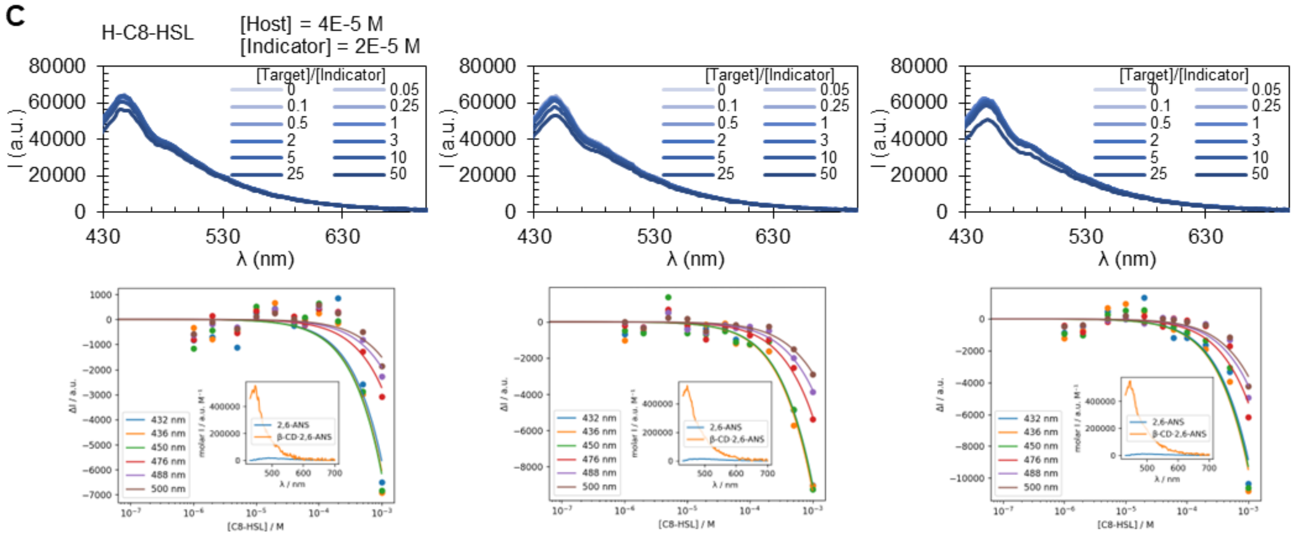

Figure S 50. **A** Concentrations of target, host and indicator for IDAs of Di(6-NH_2)-β-CD against C4-HSLs; spectra of IDAs. Results for these HSLs could not be fitted satisfactorily. **B** Concentrations of target, host and indicator for IDAs of Di(6-NH_2)-β-CD against oxo-C8-HSL and OH-C8-HSL; spectra of IDAs. Results for these HSLs could not be fitted satisfactorily. **C** IDA titration replicates of Di(6-NH_2)-β-CD against H-C8-HSL; intensity traces at given wavelengths across the titration (inset: corresponding species' fluorescence emission spectra in the optimized model).

A

[Target], M	[Host], M	[Indicator], M
0	4E-5	2E-5
2E-5	4E-5	2E-5
1E-4	4E-5	2E-5
2E-4	4E-5	2E-5
4E-4	4E-5	2E-5
1E-3	4E-5	2E-5

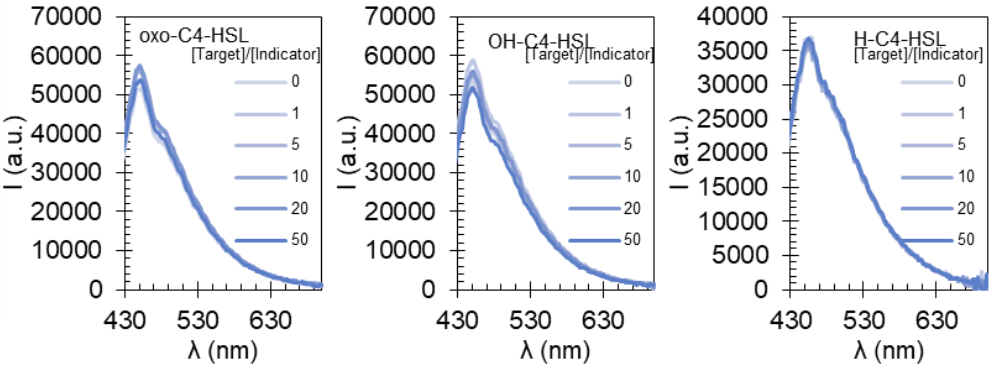

B

[Target], M	[Host], M	[Indicator], M
0	4E-5	2E-5
2E-5	4E-5	2E-5
1E-4	4E-5	2E-5
2E-4	4E-5	2E-5
4E-4	4E-5	2E-5
1E-3	4E-5	2E-5

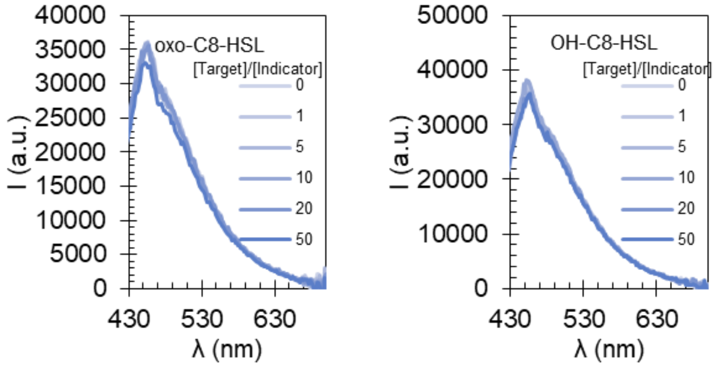

C

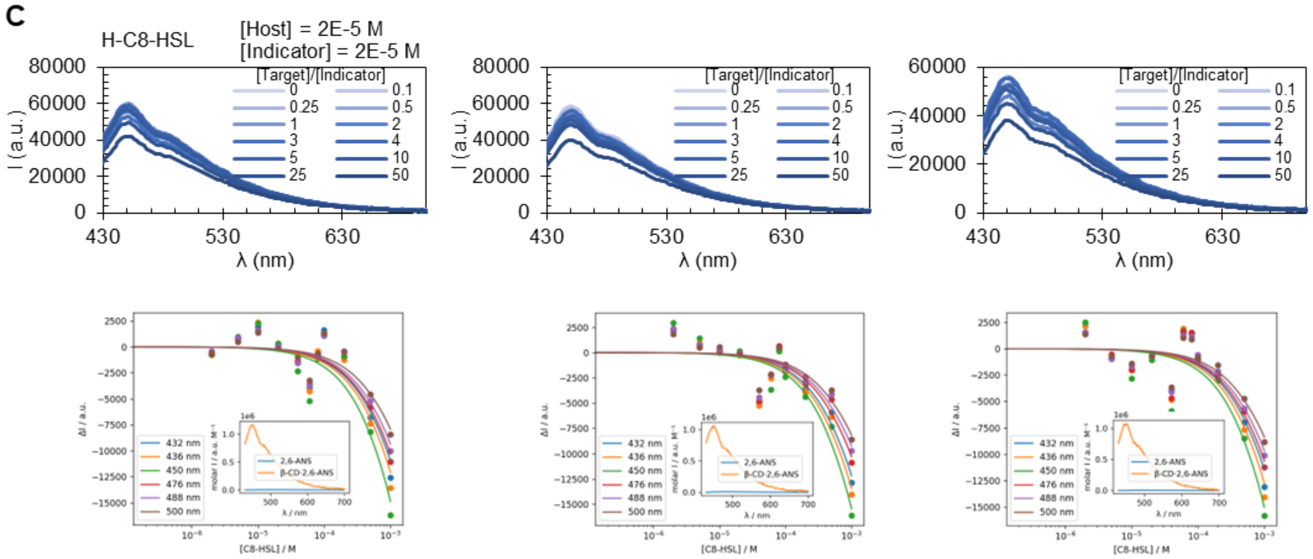

Figure S 51. **A** Concentrations of target, host and indicator for IDAs of MonoLys-β-CD against C4-HSLs; spectra of IDAs. Results for these HSLs could not be fitted satisfactorily. **B** Concentrations of target, host and indicator for IDAs of MonoLys-β-CD against oxo-C8-HSL and OH-C8-HSL; spectra of IDAs. Results for these HSLs could not be fitted satisfactorily. **C** IDA titration replicates of MonoLys-β-CD against H-C8-HSL; intensity traces at given wavelengths across the titration (inset: corresponding species' fluorescence emission spectra in the optimized model).

A

[Target], M	[Host], M	[Indicator], M
0	2E-5	2E-5
2E-5	2E-5	2E-5
1E-4	2E-5	2E-5
2E-4	2E-5	2E-5
4E-4	2E-5	2E-5
1E-3	2E-5	2E-5

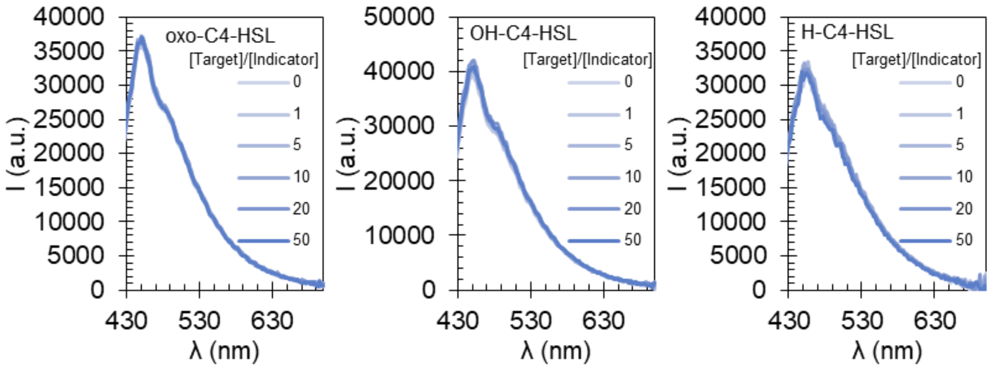

B

[Target], M	[Host], M	[Indicator], M
0	4E-5	2E-5
2E-5	4E-5	2E-5
1E-4	4E-5	2E-5
2E-4	4E-5	2E-5
4E-4	4E-5	2E-5
1E-3	4E-5	2E-5

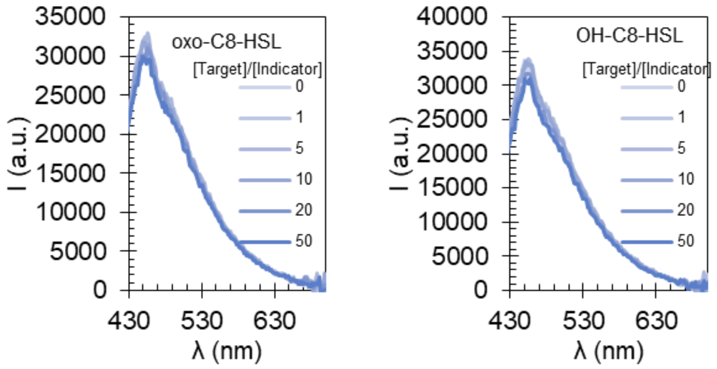

C

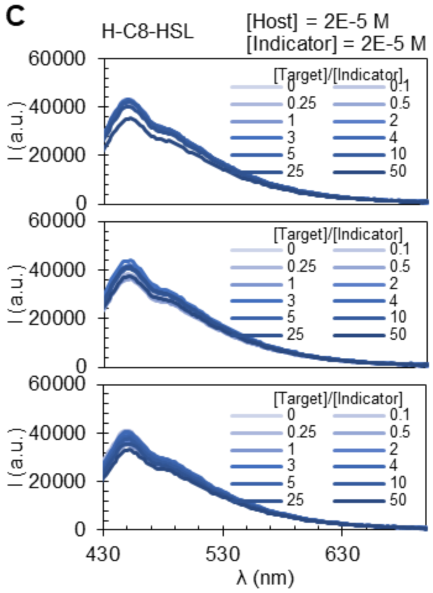

Figure S 52. **A** Concentrations of target, host and indicator for IDAs of DiLys-β-CD against C4-HSLs; spectra of IDAs. Results for these HSLs could not be fitted satisfactorily. **B** Concentrations of target, host and indicator for IDAs of DiLys-β-CD against oxo-C8-HSL and OH-C8-HSL; spectra of IDAs. Results for these HSLs could not be fitted satisfactorily. **C** IDA titration replicates of DiLys-β-CD against H-C8-HSL, concentrations and spectra. Results for these HSLs could not be fitted satisfactorily.

A

[Target], M	[Host], M	[Indicator], M
0	4E-5	2E-5
2E-5	4E-5	2E-5
1E-4	4E-5	2E-5
2E-4	4E-5	2E-5
4E-4	4E-5	2E-5
1E-3	4E-5	2E-5

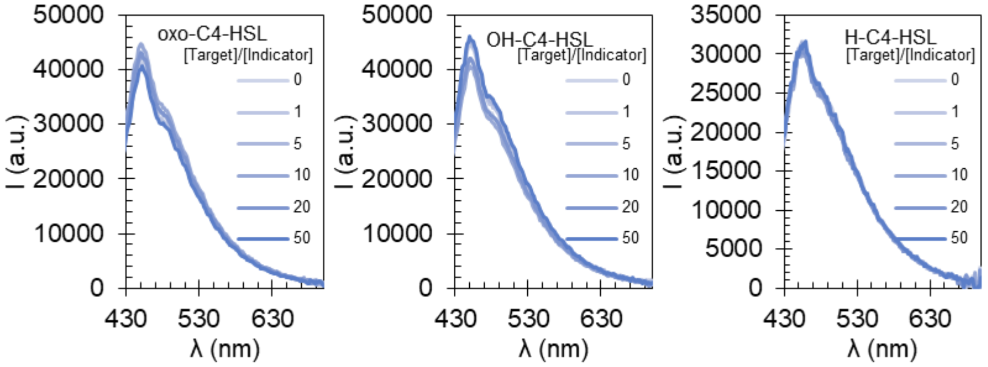

B

[Target], M	[Host], M	[Indicator], M
0	4E-5	2E-5
2E-5	4E-5	2E-5
1E-4	4E-5	2E-5
2E-4	4E-5	2E-5
4E-4	4E-5	2E-5
1E-3	4E-5	2E-5

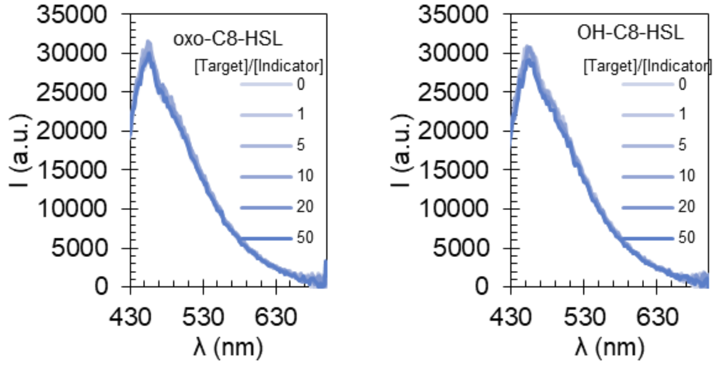

C

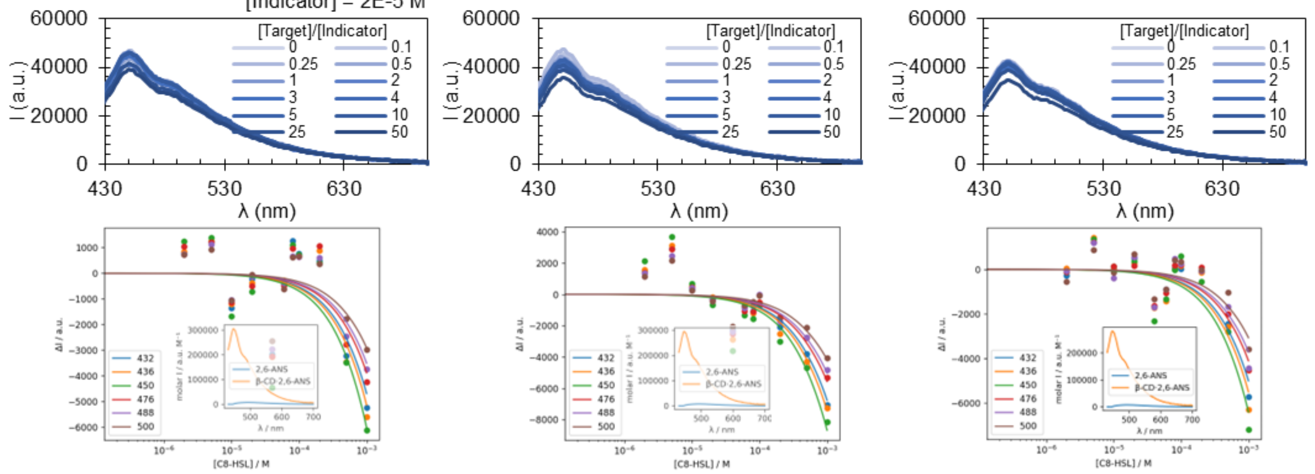

Figure S 53. **A** Concentrations of target, host and indicator for IDAs of Mono(6-TMA)-β-CD against C4-HSLs; spectra of IDAs. Results for these HSLs could not be fitted satisfactorily. **B** Concentrations of target, host and indicator for IDAs of Mono(TMA)-β-CD against oxo-C8-HSL and OH-C8-HSL; spectra of IDAs. Results for these HSLs could not be fitted satisfactorily. **C** IDA titration replicates of Mono(TMA)-β-CD against H-C8-HSL; intensity traces at given wavelengths across the titration (small panels: corresponding species' fluorescence emission spectra in the optimized model). The unbound 2,6-ANS spectrum was fixed, obtained from the corresponding host's OH-C12-HSL averaged IDA model.

A

[Target], M	[Host], M	[Indicator], M
0	4E-5	2E-5
2E-5	4E-5	2E-5
1E-4	4E-5	2E-5
2E-4	4E-5	2E-5
4E-4	4E-5	2E-5
1E-3	4E-5	2E-5

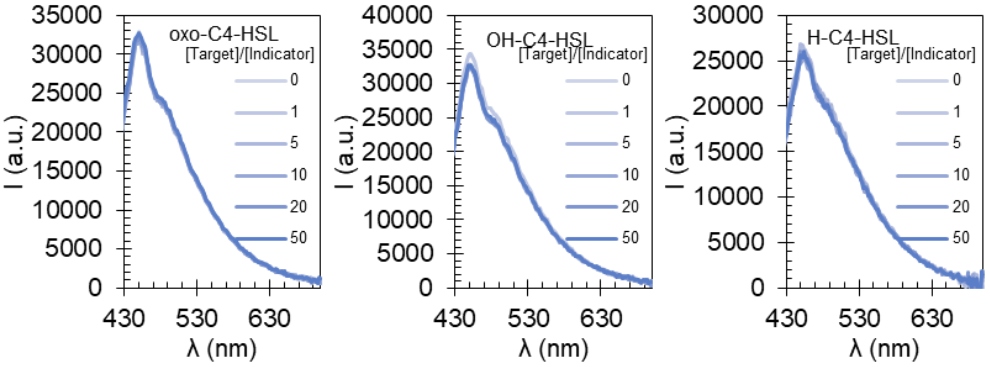

B

[Target], M	[Host], M	[Indicator], M
0	4E-5	2E-5
2E-5	4E-5	2E-5
1E-4	4E-5	2E-5
2E-4	4E-5	2E-5
4E-4	4E-5	2E-5
1E-3	4E-5	2E-5

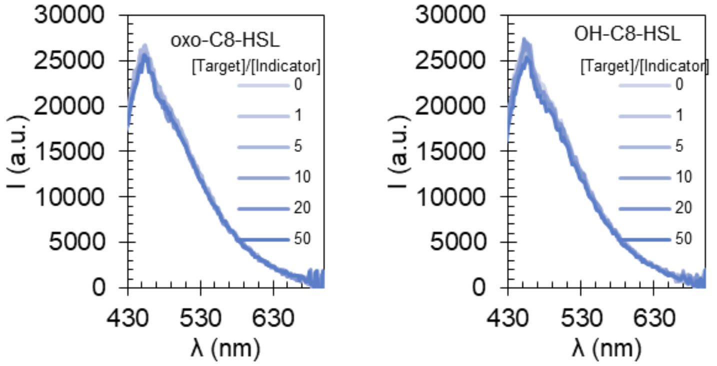

C

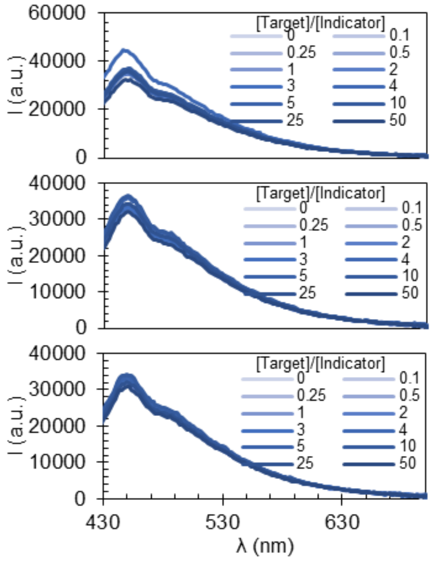

H-C8-HSL
[Host] = 2E-5 M
[Indicator] = 2E-5 M

Figure S 54. **A** Concentrations of target, host and indicator for IDAs of Di(TMA)-β-CD against C4-HSLs; spectra of IDAs. Results for these HSLs could not be fitted satisfactorily. **B** Concentrations of target, host and indicator for IDAs of Di(TMA)-β-CD against oxo-C8-HSL and OH-C8-HSL; spectra of IDAs. Results for these HSLs could not be fitted satisfactorily. **C** IDA titration replicates of Di(TMA)-β-CD against H-C8-HSL, concentrations and spectra. Results for these HSLs could not be fitted satisfactorily.

A

[Target], M	[Host], M	[Indicator], M
0	2E-5	2E-5
2E-5	2E-5	2E-5
1E-4	2E-5	2E-5
2E-4	2E-5	2E-5
4E-4	2E-5	2E-5
1E-3	2E-5	2E-5

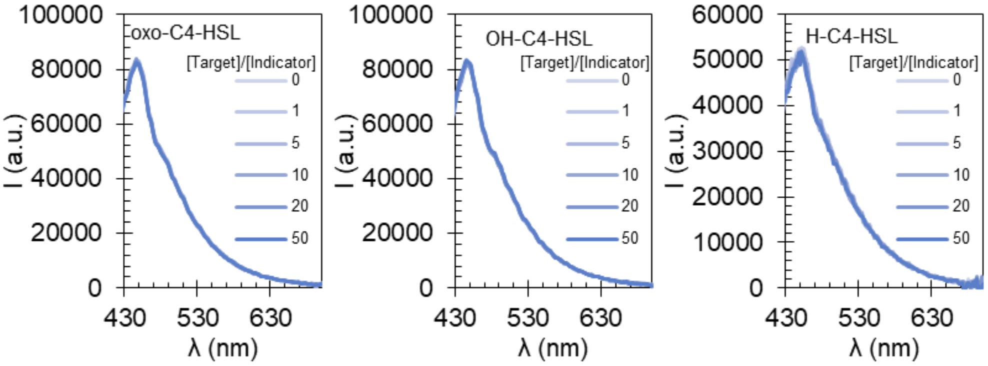

B

[Target], M	[Host], M	[Indicator], M
0	2E-5	2E-5
2E-5	2E-5	2E-5
1E-4	2E-5	2E-5
2E-4	2E-5	2E-5
4E-4	2E-5	2E-5
1E-3	2E-5	2E-5

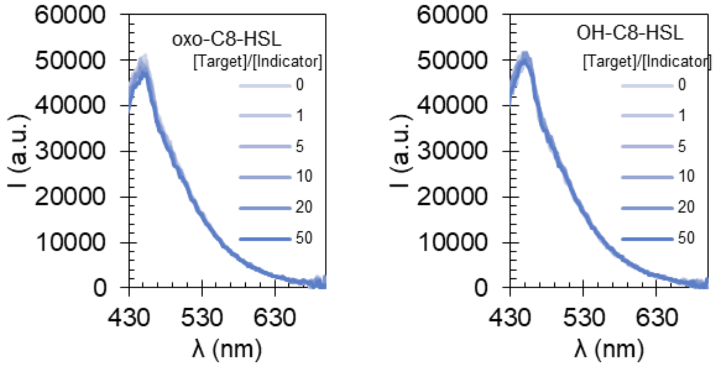

C

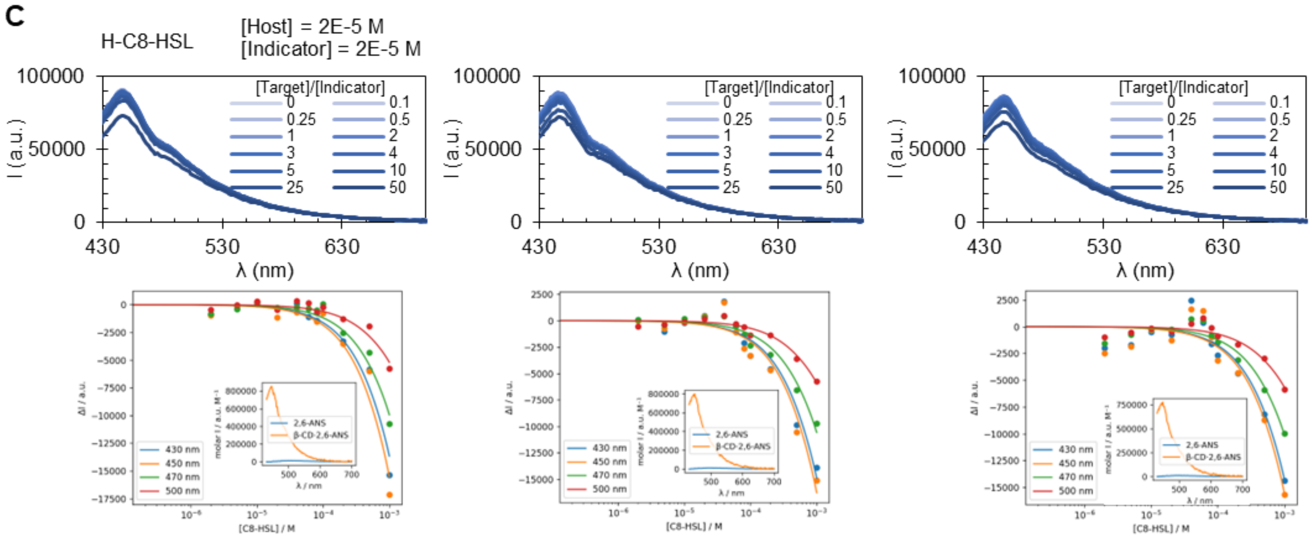

Figure S 55. **A** Concentrations of target, host and indicator for IDAs of 2,6-DiMe-β-CD against C4-HSLs; spectra of IDAs. Results for these HSLs could not be fitted satisfactorily. **B** Concentrations of target, host and indicator for IDAs of 2,6-DiMe-β-CD against oxo-C8-HSL and OH-C8-HSL; spectra of IDAs. Results for these HSLs could not be fitted satisfactorily. **C** IDA titration replicates of 2,6-DiMe-β-CD against H-C8-HSL; intensity traces at given wavelengths across the titration (small panels: corresponding species' fluorescence emission spectra in the optimized model).

A

[Target], M	[Host], M	[Indicator], M
0	4E-5	2E-5
2E-5	4E-5	2E-5
1E-4	4E-5	2E-5
2E-4	4E-5	2E-5
4E-4	4E-5	2E-5
1E-3	4E-5	2E-5

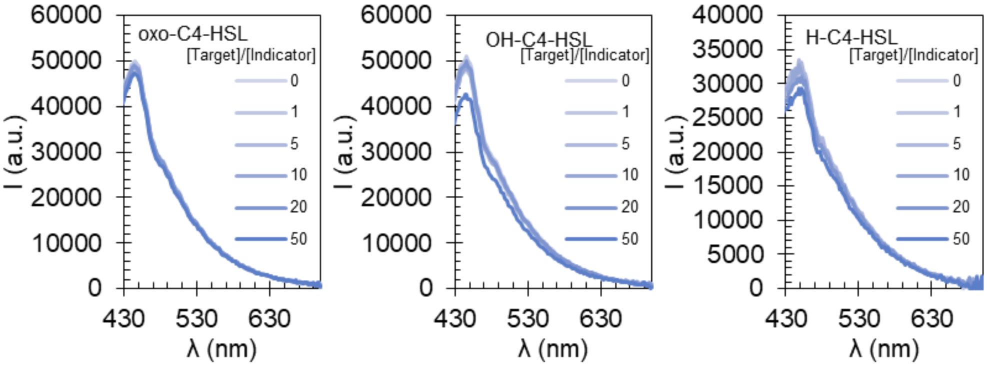

B

[Target], M	[Host], M	[Indicator], M
0	4E-5	2E-5
2E-5	4E-5	2E-5
1E-4	4E-5	2E-5
2E-4	4E-5	2E-5
4E-4	4E-5	2E-5
1E-3	4E-5	2E-5

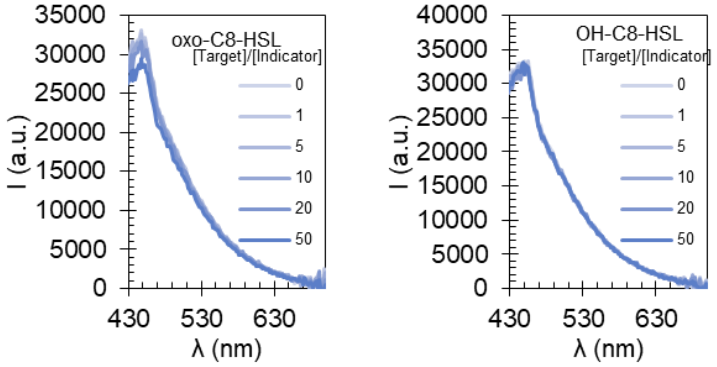

C

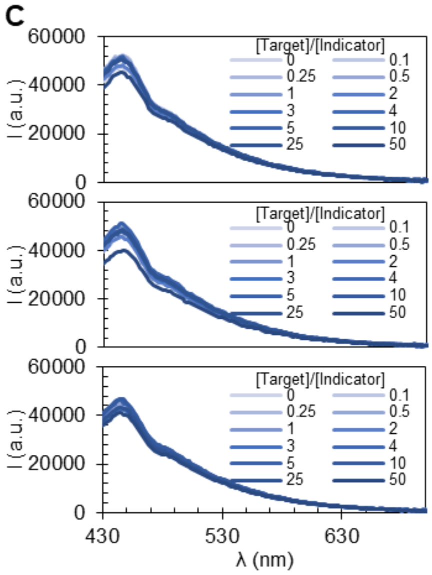

H-C8-HSL

[Host] = 2E-5 M

[Indicator] = 2E-5 M

Figure S 56. **A** Concentrations of target, host and indicator for IDAs of TriMe-β-CD against C4-HSLs; spectra of IDAs. Results for these HSLs could not be fitted satisfactorily. **B** Concentrations of target, host and indicator for IDAs of TriMe-β-CD against oxo-C8-HSL and OH-C8-HSL; spectra of IDAs. Results for these HSLs could not be fitted satisfactorily. **C** IDA titration replicates of TriMe-β-CD against H-C8-HSL, concentrations and spectra. Results for these HSLs could not be fitted satisfactorily.

The addition of H-C12-HSL in IDAs involving lysinated and TMA-substituted β-CDs led to an unexpected increase in fluorescence intensity (Figures S42–S45). This goes against the expected stagnation of fluorescence for a low binding, and expected decrease of fluorescence for high enough binding to out-compete the indicator for inclusion. The unexpected evolution of fluorescence with some hosts may be related to the formation of secondary structures or aggregates that provide a "less polar" environment to the indicator. For instance, H-C12-HSL has been reported to form micelles in a sucrose Tris buffer at 37°C at micromolar concentrations[30]. However, there is a lack of a similar effect in a control with increasing H-C12-HSL together with 2,6-ANS at a constant concentration (Supplementary Figure S29). That is, the hosts are seemingly required along H-C12-HSL and 2,6-ANS to produce the anomalous fluorescence increase.

Table S 2. Binding constants obtained between hosts and HSLs by IDA with 2,6-ANS as indicator.

H-C8-HSL					
**Host**	**Ka's (M^{-1})**	**$n_{replicates}$**	**Average**	**Log(K_a)**	**StDev**
β-CD	386, 360, 403	3	383.0	2.58	0.02
Mono(6-NH_2)-β-CD	598, 626, 452	3	558.7	2.75	0.07
Di(6-NH_2)-β-CD	142, 256, 248	3	215.3	2.33	0.13
2,6-DiMe-β-CD	277, 320, 318	3	305.0	2.48	0.03
TriMe-β-CD	-	-	-	-	-
Lys-β-CD	386, 472, 462	3	440.0	2.64	0.05
Di-Lys-β-CD	-	-	-	-	-
Mono(6-TMA)-β-CD	221, 362, 263	3	282.0	2.45	0.11
Di(6-TMA)-β-CD	-	-	-	-	-
**oxo-C12-HSL**					
**Host**	**Ka's (M^{-1})**	**$n_{replicates}$**	**Average**	**Log(K_a)**	**StDev**
β-CD	7900, 5900, 6860	3	6886.7	3.84	0.06
Mono(6-NH_2)-β-CD	20200, 9730, 12200	3	14043.3	4.15	0.17
Di(6-NH_2)-β-CD	4390, 3770, 4440	3	4200.0	3.62	0.04
2,6-DiMe-β-CD	5400, 4680, 4770	3	4950.0	3.69	0.03
TriMe-β-CD	-	-	-	-	-
Lys-β-CD	6310, 3890, 5090	3	5096.7	3.71	0.10
Di-Lys-β-CD	3240, 6120, 2840	3	4066.7	3.61	0.19
Mono(6-TMA)-β-CD	5130, 4910, 4140	3	4726.7	3.67	0.05
Di(6-TMA)-β-CD	1790, 14600, 7980	3	8123.3	3.91	0.34
**OH-C12-HSL**					
**Host**	**Ka's (M^{-1})**	**$n_{replicates}$**	**Average**	**Log(K_a)**	**StDev**
β-CD	8840, 9480, 8980	3	9100.0	3.96	0.02
Mono(6-NH_2)-β-CD	8190, 10200, 8130	3	8840.0	3.95	0.06
Di(6-NH_2)-β-CD	2790, 2890, 3400	3	3026.7	3.48	0.05
2,6-DiMe-β-CD	5640, 5770, 5640	3	5683.3	3.75	0.01
TriMe-β-CD	-	-	-	-	-
Lys-β-CD	4890, 4860, 5040	3	4930.0	3.69	0.01
Di-Lys-β-CD	2570, 3730, 1990	3	2763.3	3.44	0.14
Mono(6-TMA)-β-CD	4950, 5300, 3940	3	4730.0	3.67	0.06
Di(6-TMA)-β-CD	1060, 1510, 868	3	1146.0	3.06	0.12
**H-C12-HSL**					
**Host**	**Ka's (M^{-1})**	**$n_{replicates}$**	**Average**	**Log(K_a)**	**StDev**
β-CD	2080, 1430, 1490	3	1666.7	3.22	0.09
Mono(6-NH_2)-β-CD	2050, 1430, 1610	3	1696.7	3.23	0.08
Di(6-NH_2)-β-CD	1670, 1530, 1250	3	1483.3	3.17	0.06
2,6-DiMe-β-CD	1520, 1330, 1150	3	1333.3	3.12	0.06
TriMe-β-CD	-	-	-	-	-
Lys-β-CD	-	-	-	-	-
Di-Lys-β-CD	-	-	-	-	-
Mono(6-TMA)-β-CD	-	-	-	-	-
Di(6-TMA)-β-CD	-	-	-	-	-

3.4. ROESY NMR

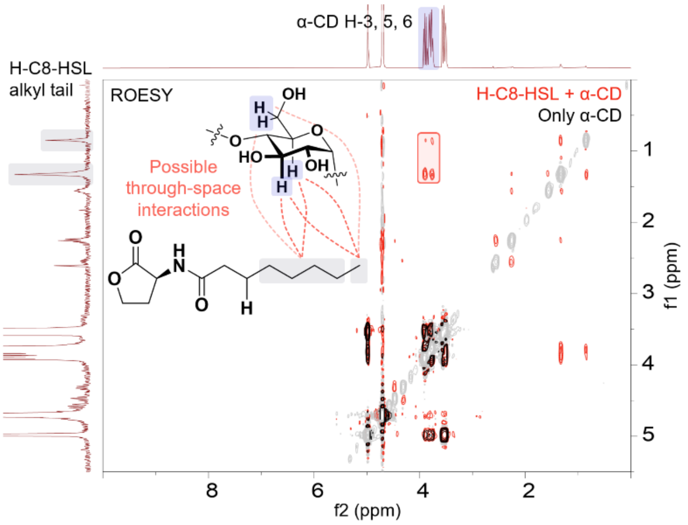

Figure S 57. ROESY NMR spectrum of α-CD alone (black) and mixed with H-C8-HSL (red). Self-correlations are shown in grey. [α-CD]=20 mM, [H-C8-HSL]=0.33 mM, in 1% DMSO-d_6/D_2O. ^{1}H NMR projections correspond to the H-C8-HSL + α-CD sample, with denoted signals of interest. Inset shows through-space interactions that are possible based on the correlated groups of peaks.

4. References

(1) Soloviev, D. O.; Hunter, C. A. Musketeer: A Software Tool for the Analysis of Titration Data. *Chem. Sci.* **2024**, *15* (37), 15299–15310. https://doi.org/10.1039/D4SC03354J.
(2) Scheuermann, T. H.; Brautigam, C. A. High-Precision, Automated Integration of Multiple Isothermal Titration Calorimetric Thermograms: New Features of NITPIC. *Methods* **2015**, *76*, 87–98. https://doi.org/10.1016/j.ymeth.2014.11.024.
(3) Keller, S.; Vargas, C.; Zhao, H.; Piszczek, G.; Brautigam, C. A.; Schuck, P. High-Precision Isothermal Titration Calorimetry with Automated Peak-Shape Analysis. *Anal. Chem.* **2012**, *84* (11), 5066–5073. https://doi.org/10.1021/ac3007522.
(4) Zhao, H.; Piszczek, G.; Schuck, P. SEDPHAT – A Platform for Global ITC Analysis and Global Multi-Method Analysis of Molecular Interactions. *Methods* **2015**, *76*, 137–148. https://doi.org/10.1016/j.ymeth.2014.11.012.
(5) Nudelman, A.; Bechor ,Yosi; Falb ,Eliezer; Fischer ,Bilha; Wexler ,Barry A.; and Nudelman, A. Acetyl Chloride-Methanol as a Convenient Reagent for: A) Quantitative Formation of Amine Hydrochlorides B) Carboxylate Ester Formation C) Mild Removal of N-t-Boc-Protective Group. *Synth. Commun.* **1998**, *28* (3), 471–474. https://doi.org/10.1080/00397919808005101.
(6) Davis, B. M.; Richens, J. L.; O'Shea, P. Label-Free Critical Micelle Concentration Determination of Bacterial Quorum Sensing Molecules. *Biophys. J.* **2011**, *101* (1), 245–254. https://doi.org/10.1016/j.bpj.2011.05.033.
